# Supplementary material for: A Symmetric Prior for the Regularisation of Elastic Deformations: Improved anatomical plausibility in nonlinear image registration
Source: Neuroimage. 2020 Oct 1;219:116962. doi: 10.1016/j.neuroimage.2020.116962 (PMC7610794; doi:10.1016/j.neuroimage.2020.116962)
Supplement: Multimedia component 3 [file mmc3.pdf]

A Symmetric Prior for the Regularisation of Elastic Deformations: Improved Anatomical Plausibility in Nonlinear Image Registration - *Supplementary Material: Comparison of Log-Jacobian Determinant Spatial Maps for all Subject Pairs in the NIREP Dataset*

Frederik J Lange[1,], John Ashburner[1], Stephen M Smith[1], Jesper L R Andersson[1]

[a]*Centre for Functional MRI of the Brain (FMRIB), Wellcome Centre for Integrative Neuroimaging, Nuffield Department of Clinical Neurosciences, University of Oxford, John Radcliffe Hospital, Headley Way, Oxford, OX3 9DU, UK*

[b]*Wellcome Centre for Human Neuroimaging, UCL Institute of Neurology, University College London, 12 Queen Square, London, WC1N 3BG, UK*

1. Reference Subject 01

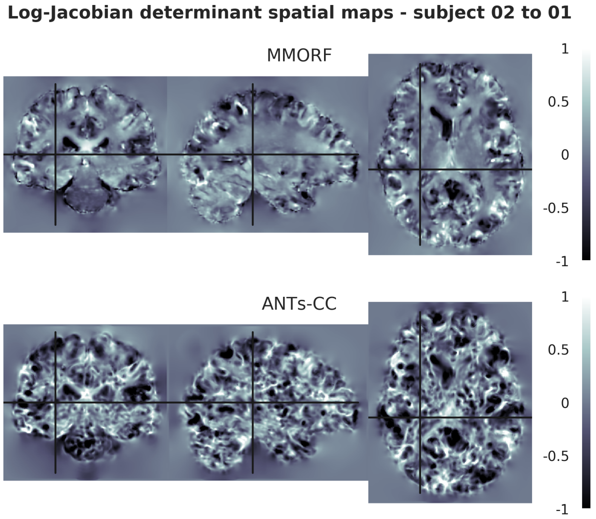

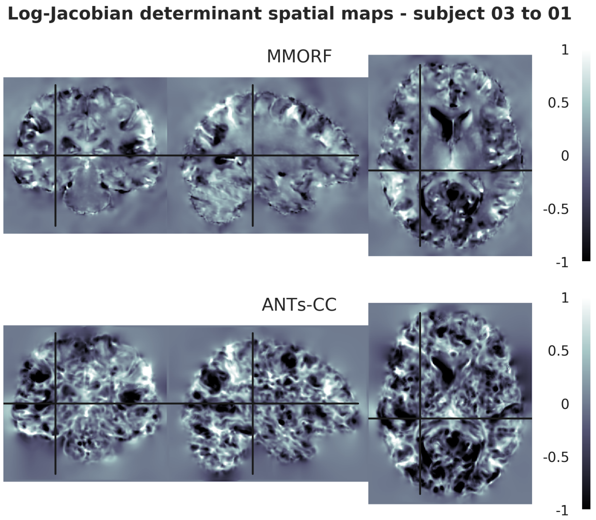

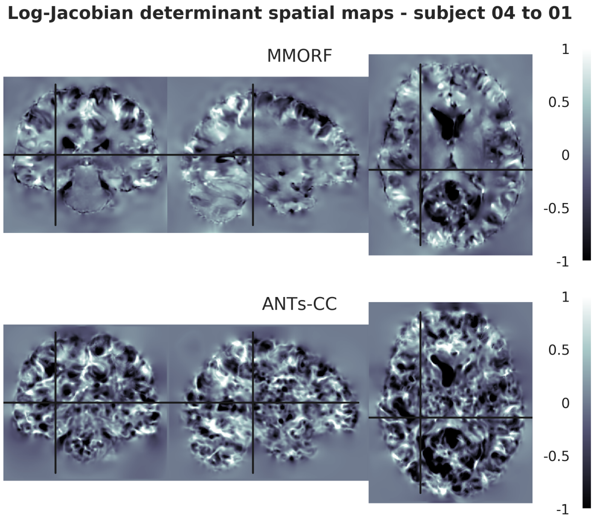

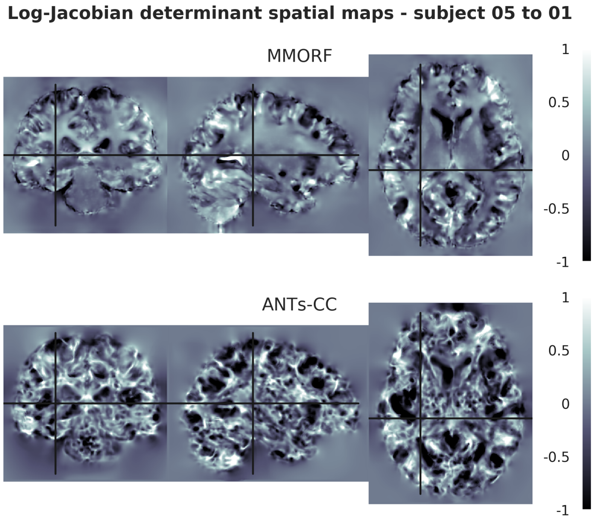

*Corresponding Author

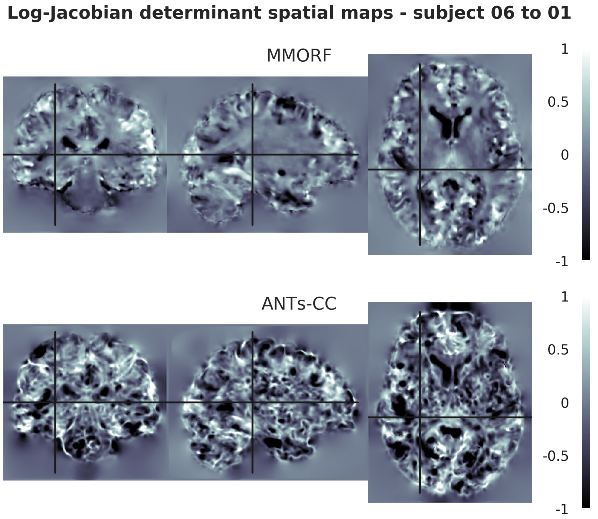
Log-Jacobian determinant spatial maps - subject 06 to 01
MMORF
ANTs-CC
1
0.5
0
-0.5
-1

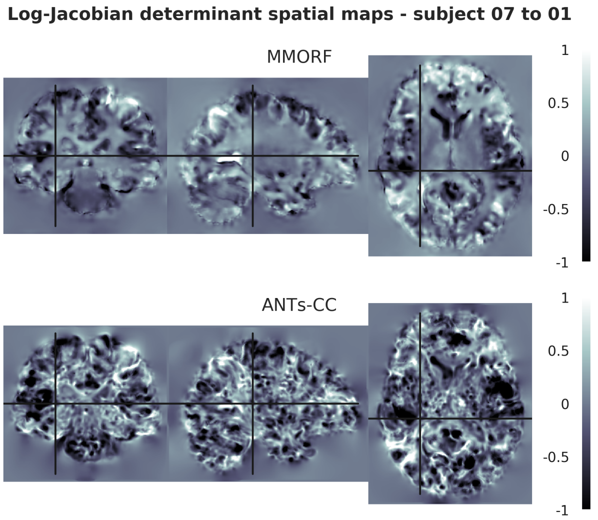
Log-Jacobian determinant spatial maps - subject 07 to 01
MMORF
ANTs-CC
1
0.5
0
-0.5
-1

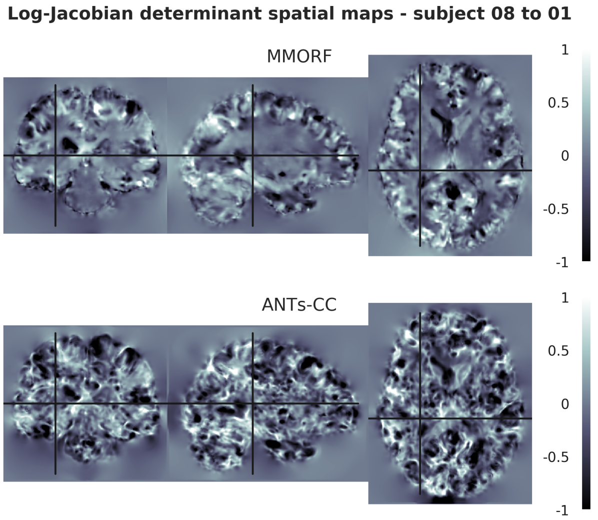
Log-Jacobian determinant spatial maps - subject 08 to 01
MMORF
ANTs-CC
1
0.5
0
-0.5
-1

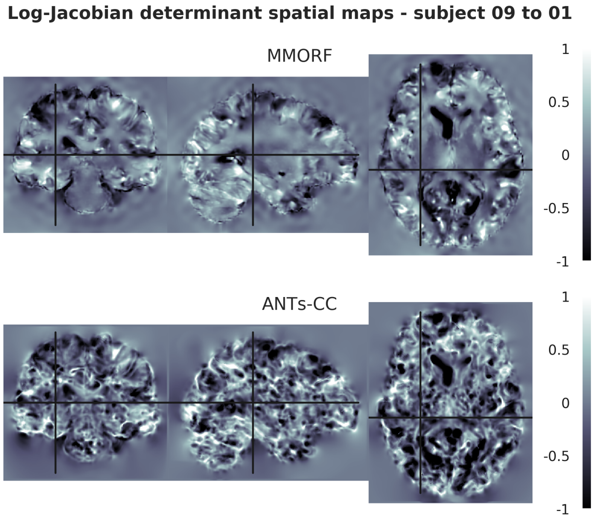
Log-Jacobian determinant spatial maps - subject 09 to 01
MMORF
ANTs-CC
1
0.5
0
-0.5
-1

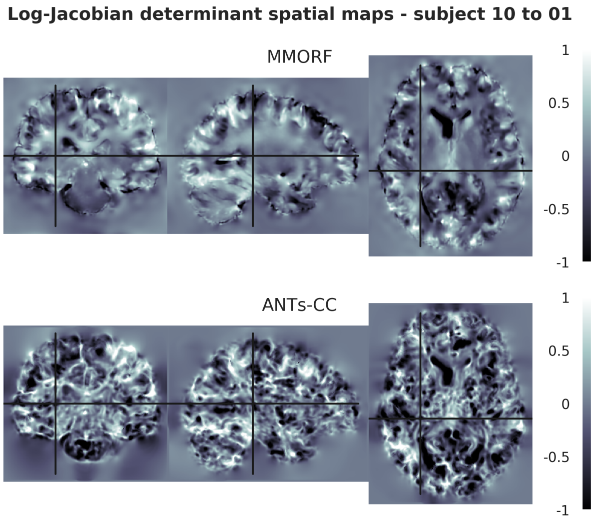
Log-Jacobian determinant spatial maps - subject 10 to 01
MMORF
ANTs-CC
1
0.5
0
-0.5
-1

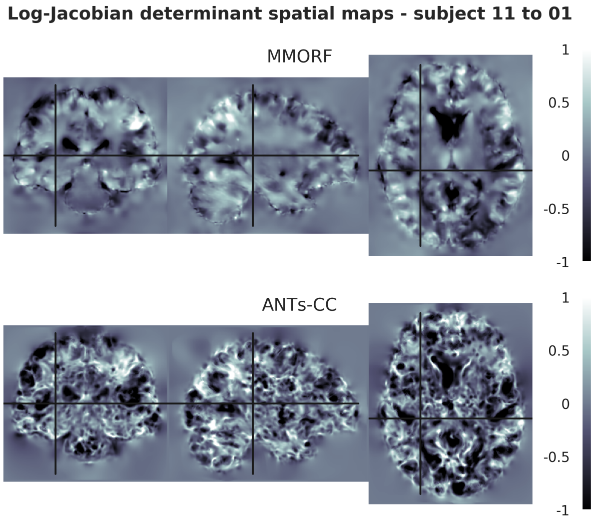
Log-Jacobian determinant spatial maps - subject 11 to 01
MMORF
ANTs-CC
1
0.5
0
-0.5
-1

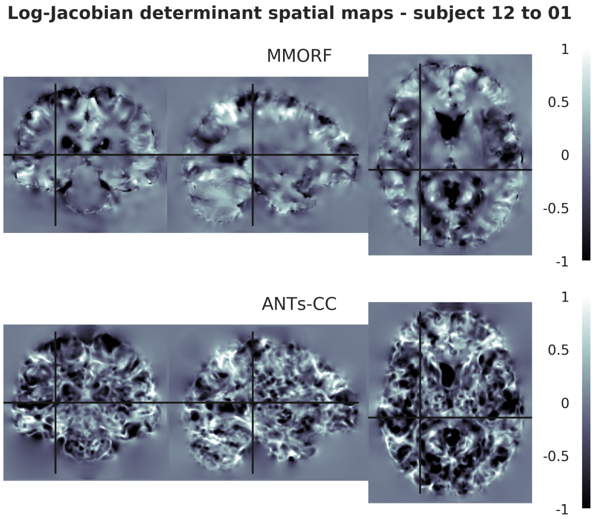
Log-Jacobian determinant spatial maps - subject 12 to 01
MMORF
ANTs-CC
1
0.5
0
-0.5
-1

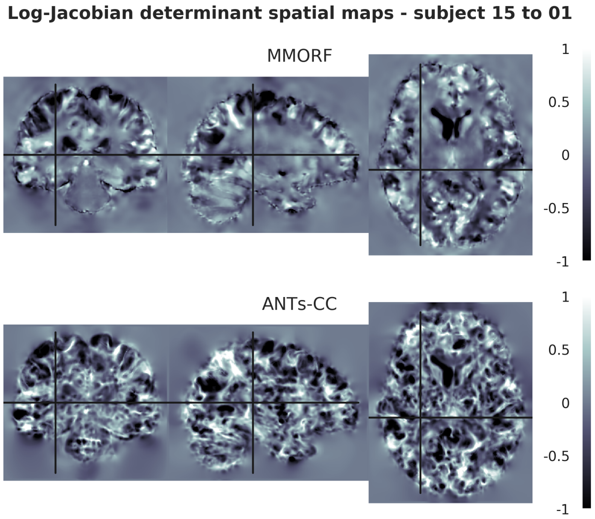
Log-Jacobian determinant spatial maps - subject 15 to 01
MMORF
ANTs-CC
1
0.5
0
-0.5
-1

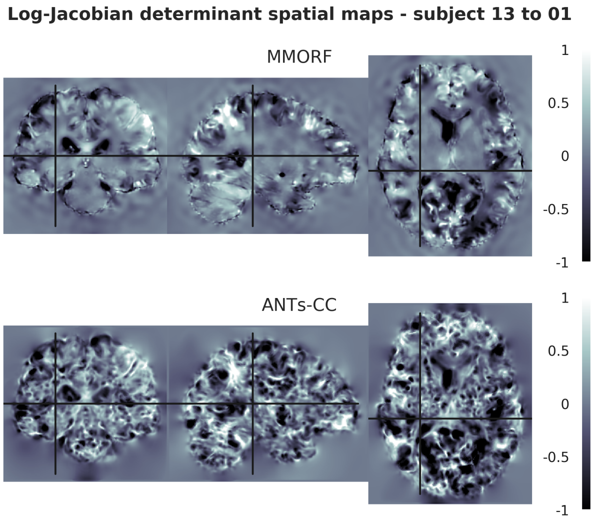
Log-Jacobian determinant spatial maps - subject 13 to 01
MMORF
ANTs-CC
1
0.5
0
-0.5
-1

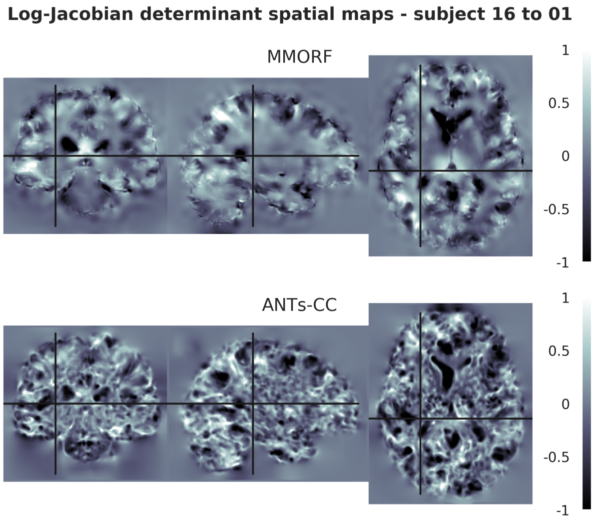
Log-Jacobian determinant spatial maps - subject 16 to 01
MMORF
ANTs-CC
1
0.5
0
-0.5
-1

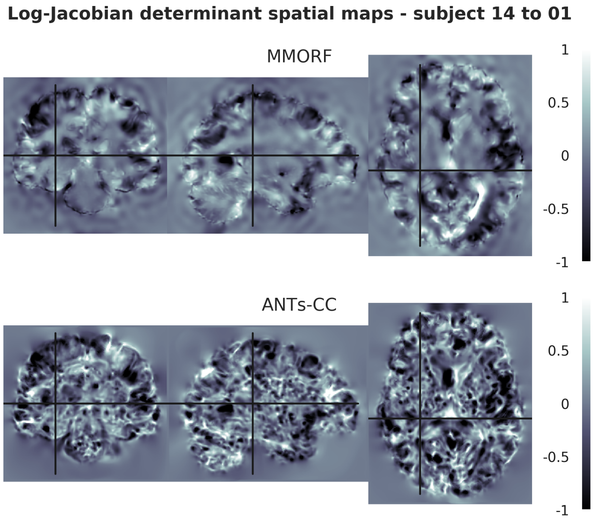
Log-Jacobian determinant spatial maps - subject 14 to 01
MMORF
ANTs-CC
1
0.5
0
-0.5
-1

2. Reference Subject 02

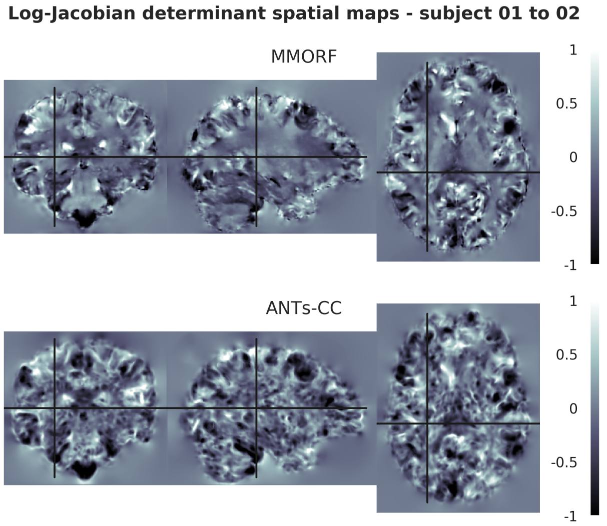

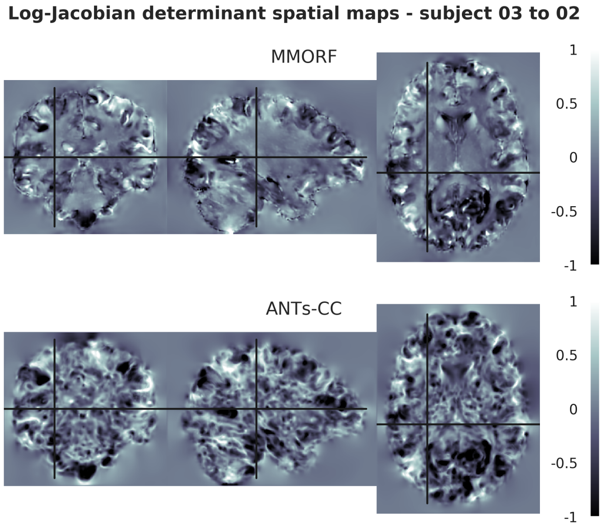

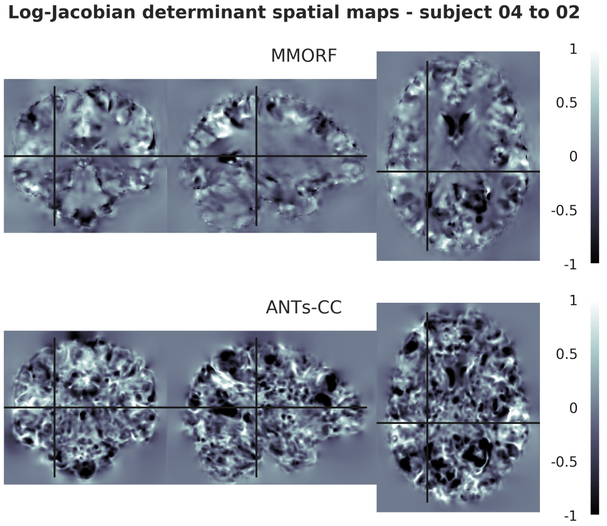

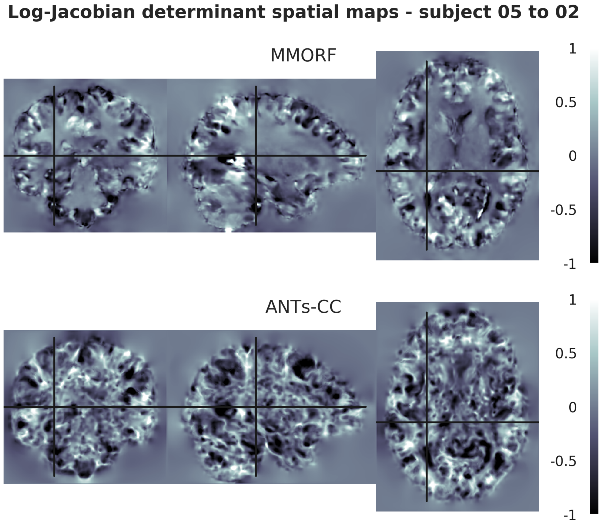

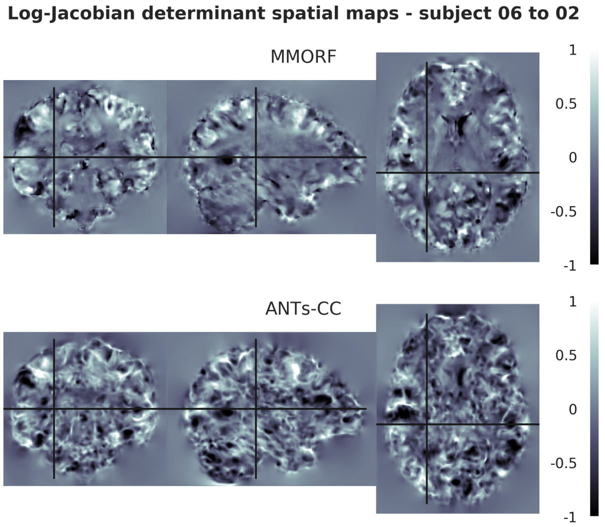

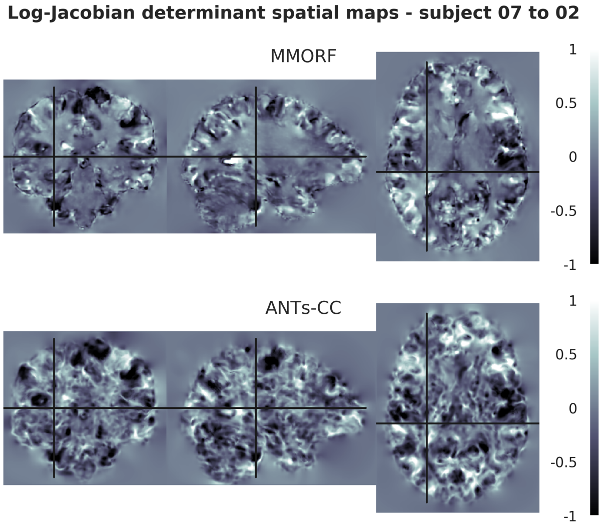

Log-Jacobian determinant spatial maps - subject 08 to 02

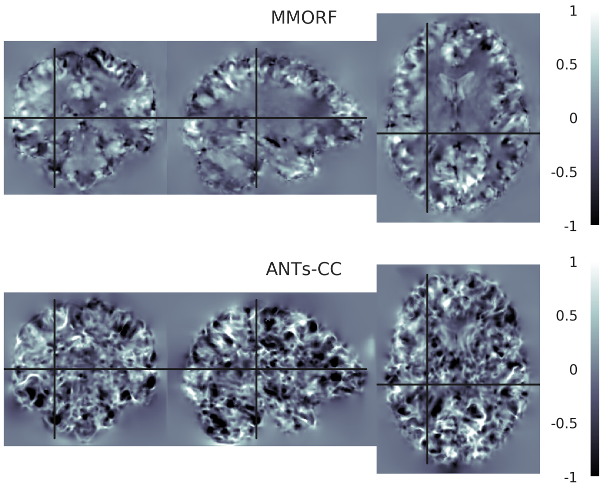

Log-Jacobian determinant spatial maps - subject 09 to 02

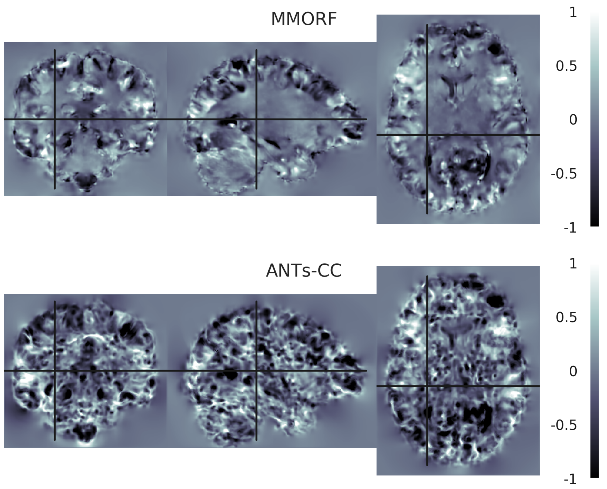

Log-Jacobian determinant spatial maps - subject 10 to 02

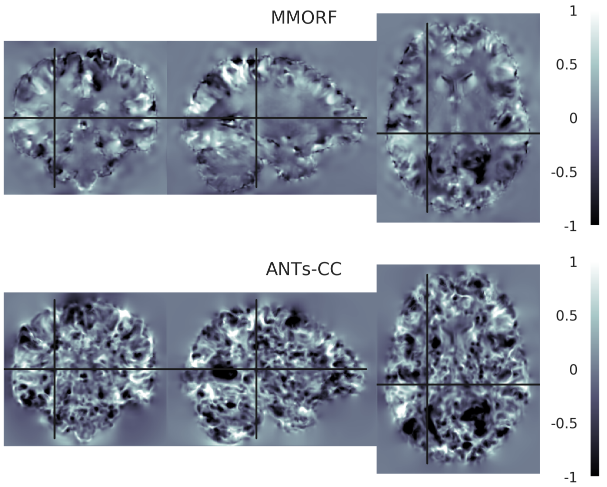

Log-Jacobian determinant spatial maps - subject 11 to 02

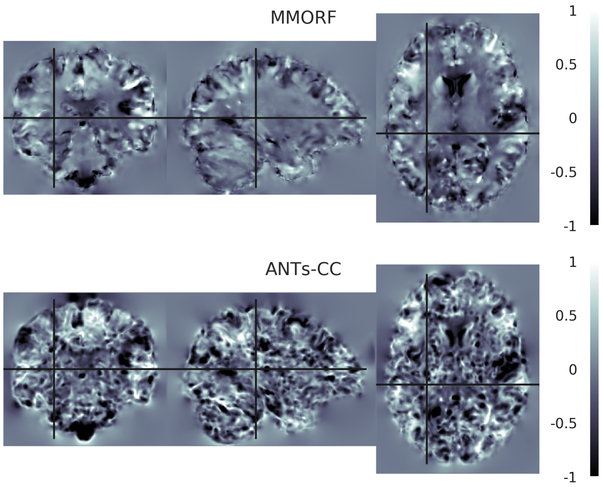

Log-Jacobian determinant spatial maps - subject 12 to 02

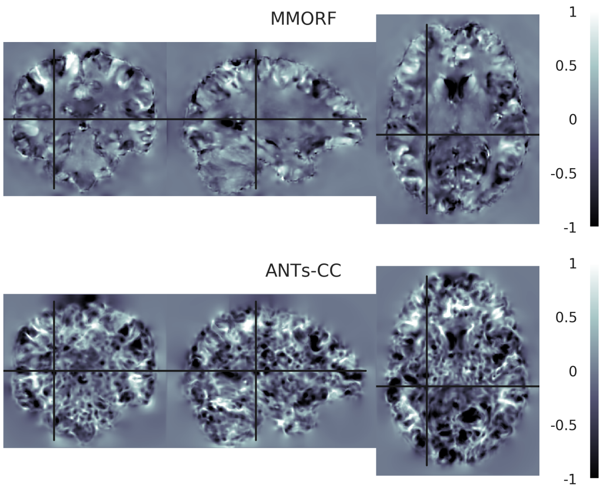

Log-Jacobian determinant spatial maps - subject 13 to 02

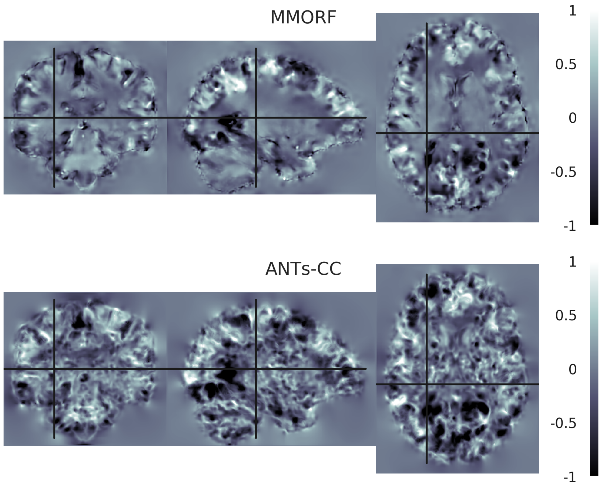

Log-Jacobian determinant spatial maps - subject 14 to 02

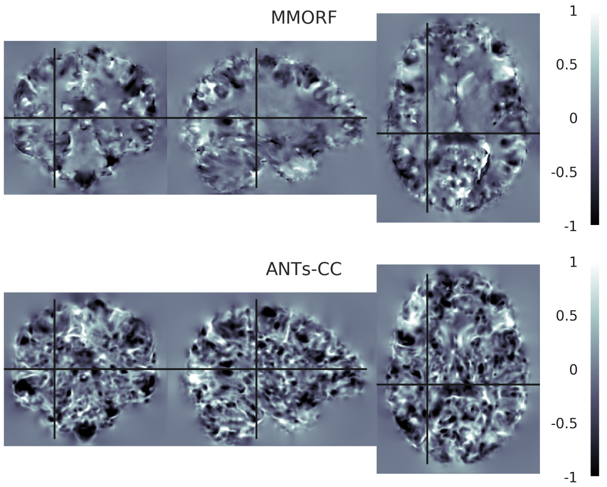

Log-Jacobian determinant spatial maps - subject 15 to 02

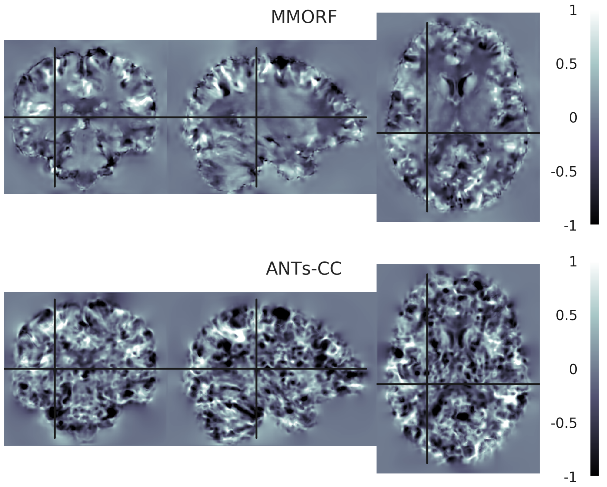

Log-Jacobian determinant spatial maps - subject 16 to 02

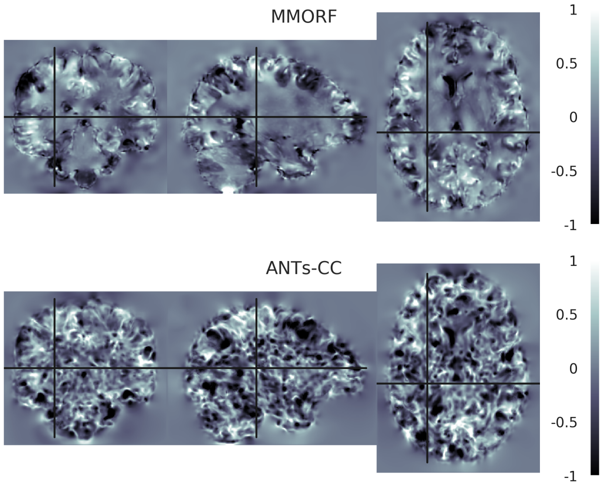

3. Reference Subject 03

Log-Jacobian determinant spatial maps - subject 01 to 03

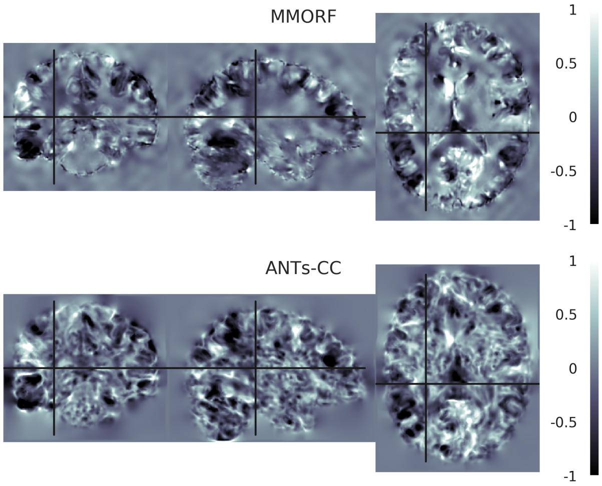

Log-Jacobian determinant spatial maps - subject 02 to 03

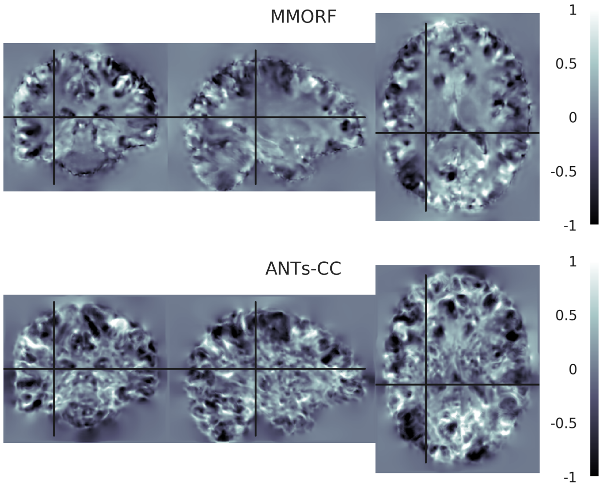

Log-Jacobian determinant spatial maps - subject 04 to 03

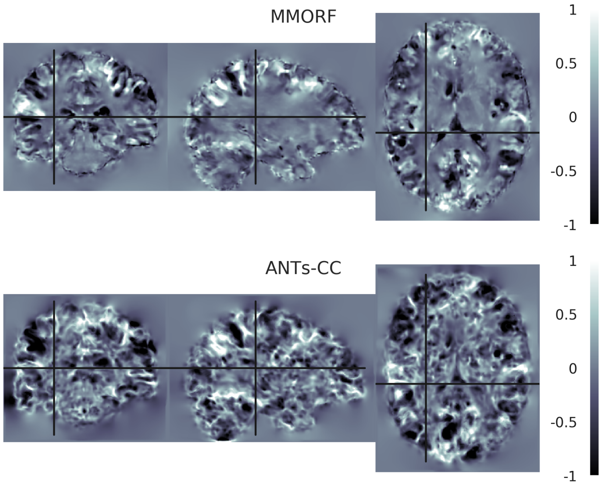

Log-Jacobian determinant spatial maps - subject 05 to 03

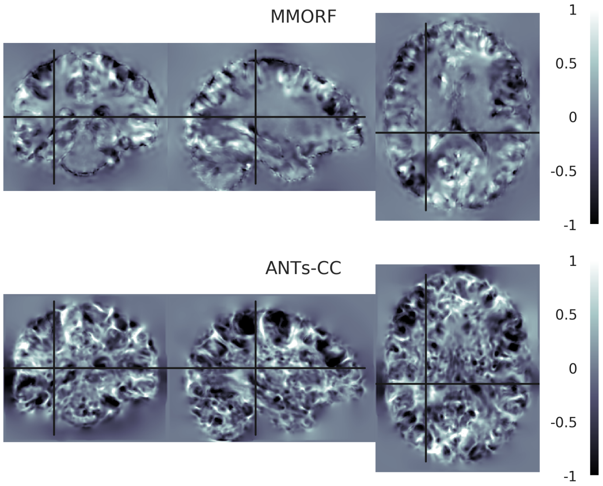

Log-Jacobian determinant spatial maps - subject 06 to 03

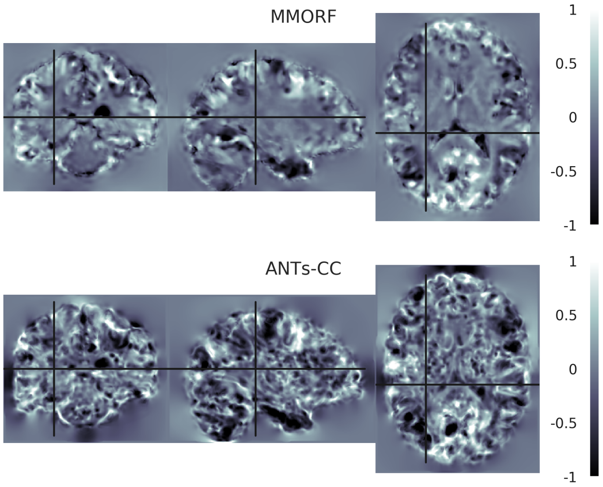

Log-Jacobian determinant spatial maps - subject 07 to 03

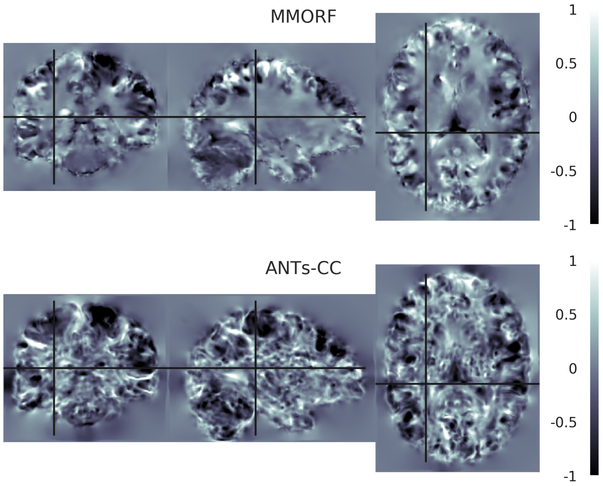

Log-Jacobian determinant spatial maps - subject 08 to 03

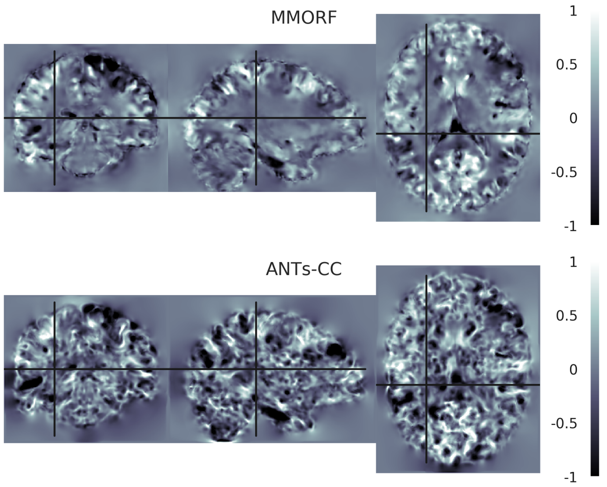

Log-Jacobian determinant spatial maps - subject 09 to 03

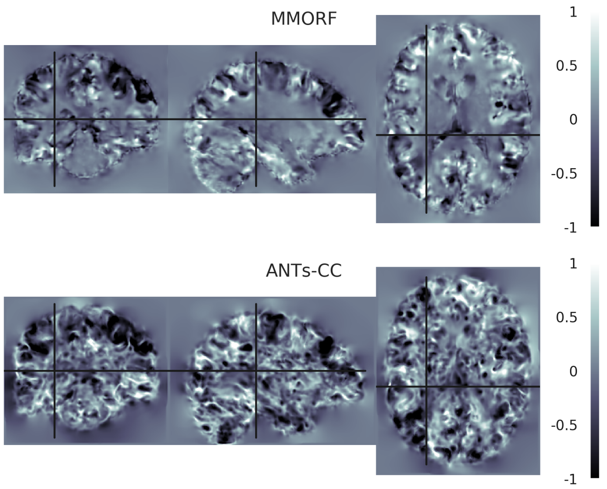

Log-Jacobian determinant spatial maps - subject 10 to 03

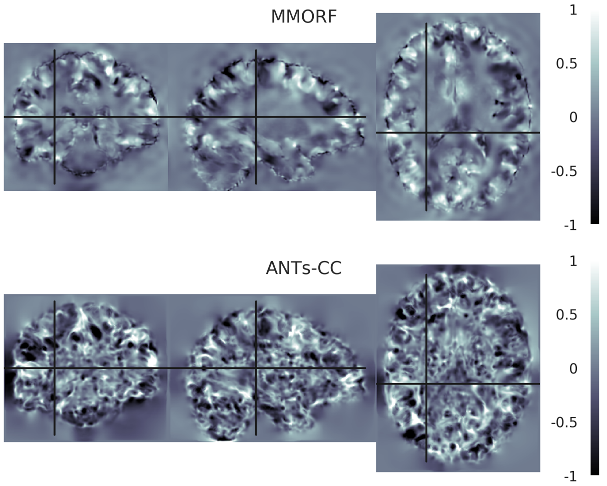

Log-Jacobian determinant spatial maps - subject 11 to 03

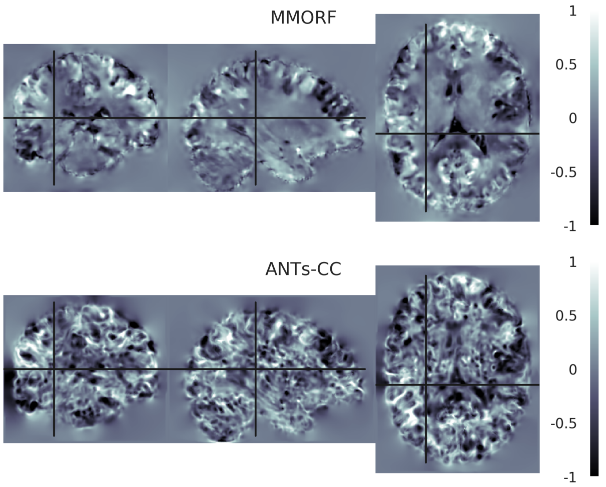

Log-Jacobian determinant spatial maps - subject 12 to 03

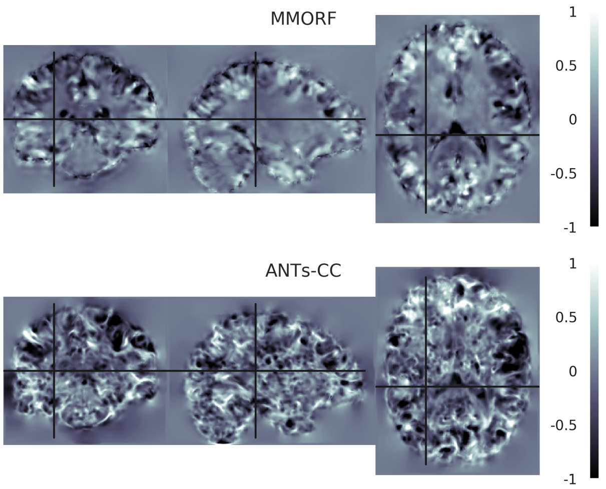

Log-Jacobian determinant spatial maps - subject 13 to 03

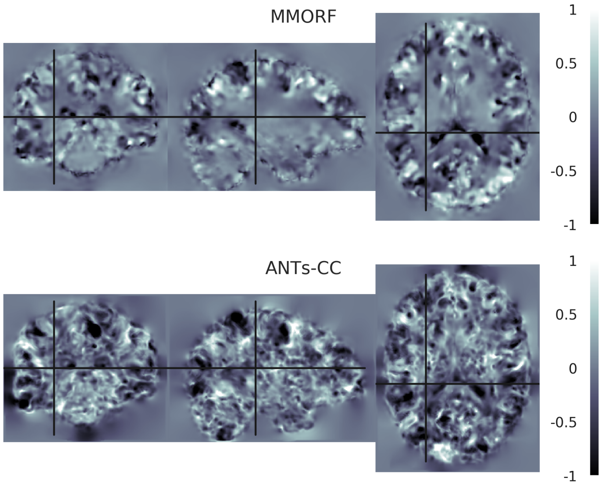

Log-Jacobian determinant spatial maps - subject 14 to 03

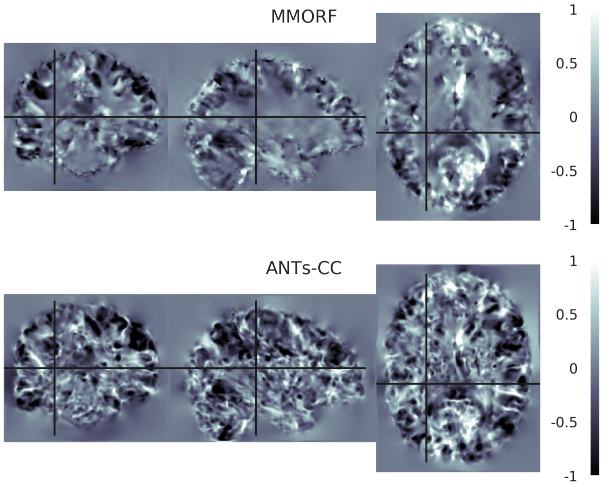

Log-Jacobian determinant spatial maps - subject 15 to 03

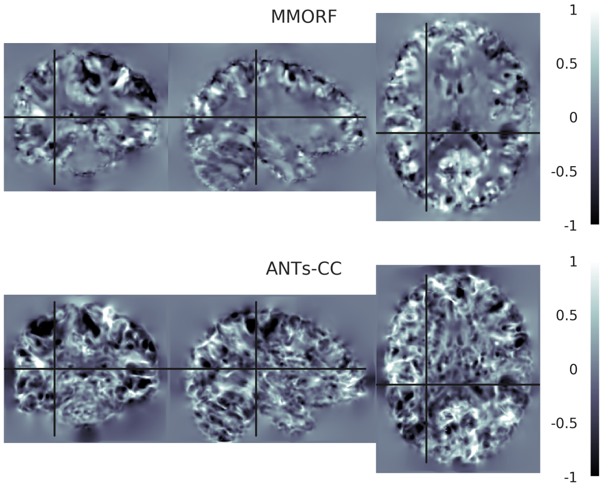

Log-Jacobian determinant spatial maps - subject 16 to 03

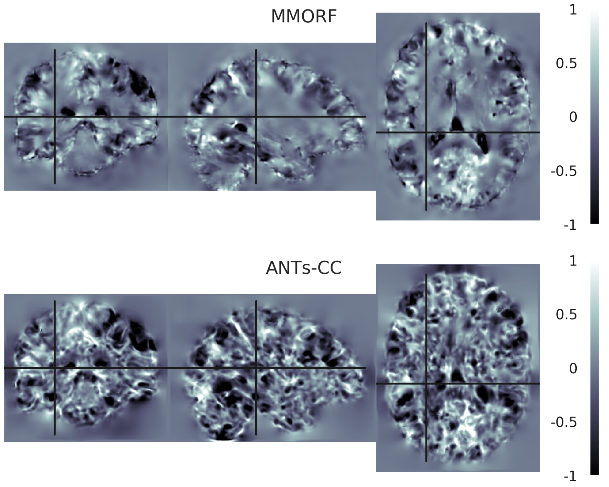

4. Reference Subject 04

Log-Jacobian determinant spatial maps - subject 01 to 04

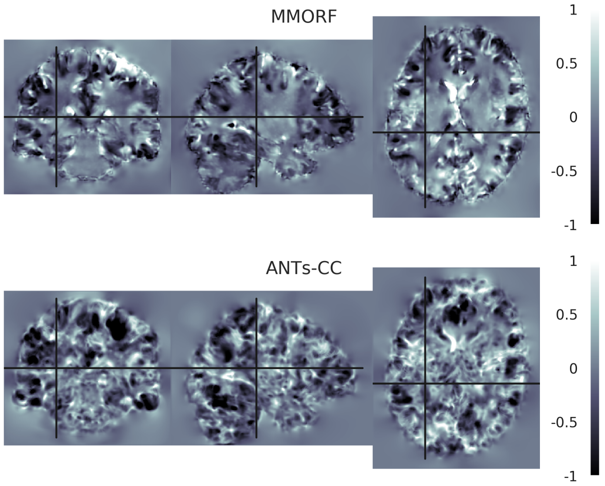

Log-Jacobian determinant spatial maps - subject 02 to 04

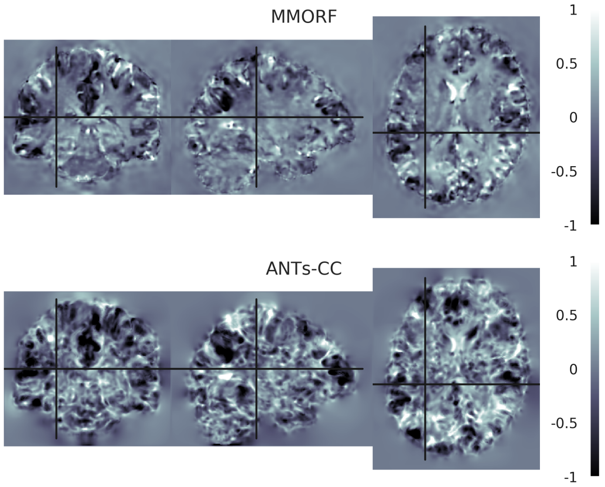

Log-Jacobian determinant spatial maps - subject 03 to 04

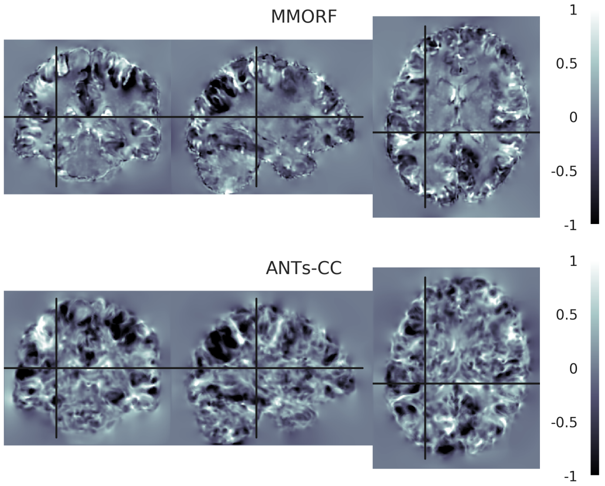

Log-Jacobian determinant spatial maps - subject 05 to 04

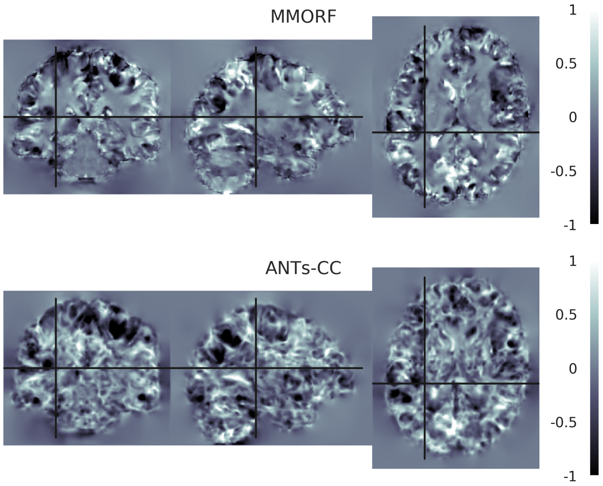

Log-Jacobian determinant spatial maps - subject 06 to 04

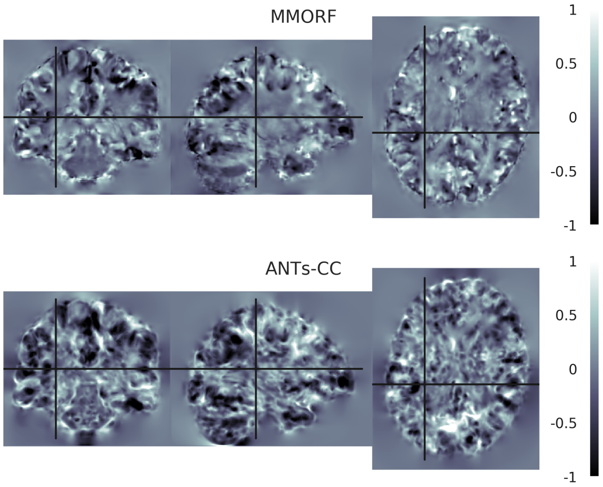

Log-Jacobian determinant spatial maps - subject 07 to 04

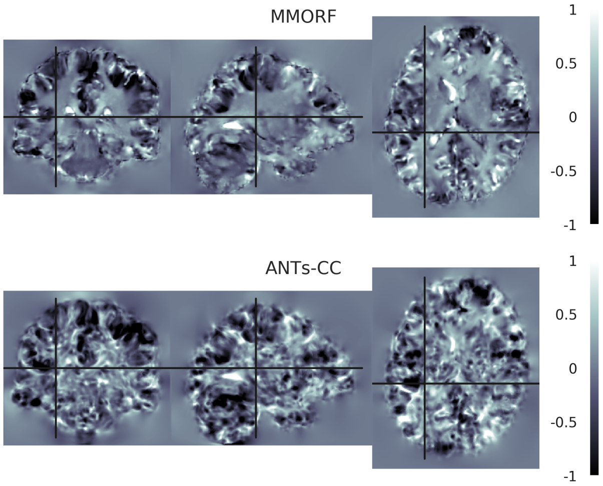

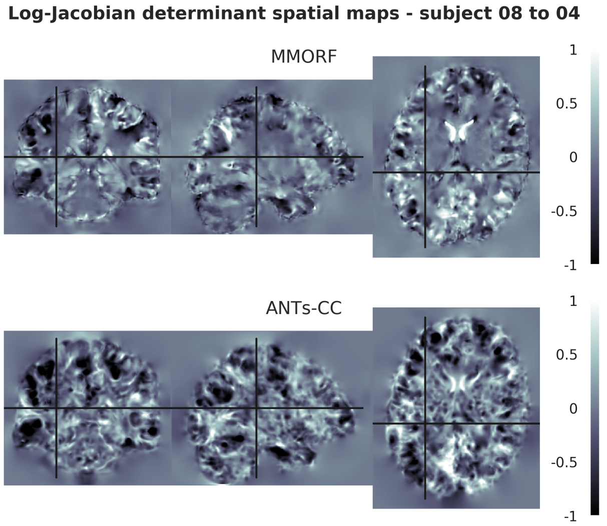
Log-Jacobian determinant spatial maps - subject 08 to 04
MMORF
ANTs-CC
1
0.5
0
-0.5
-1

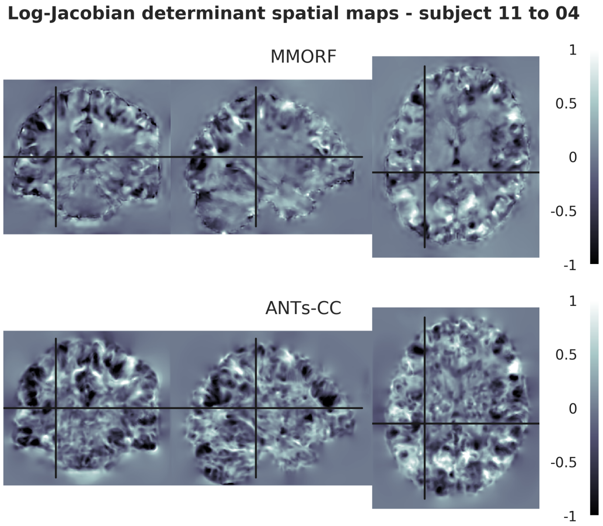
Log-Jacobian determinant spatial maps - subject 11 to 04
MMORF
ANTs-CC
1
0.5
0
-0.5
-1

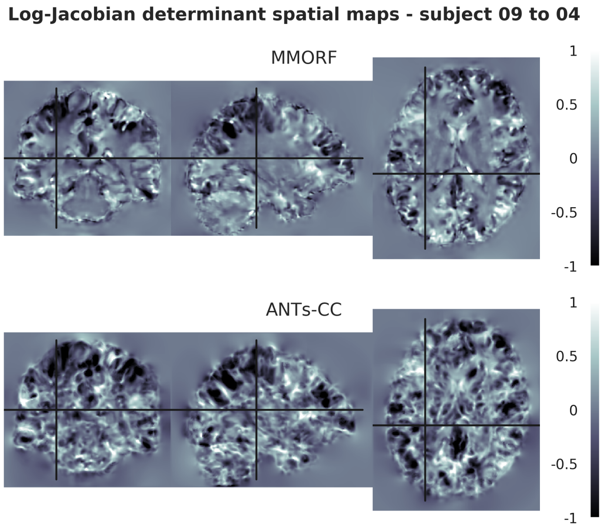
Log-Jacobian determinant spatial maps - subject 09 to 04
MMORF
ANTs-CC
1
0.5
0
-0.5
-1

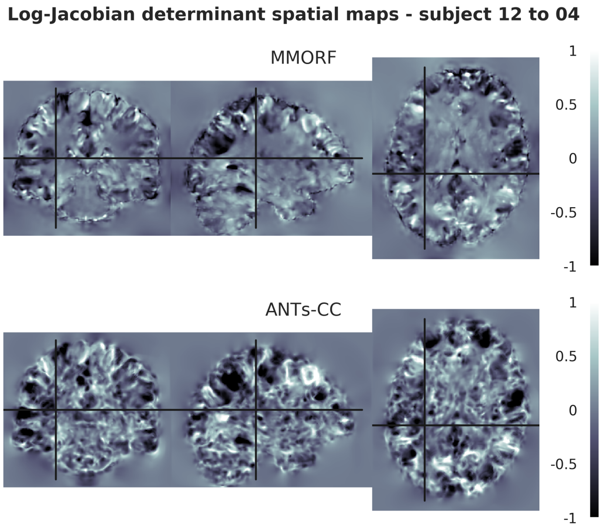
Log-Jacobian determinant spatial maps - subject 12 to 04
MMORF
ANTs-CC
1
0.5
0
-0.5
-1

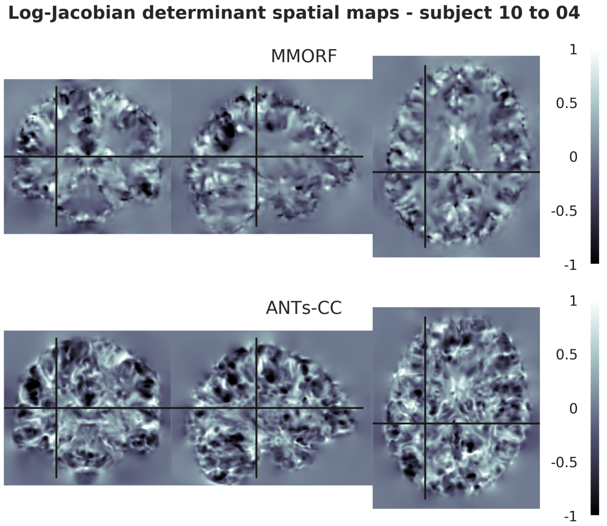
Log-Jacobian determinant spatial maps - subject 10 to 04
MMORF
ANTs-CC
1
0.5
0
-0.5
-1

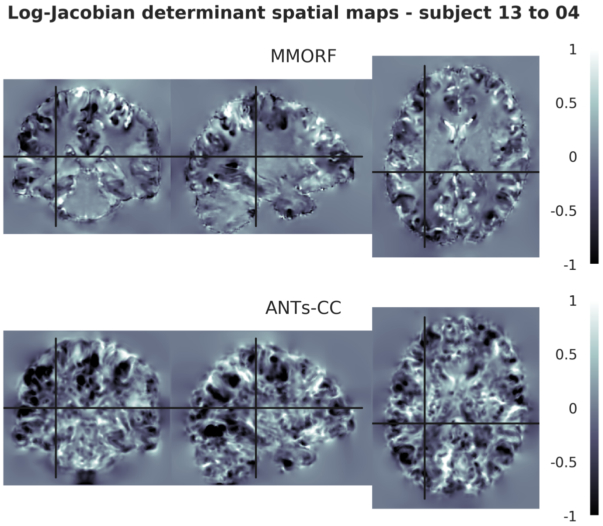
Log-Jacobian determinant spatial maps - subject 13 to 04
MMORF
ANTs-CC
1
0.5
0
-0.5
-1

Log-Jacobian determinant spatial maps - subject 14 to 04

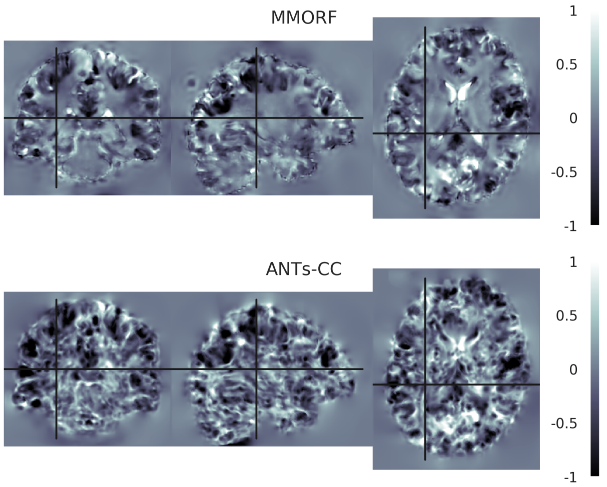

Log-Jacobian determinant spatial maps - subject 15 to 04

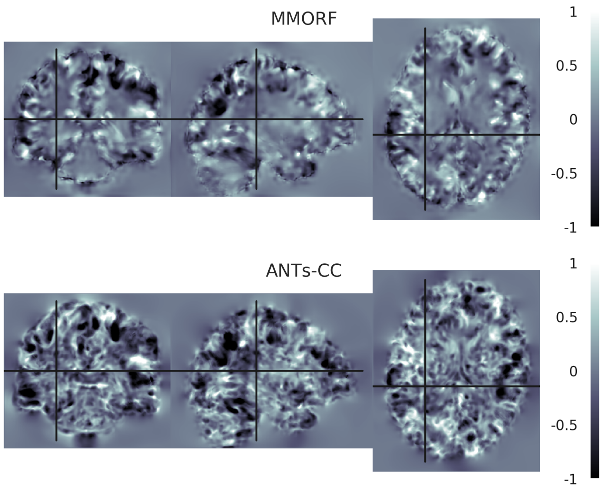

Log-Jacobian determinant spatial maps - subject 16 to 04

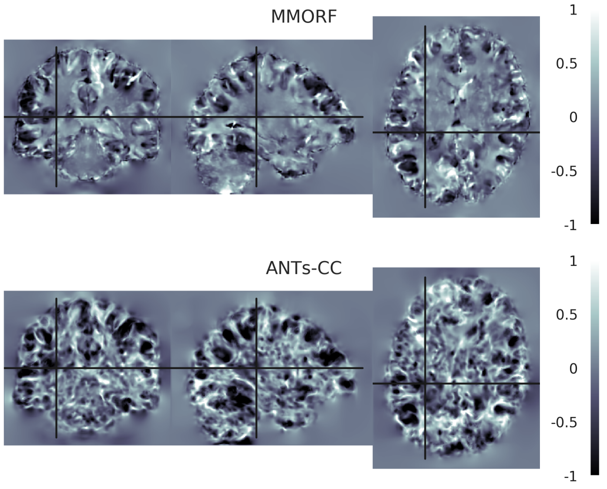

5. Reference Subject 05

Log-Jacobian determinant spatial maps - subject 01 to 05

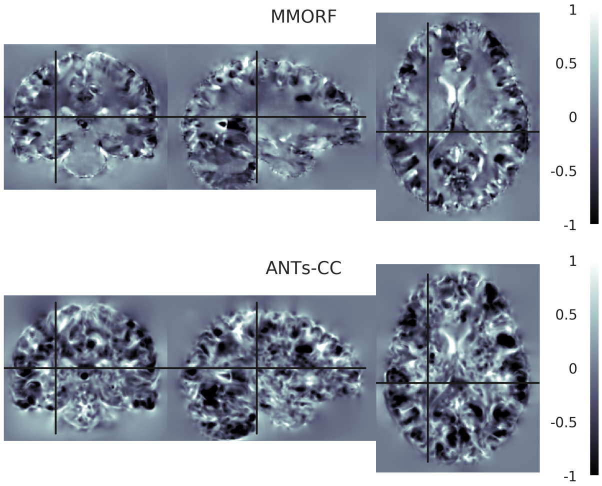

Log-Jacobian determinant spatial maps - subject 02 to 05

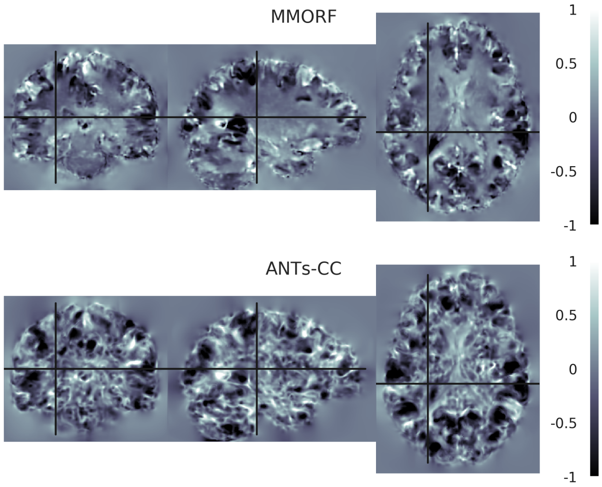

Log-Jacobian determinant spatial maps - subject 03 to 05

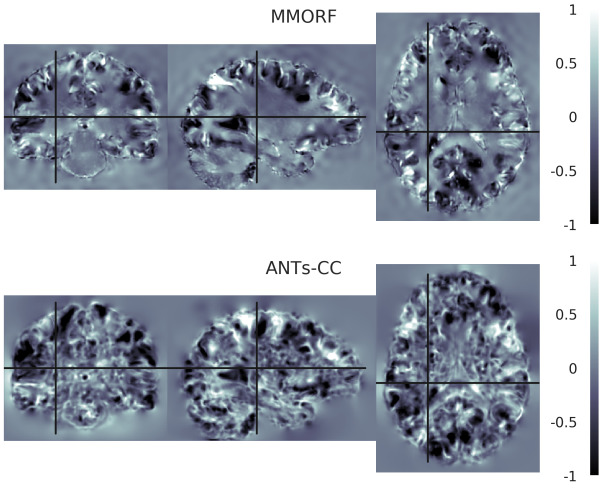

Log-Jacobian determinant spatial maps - subject 04 to 05

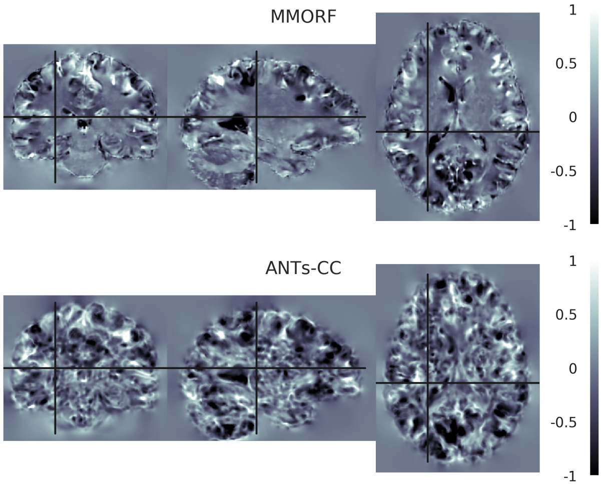

Log-Jacobian determinant spatial maps - subject 06 to 05

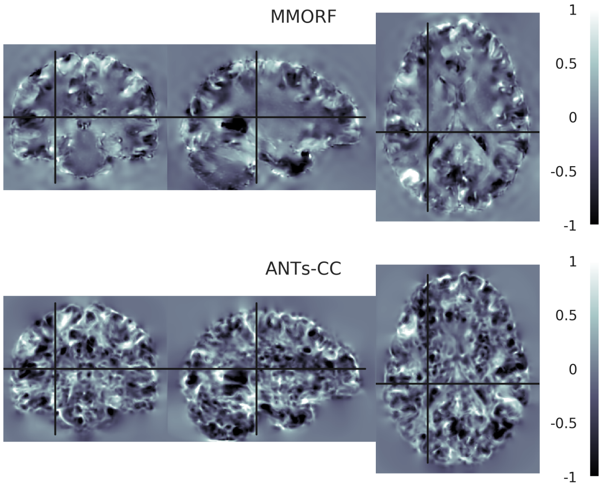

Log-Jacobian determinant spatial maps - subject 07 to 05

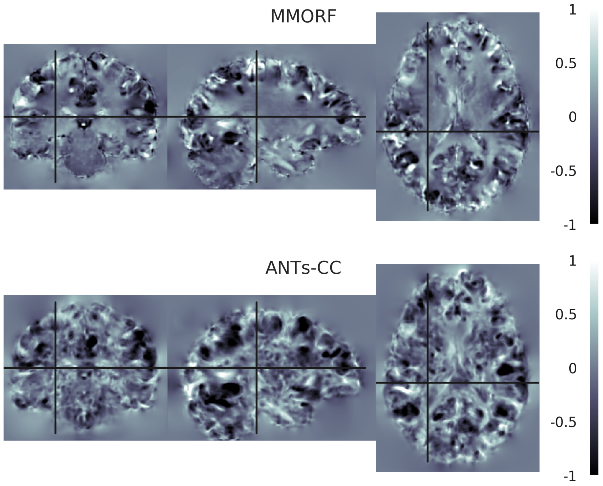

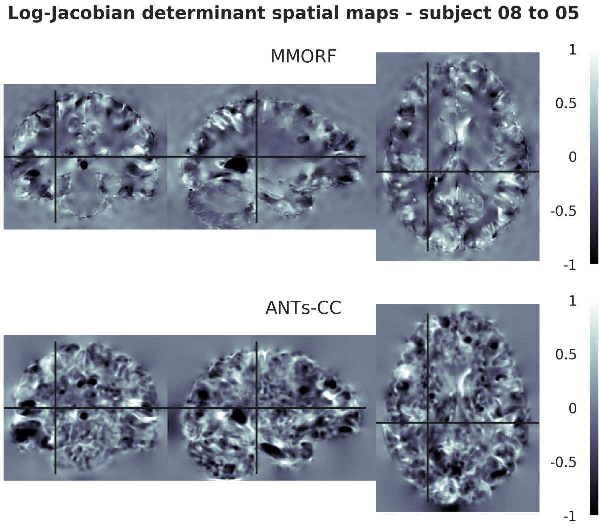
Log-Jacobian determinant spatial maps - subject 08 to 05
MMORF
ANTs-CC
1
0.5
0
-0.5
-1

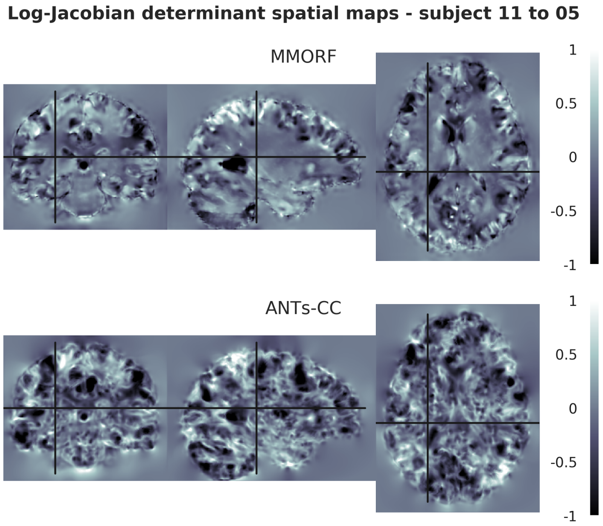
Log-Jacobian determinant spatial maps - subject 11 to 05
MMORF
ANTs-CC
1
0.5
0
-0.5
-1

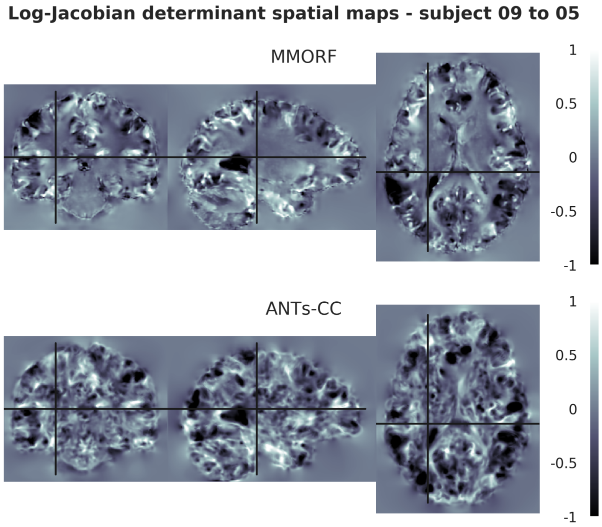
Log-Jacobian determinant spatial maps - subject 09 to 05
MMORF
ANTs-CC
1
0.5
0
-0.5
-1

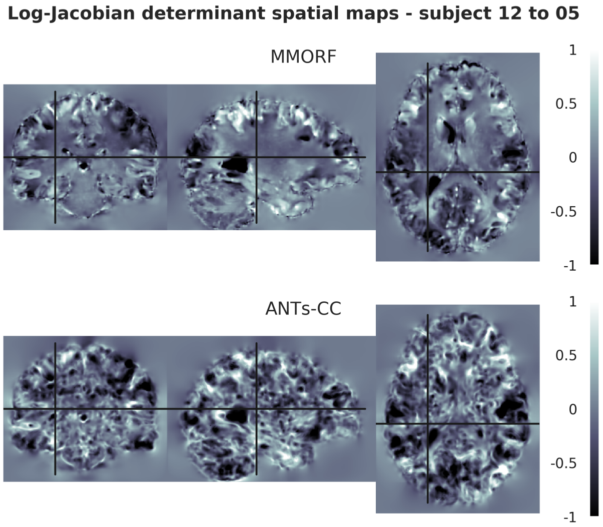
Log-Jacobian determinant spatial maps - subject 12 to 05
MMORF
ANTs-CC
1
0.5
0
-0.5
-1

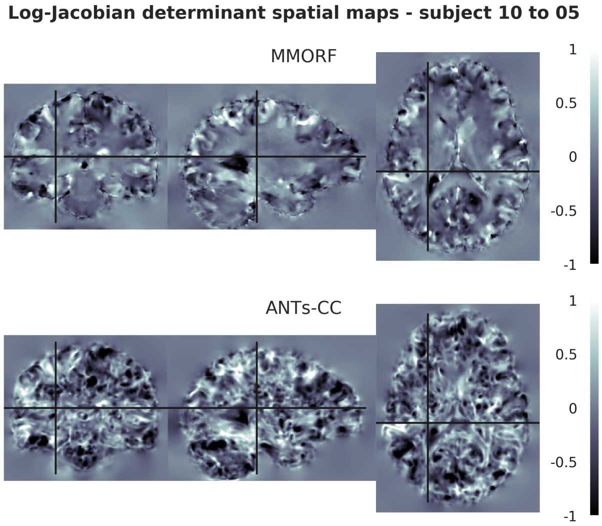
Log-Jacobian determinant spatial maps - subject 10 to 05
MMORF
ANTs-CC
1
0.5
0
-0.5
-1

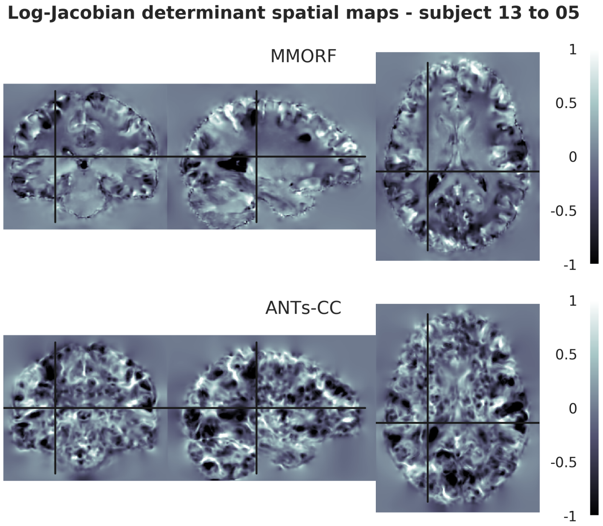
Log-Jacobian determinant spatial maps - subject 13 to 05
MMORF
ANTs-CC
1
0.5
0
-0.5
-1

Log-Jacobian determinant spatial maps - subject 14 to 05

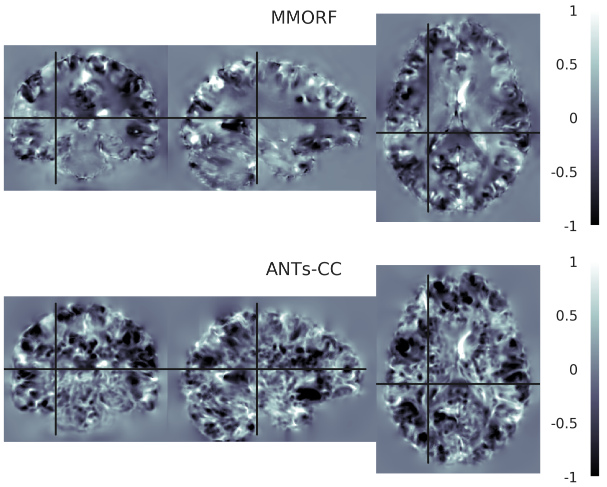

Log-Jacobian determinant spatial maps - subject 15 to 05

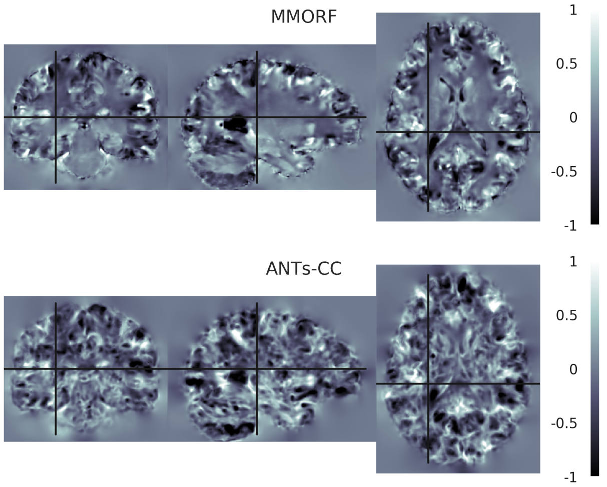

Log-Jacobian determinant spatial maps - subject 16 to 05

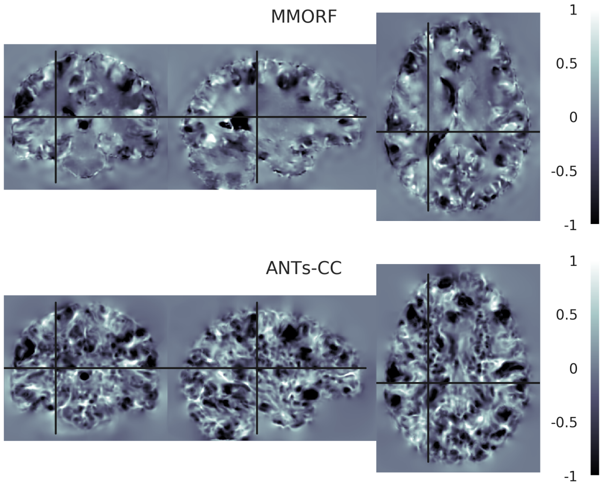

6. Reference Subject 06

Log-Jacobian determinant spatial maps - subject 01 to 06

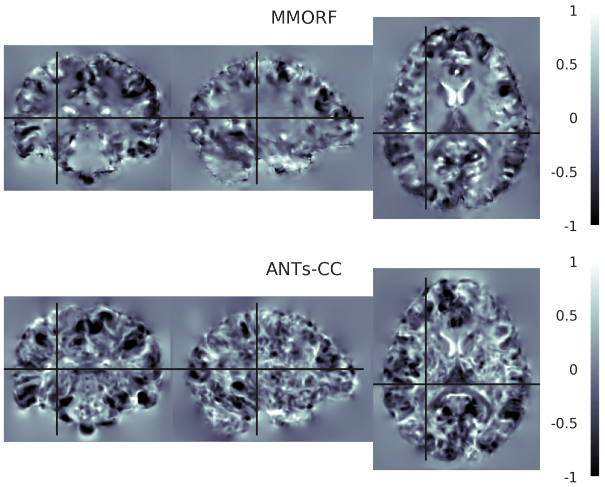

Log-Jacobian determinant spatial maps - subject 02 to 06

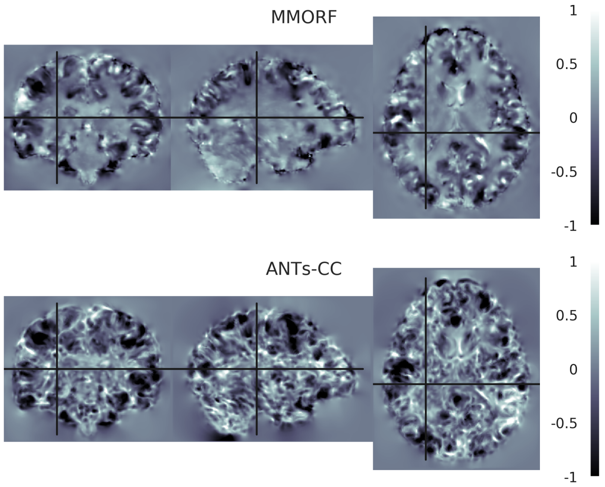

Log-Jacobian determinant spatial maps - subject 03 to 06

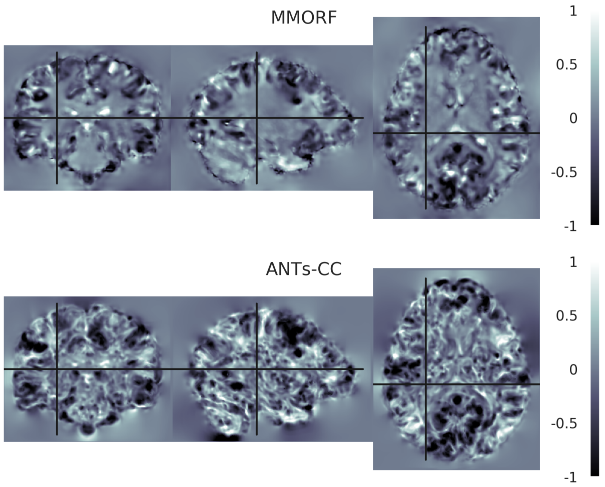

Log-Jacobian determinant spatial maps - subject 04 to 06

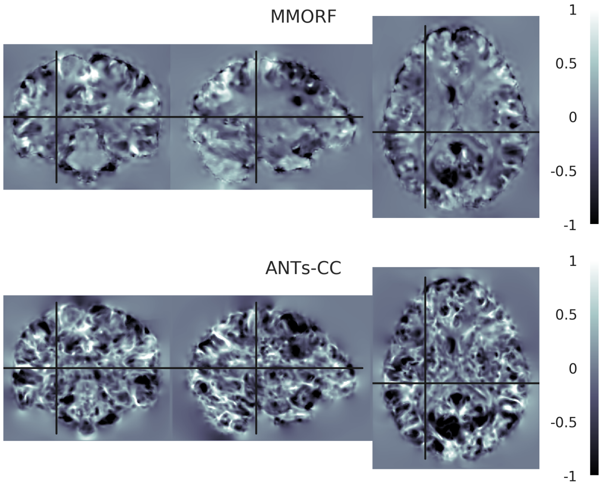

Log-Jacobian determinant spatial maps - subject 05 to 06

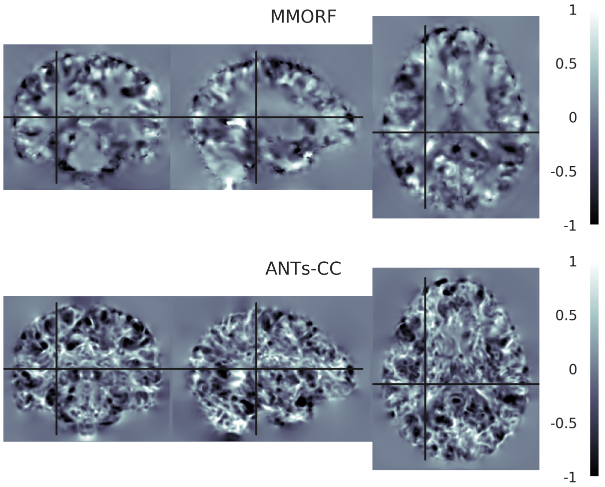

Log-Jacobian determinant spatial maps - subject 07 to 06

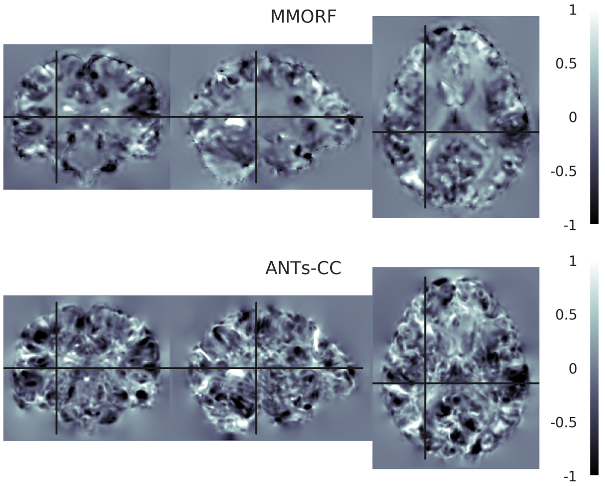

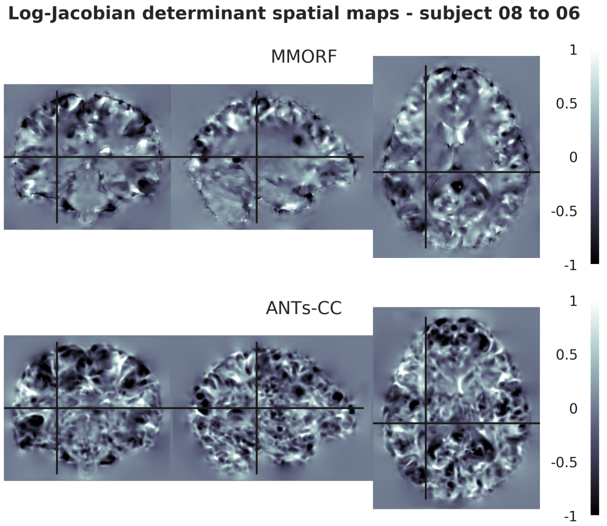
Log-Jacobian determinant spatial maps - subject 08 to 06
MMORF
ANTs-CC
1
0.5
0
-0.5
-1

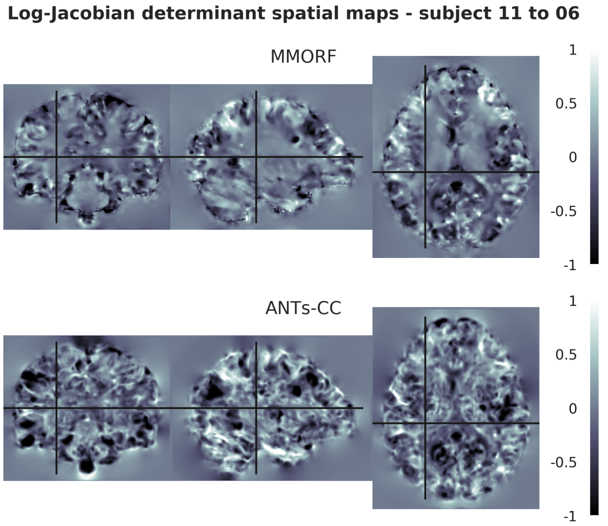
Log-Jacobian determinant spatial maps - subject 11 to 06
MMORF
ANTs-CC
1
0.5
0
-0.5
-1

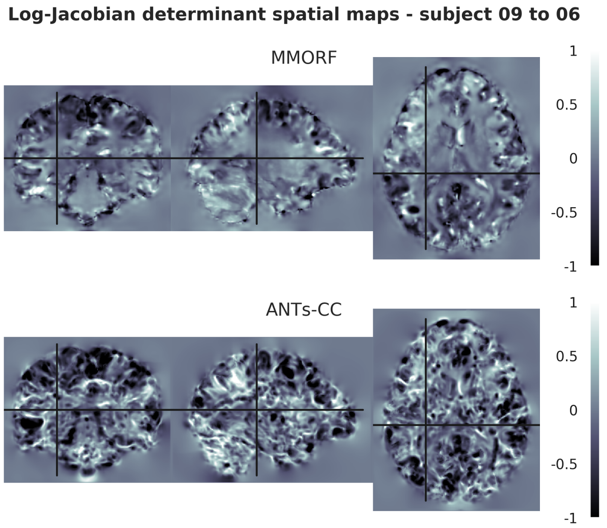
Log-Jacobian determinant spatial maps - subject 09 to 06
MMORF
ANTs-CC
1
0.5
0
-0.5
-1

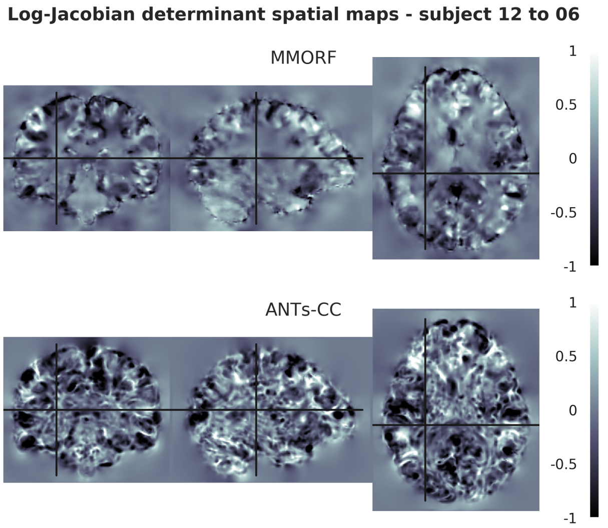
Log-Jacobian determinant spatial maps - subject 12 to 06
MMORF
ANTs-CC
1
0.5
0
-0.5
-1

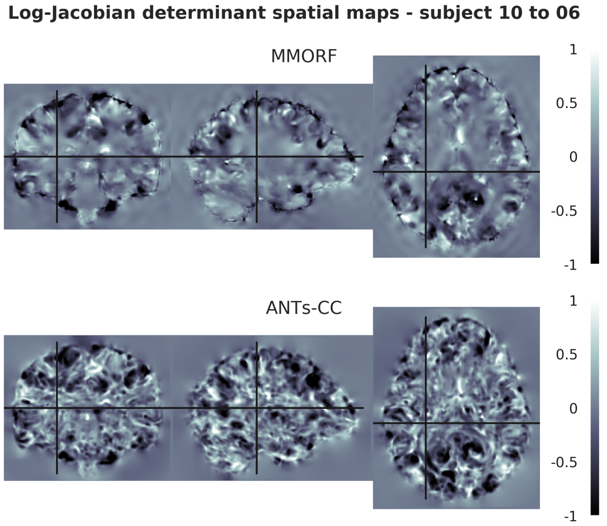
Log-Jacobian determinant spatial maps - subject 10 to 06
MMORF
ANTs-CC
1
0.5
0
-0.5
-1

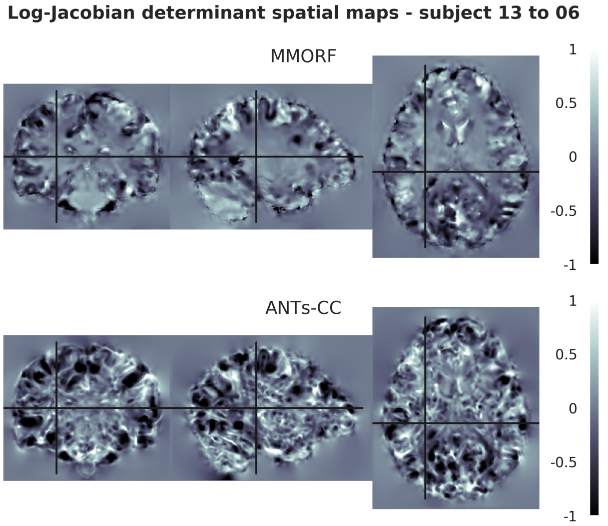
Log-Jacobian determinant spatial maps - subject 13 to 06
MMORF
ANTs-CC
1
0.5
0
-0.5
-1

Log-Jacobian determinant spatial maps - subject 14 to 06

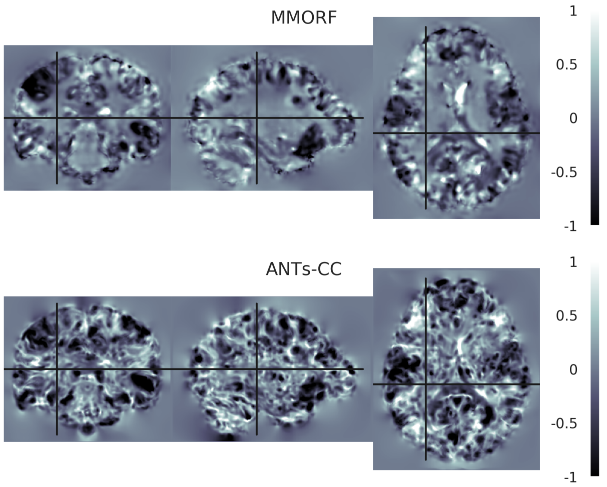

Log-Jacobian determinant spatial maps - subject 15 to 06

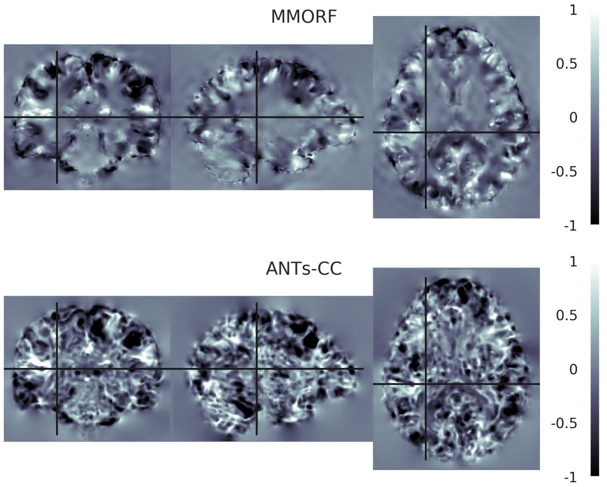

Log-Jacobian determinant spatial maps - subject 16 to 06

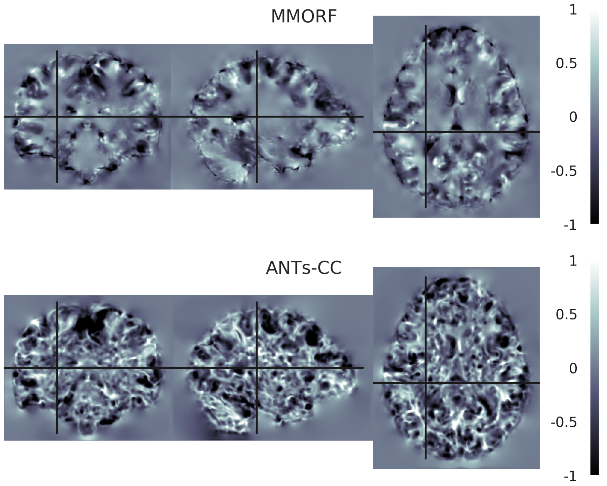

7. Reference Subject 07

Log-Jacobian determinant spatial maps - subject 01 to 07

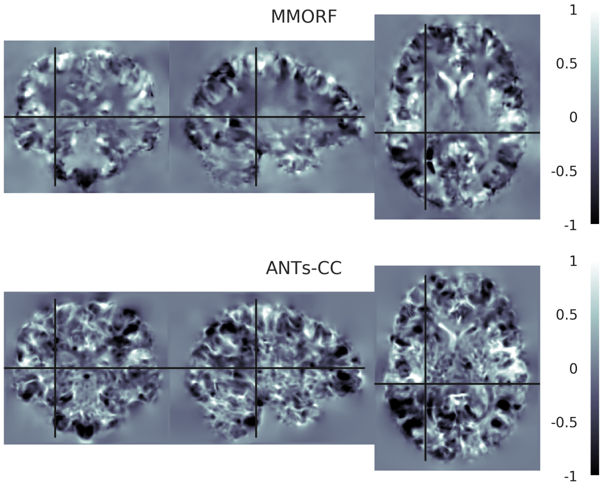

Log-Jacobian determinant spatial maps - subject 02 to 07

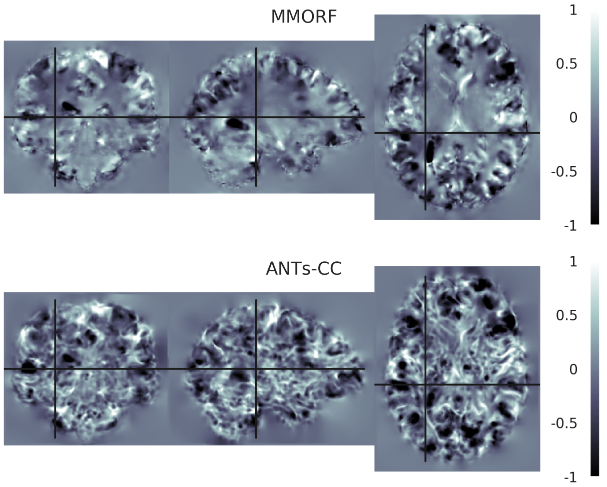

Log-Jacobian determinant spatial maps - subject 03 to 07

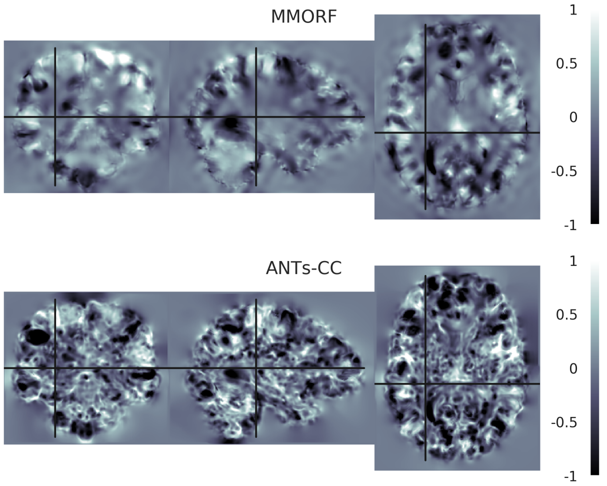

Log-Jacobian determinant spatial maps - subject 04 to 07

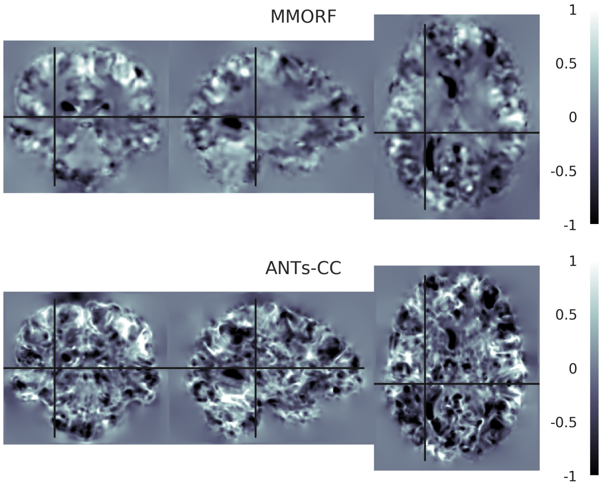

Log-Jacobian determinant spatial maps - subject 05 to 07

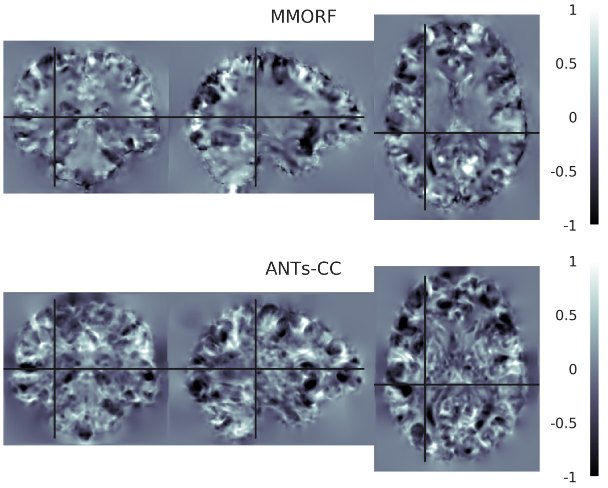

Log-Jacobian determinant spatial maps - subject 06 to 07

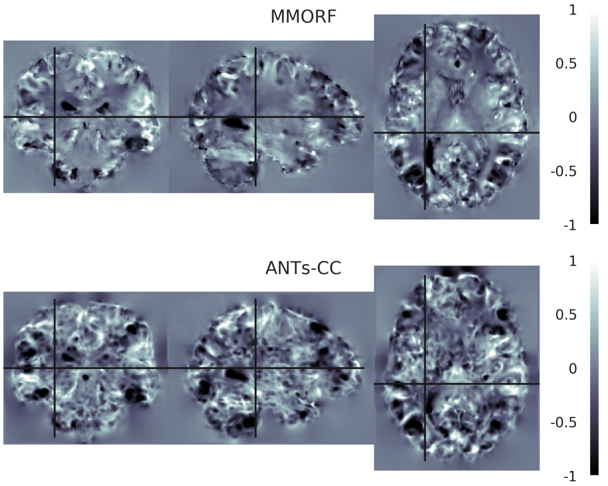

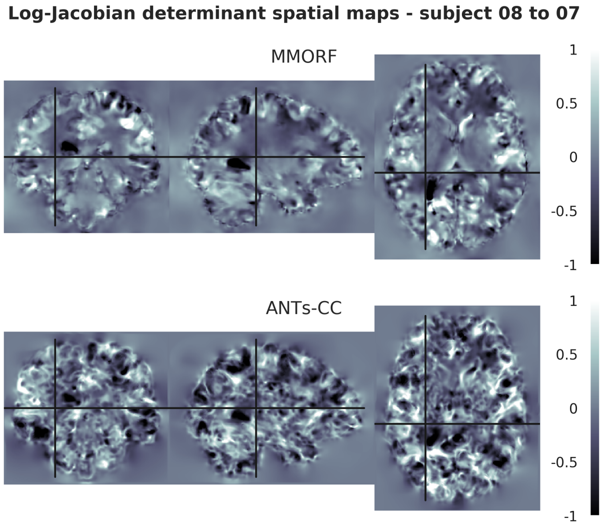
Log-Jacobian determinant spatial maps - subject 08 to 07
MMORF
ANTs-CC
1
0.5
0
-0.5
-1

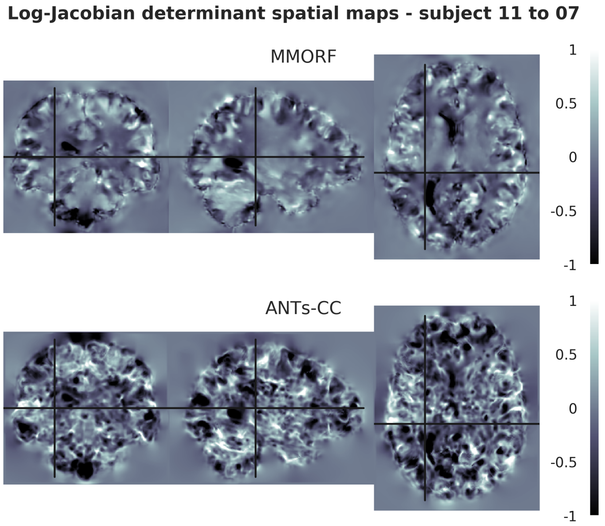
Log-Jacobian determinant spatial maps - subject 11 to 07
MMORF
ANTs-CC
1
0.5
0
-0.5
-1

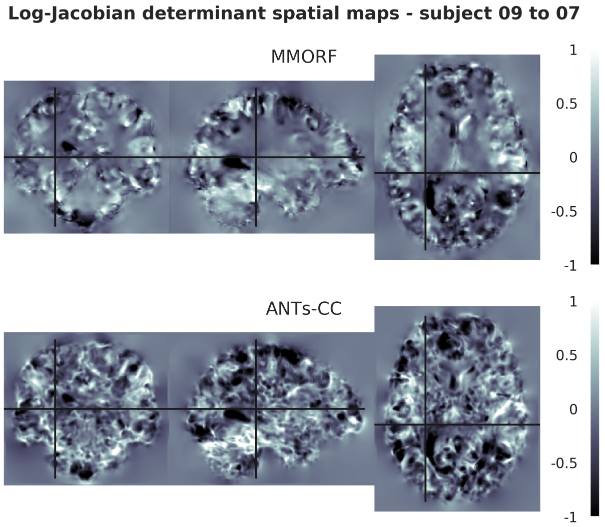
Log-Jacobian determinant spatial maps - subject 09 to 07
MMORF
ANTs-CC
1
0.5
0
-0.5
-1

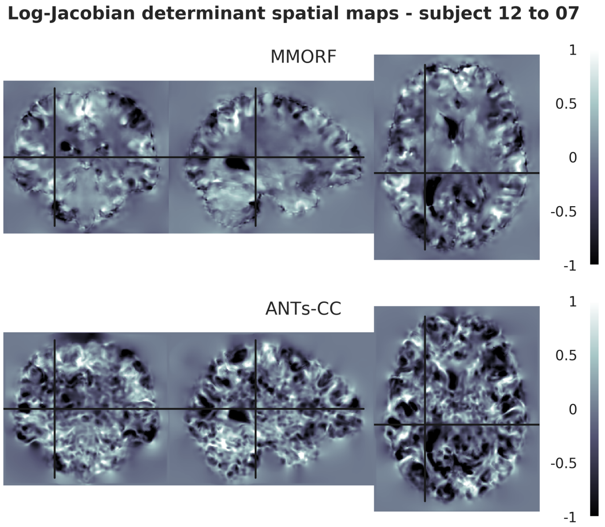
Log-Jacobian determinant spatial maps - subject 12 to 07
MMORF
ANTs-CC
1
0.5
0
-0.5
-1

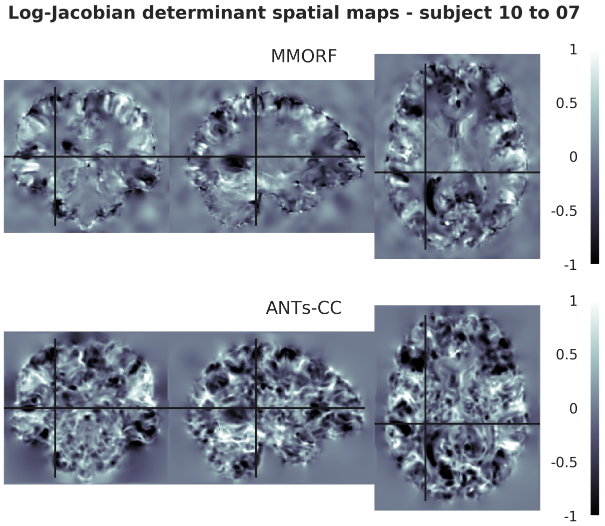
Log-Jacobian determinant spatial maps - subject 10 to 07
MMORF
ANTs-CC
1
0.5
0
-0.5
-1

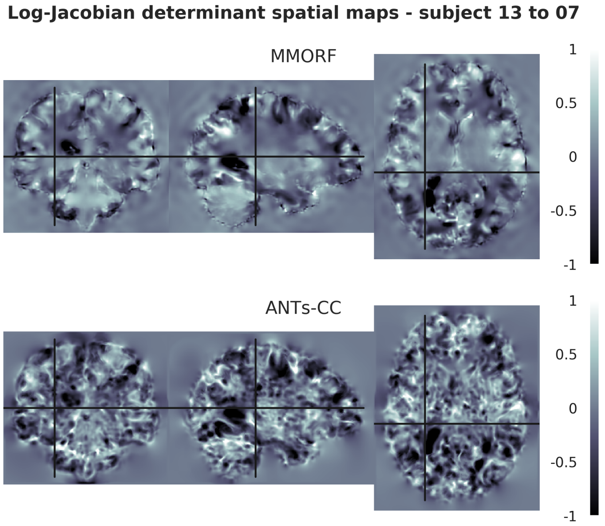
Log-Jacobian determinant spatial maps - subject 13 to 07
MMORF
ANTs-CC
1
0.5
0
-0.5
-1

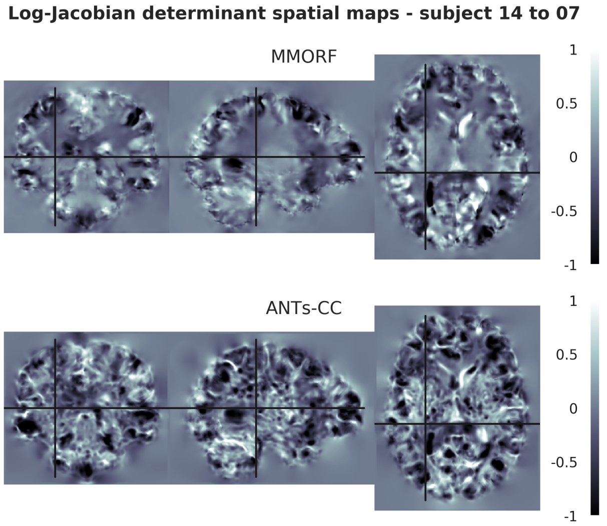
Log-Jacobian determinant spatial maps - subject 14 to 07
MMORF
ANTs-CC
1
0.5
0
-0.5
-1

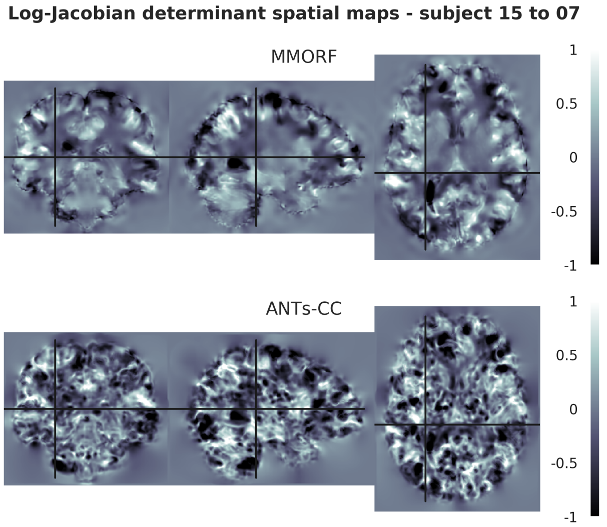
Log-Jacobian determinant spatial maps - subject 15 to 07
MMORF
ANTs-CC
1
0.5
0
-0.5
-1

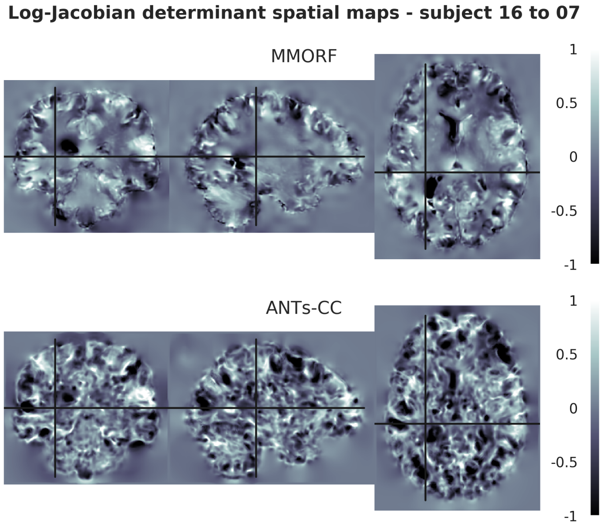
Log-Jacobian determinant spatial maps - subject 16 to 07
MMORF
ANTs-CC
1
0.5
0
-0.5
-1

8. Reference Subject 08

Log-Jacobian determinant spatial maps - subject 01 to 08

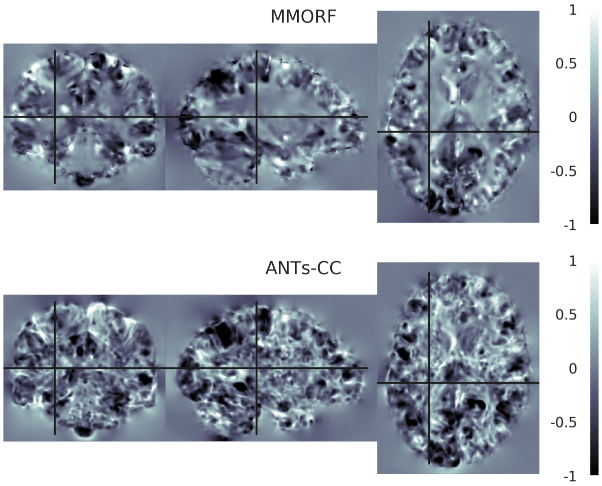

Log-Jacobian determinant spatial maps - subject 02 to 08

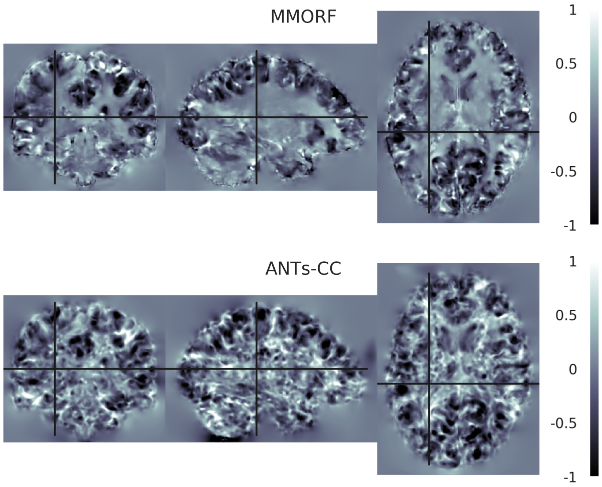

Log-Jacobian determinant spatial maps - subject 03 to 08

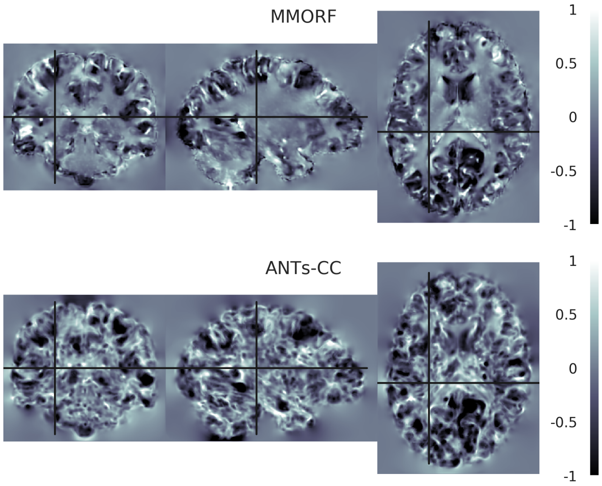

Log-Jacobian determinant spatial maps - subject 04 to 08

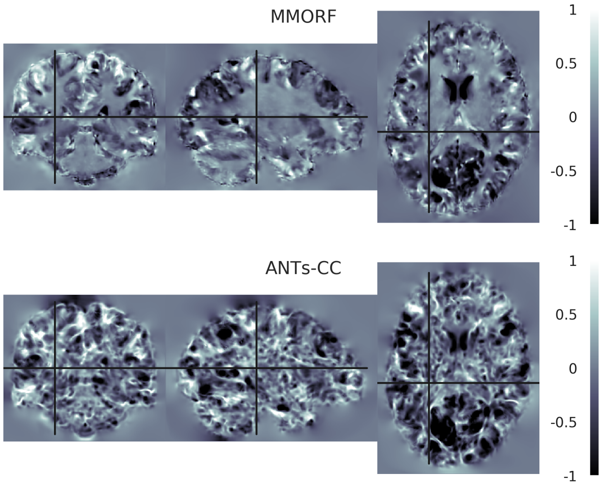

Log-Jacobian determinant spatial maps - subject 05 to 08

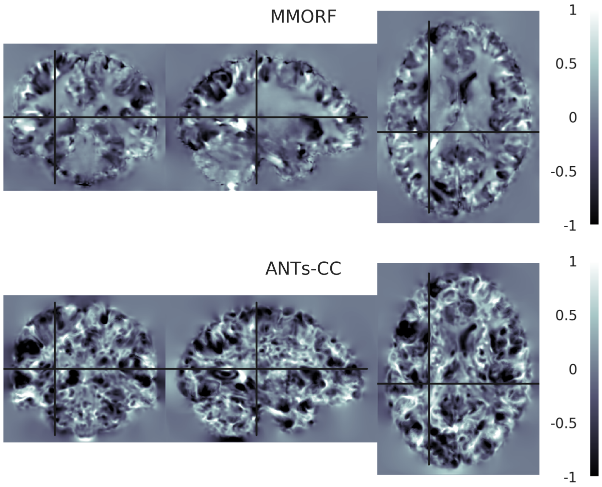

Log-Jacobian determinant spatial maps - subject 06 to 08

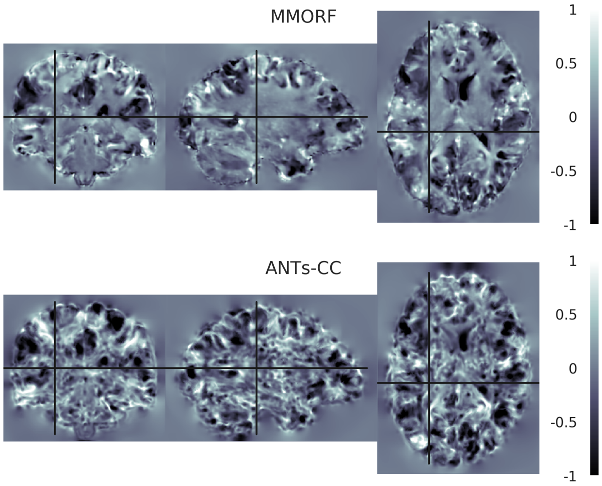

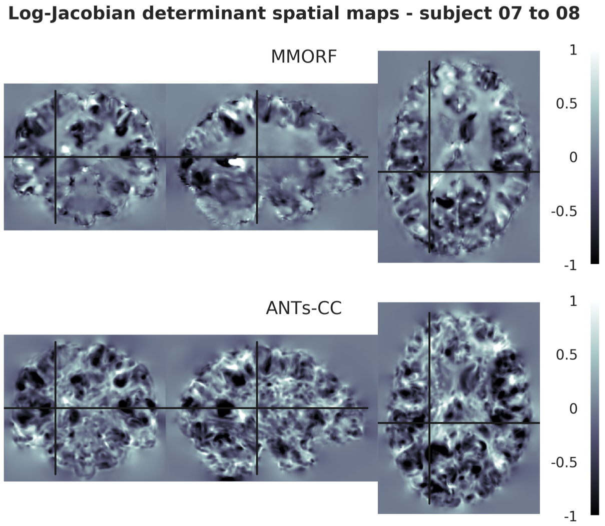
Log-Jacobian determinant spatial maps - subject 07 to 08
MMORF
1
0.5
0
-0.5
-1
ANTs-CC
1
0.5
0
-0.5
-1

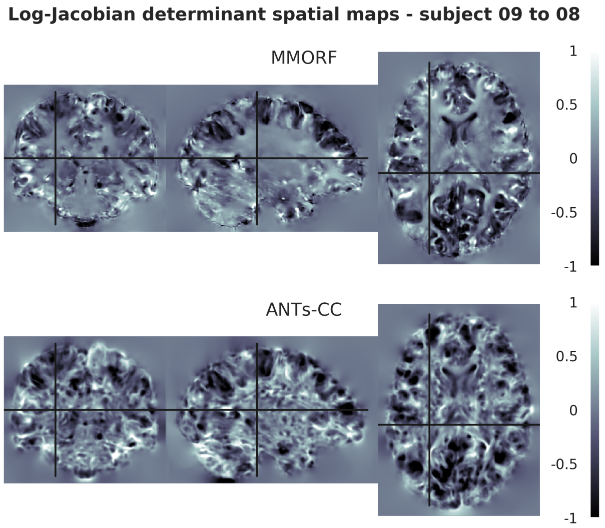
Log-Jacobian determinant spatial maps - subject 09 to 08
MMORF
1
0.5
0
-0.5
-1
ANTs-CC
1
0.5
0
-0.5
-1

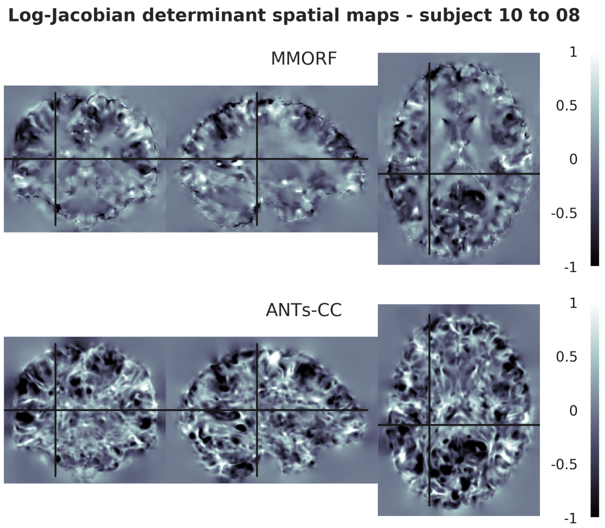
Log-Jacobian determinant spatial maps - subject 10 to 08
MMORF
1
0.5
0
-0.5
-1
ANTs-CC
1
0.5
0
-0.5
-1

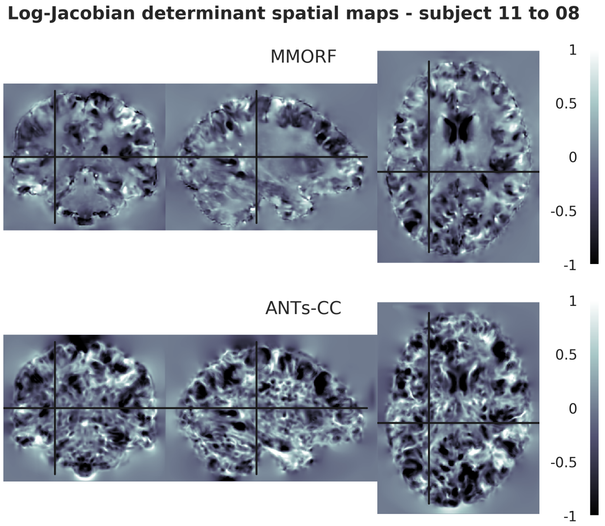
Log-Jacobian determinant spatial maps - subject 11 to 08
MMORF
1
0.5
0
-0.5
-1
ANTs-CC
1
0.5
0
-0.5
-1

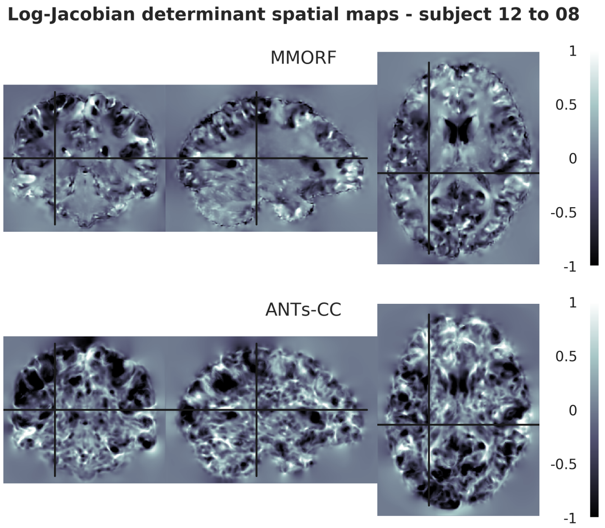
Log-Jacobian determinant spatial maps - subject 12 to 08
MMORF
1
0.5
0
-0.5
-1
ANTs-CC
1
0.5
0
-0.5
-1

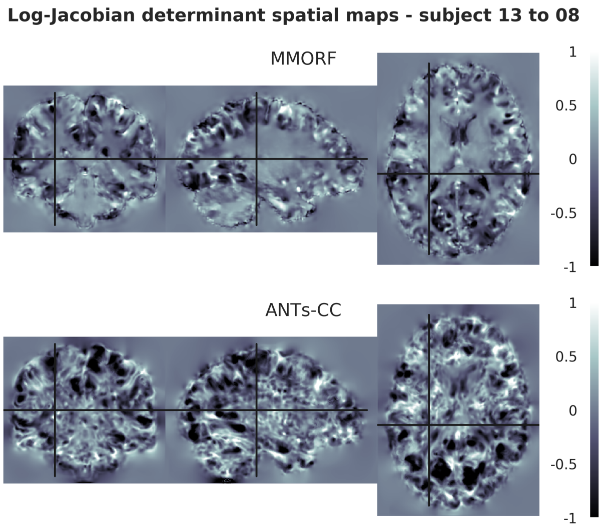
Log-Jacobian determinant spatial maps - subject 13 to 08
MMORF
1
0.5
0
-0.5
-1
ANTs-CC
1
0.5
0
-0.5
-1

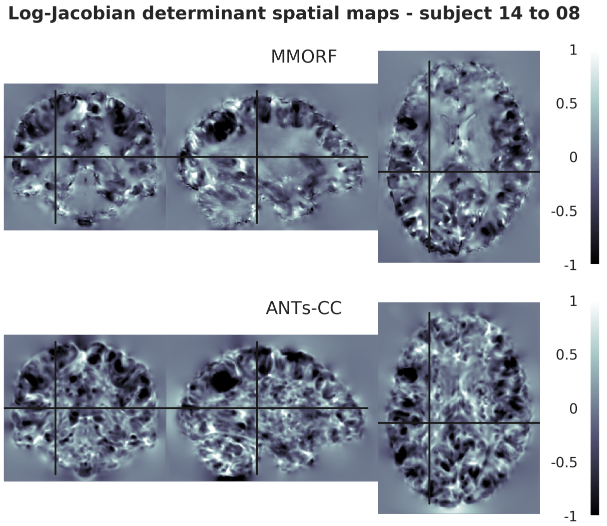
Log-Jacobian determinant spatial maps - subject 14 to 08
MMORF
ANTs-CC
1
0.5
0
-0.5
-1

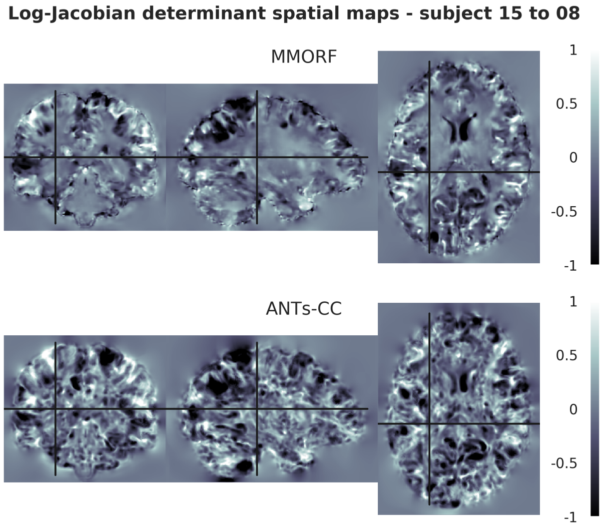
Log-Jacobian determinant spatial maps - subject 15 to 08
MMORF
ANTs-CC
1
0.5
0
-0.5
-1

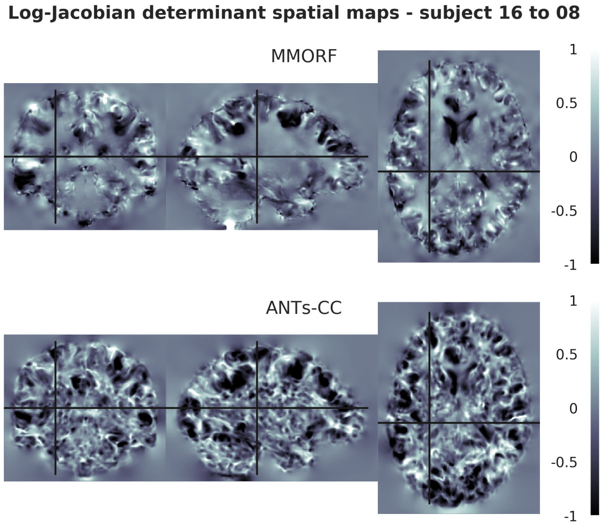
Log-Jacobian determinant spatial maps - subject 16 to 08
MMORF
ANTs-CC
1
0.5
0
-0.5
-1

9. Reference Subject 09

Log-Jacobian determinant spatial maps - subject 01 to 09

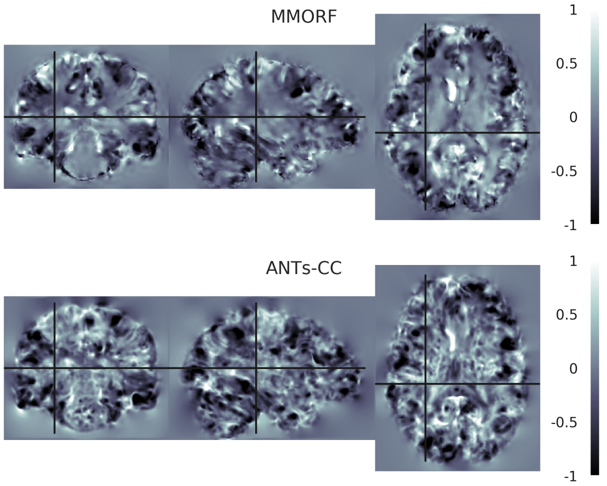

Log-Jacobian determinant spatial maps - subject 02 to 09

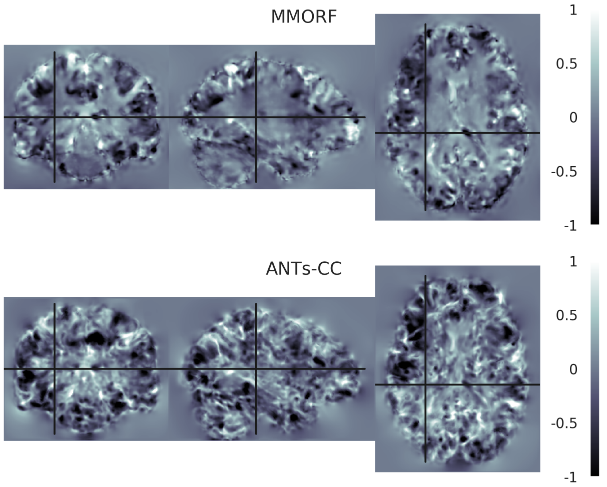

Log-Jacobian determinant spatial maps - subject 03 to 09

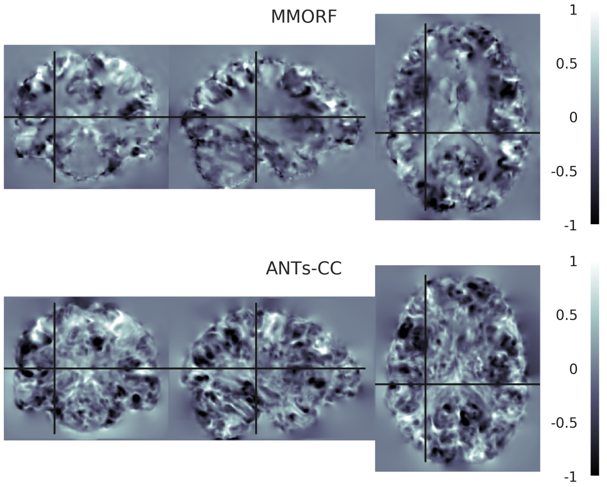

Log-Jacobian determinant spatial maps - subject 04 to 09

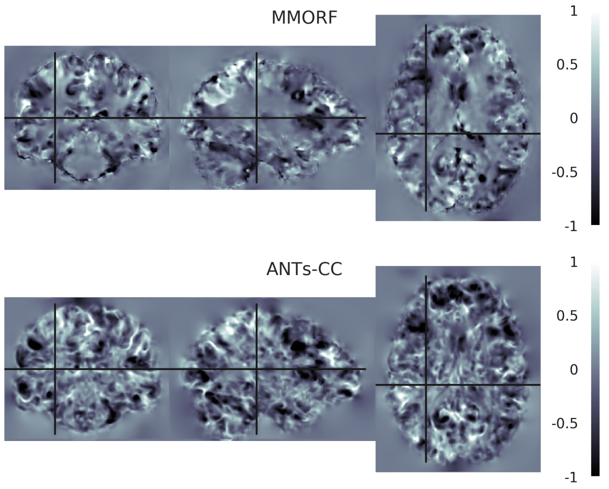

Log-Jacobian determinant spatial maps - subject 05 to 09

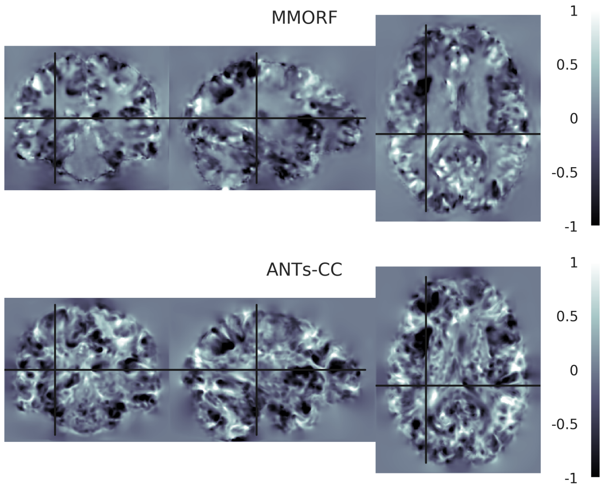

Log-Jacobian determinant spatial maps - subject 06 to 09

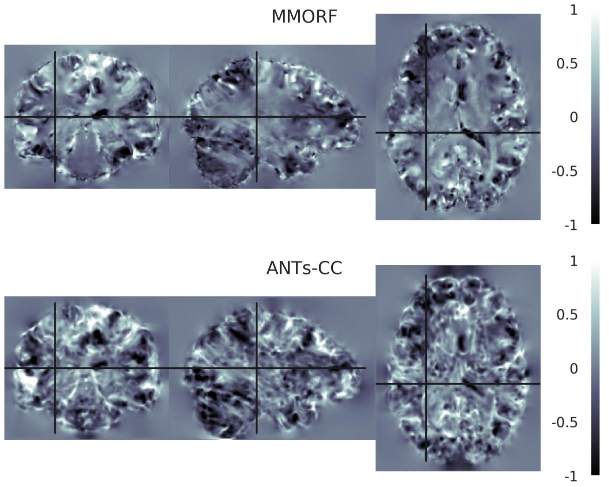

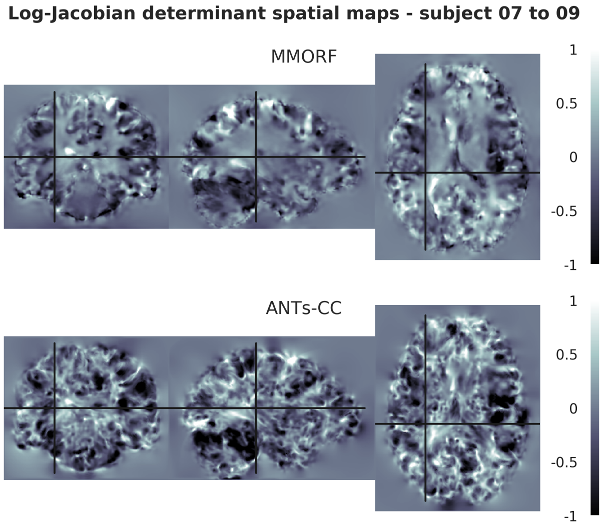
Log-Jacobian determinant spatial maps - subject 07 to 09
MMORF
ANTs-CC
1
0.5
0
-0.5
-1

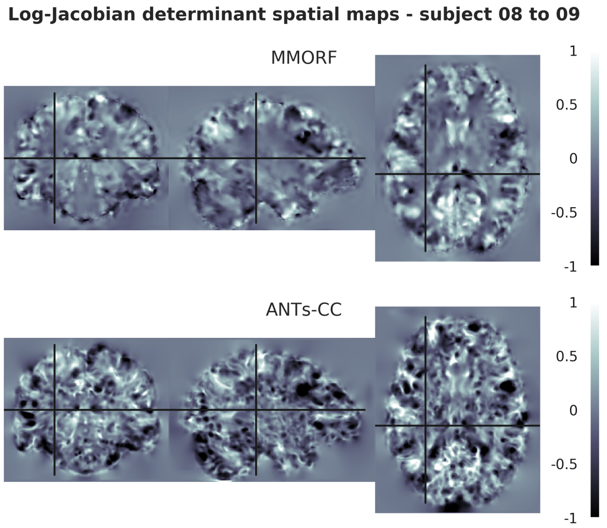
Log-Jacobian determinant spatial maps - subject 08 to 09
MMORF
ANTs-CC
1
0.5
0
-0.5
-1

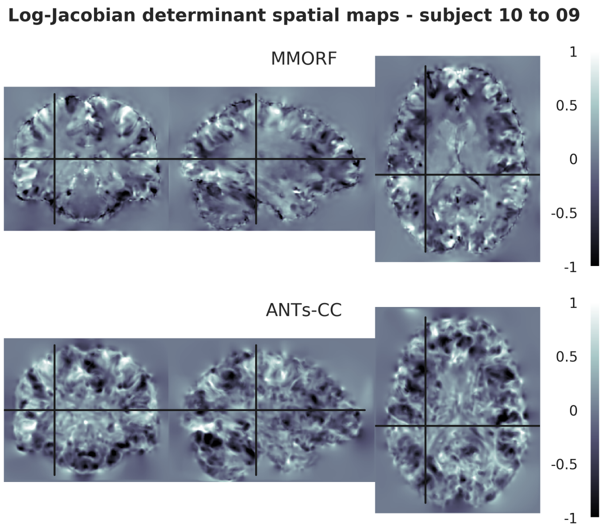
Log-Jacobian determinant spatial maps - subject 10 to 09
MMORF
ANTs-CC
1
0.5
0
-0.5
-1

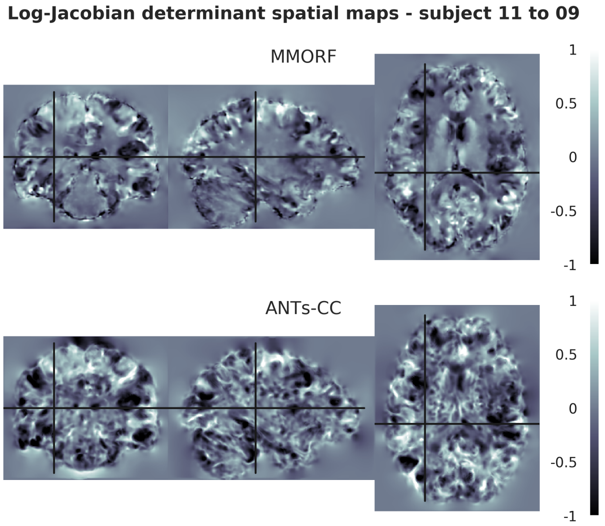
Log-Jacobian determinant spatial maps - subject 11 to 09
MMORF
ANTs-CC
1
0.5
0
-0.5
-1

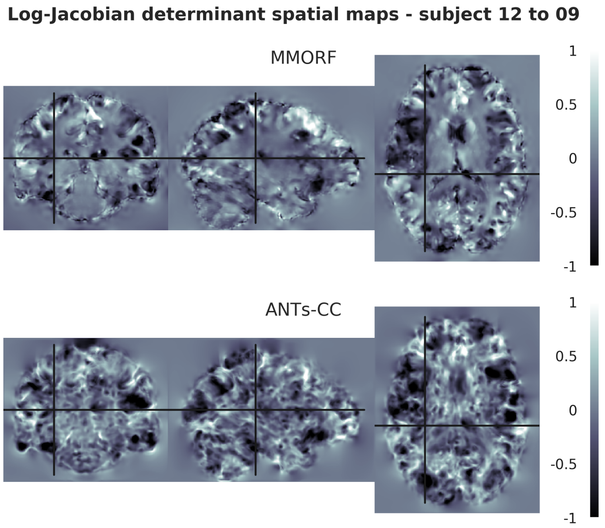
Log-Jacobian determinant spatial maps - subject 12 to 09
MMORF
ANTs-CC
1
0.5
0
-0.5
-1

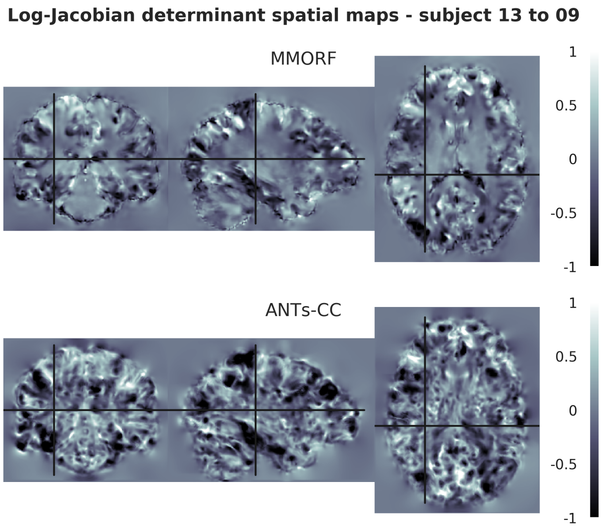
Log-Jacobian determinant spatial maps - subject 13 to 09
MMORF
ANTs-CC
1
0.5
0
-0.5
-1

Log-Jacobian determinant spatial maps - subject 14 to 09

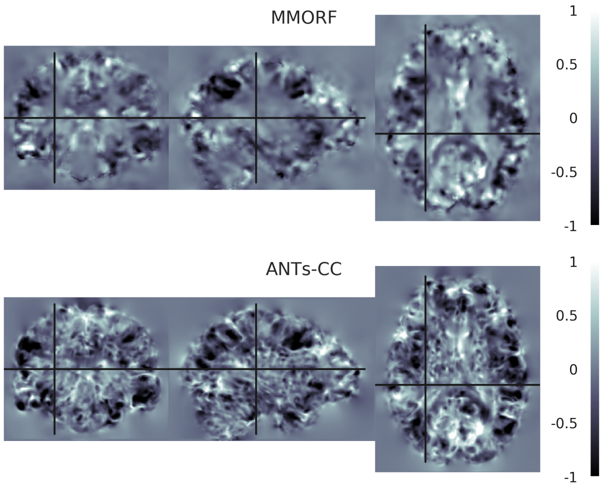

Log-Jacobian determinant spatial maps - subject 15 to 09

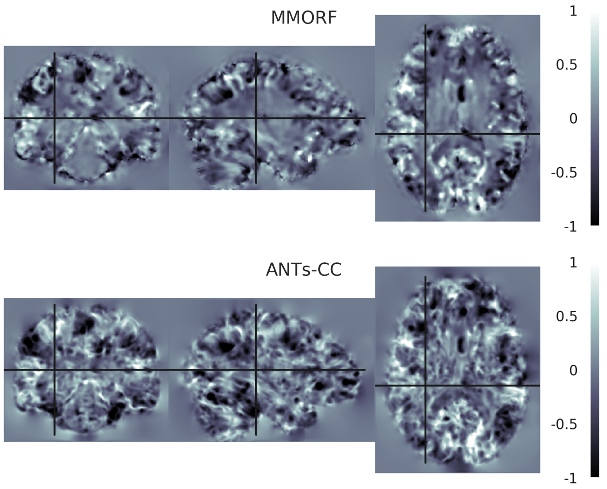

Log-Jacobian determinant spatial maps - subject 16 to 09

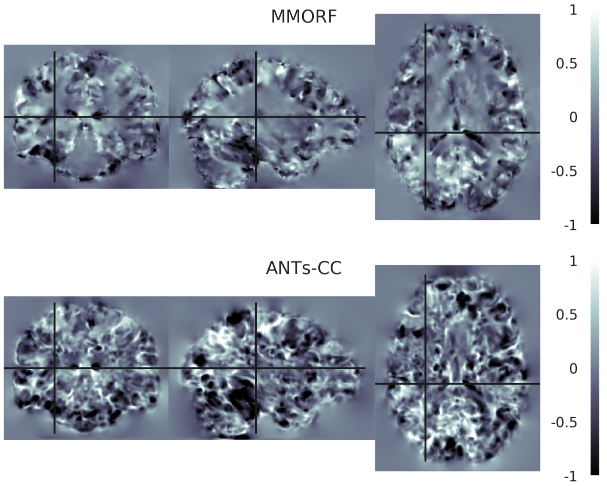

10. Reference Subject 10

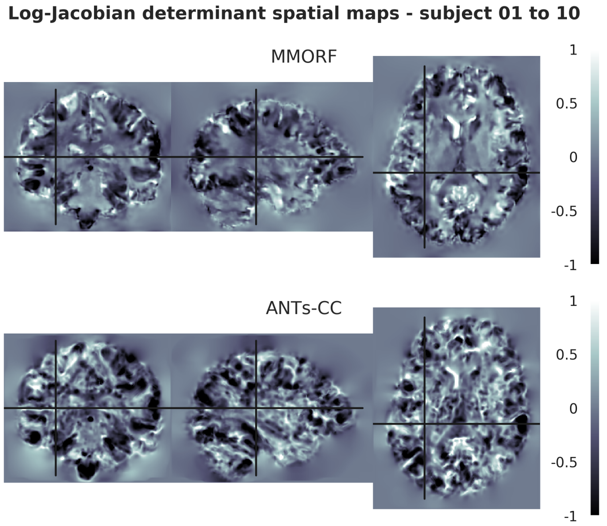

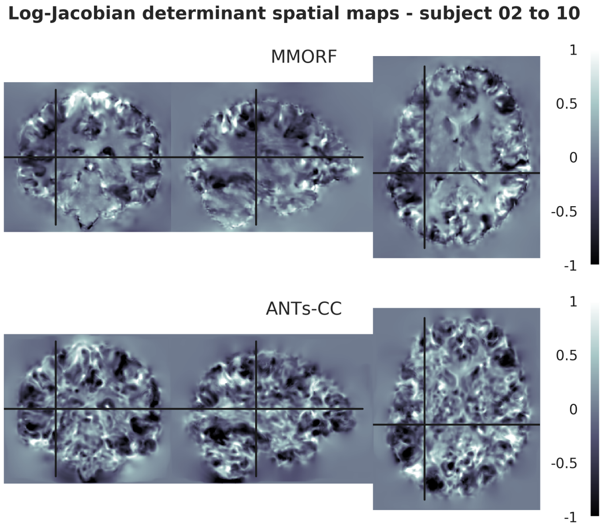

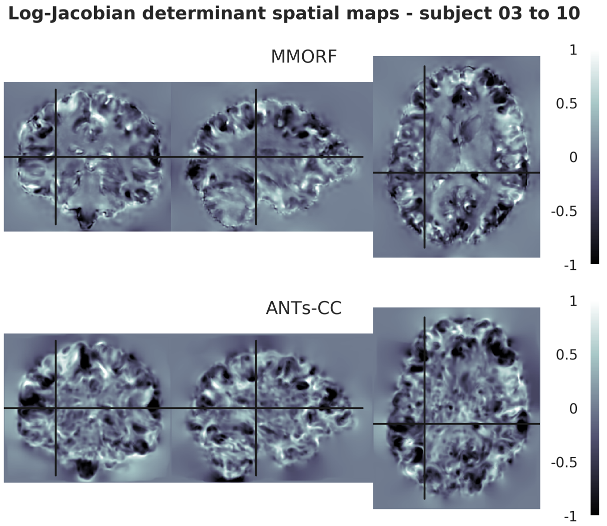

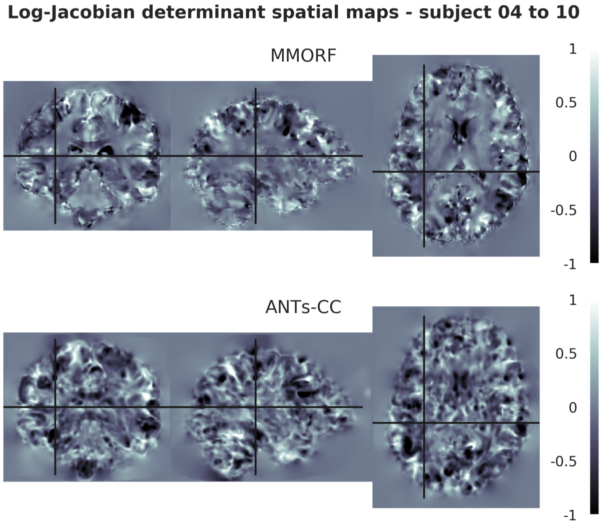

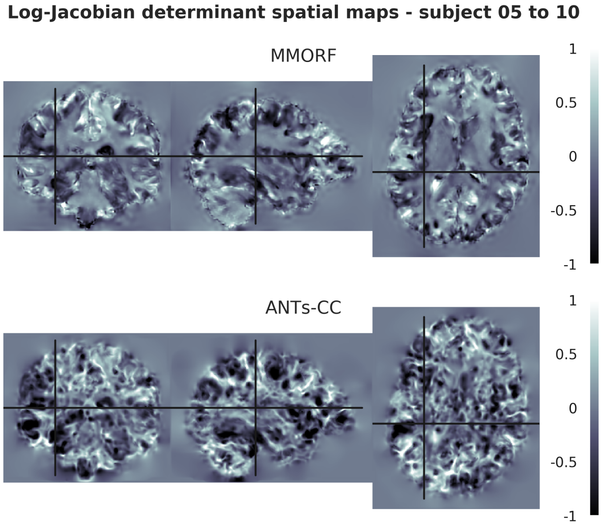

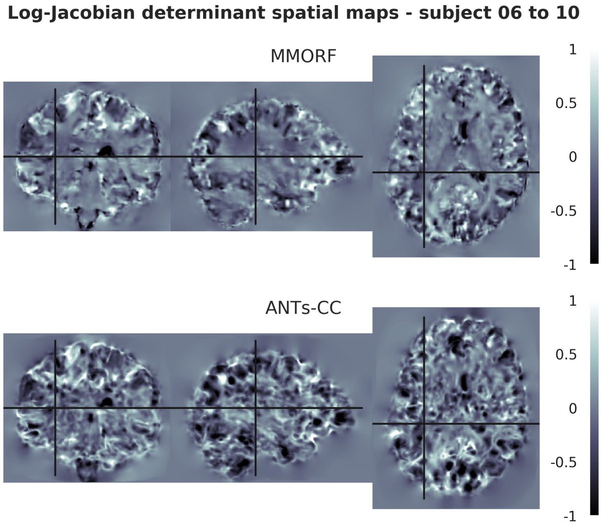

Log-Jacobian determinant spatial maps - subject 07 to 10

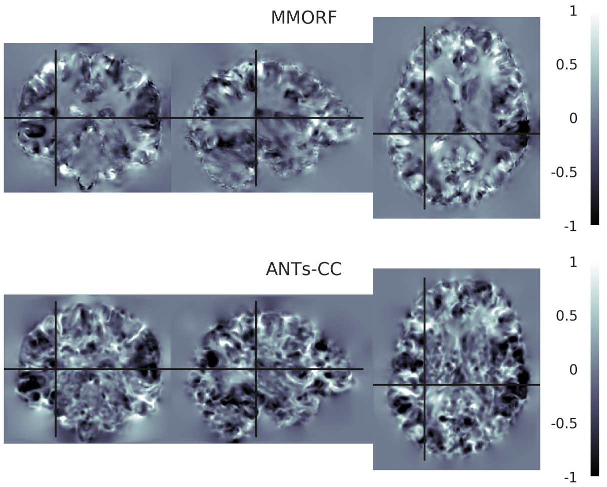

Log-Jacobian determinant spatial maps - subject 08 to 10

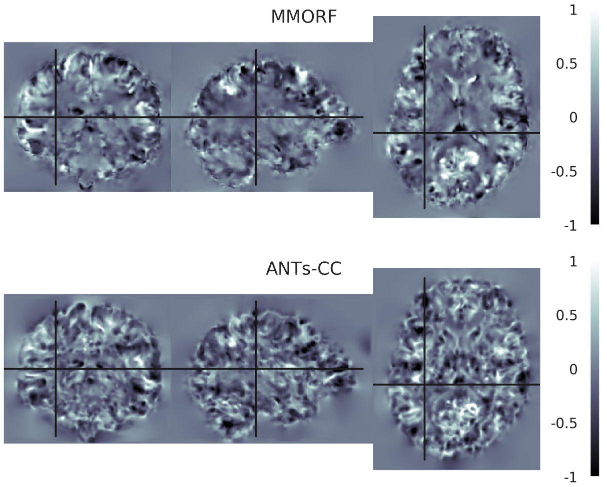

Log-Jacobian determinant spatial maps - subject 09 to 10

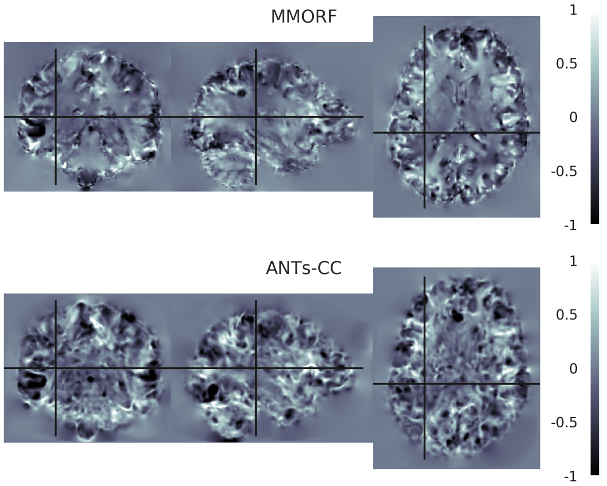

Log-Jacobian determinant spatial maps - subject 11 to 10

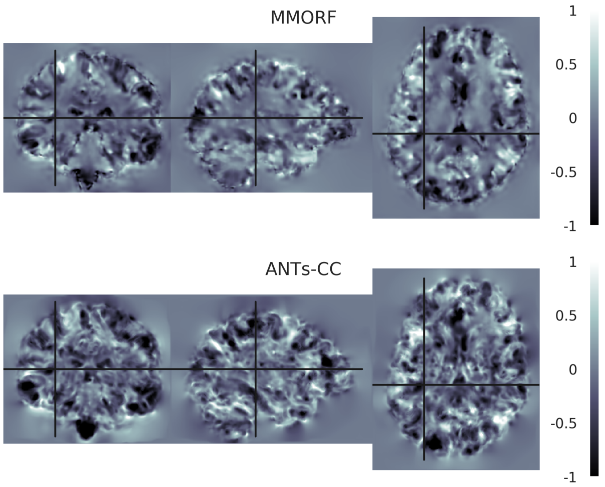

Log-Jacobian determinant spatial maps - subject 12 to 10

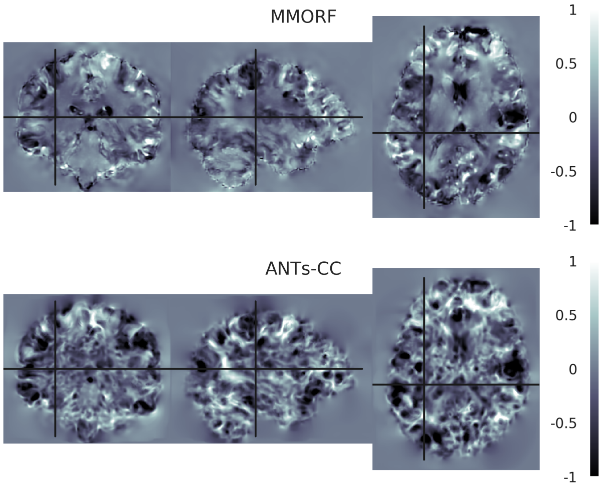

Log-Jacobian determinant spatial maps - subject 13 to 10

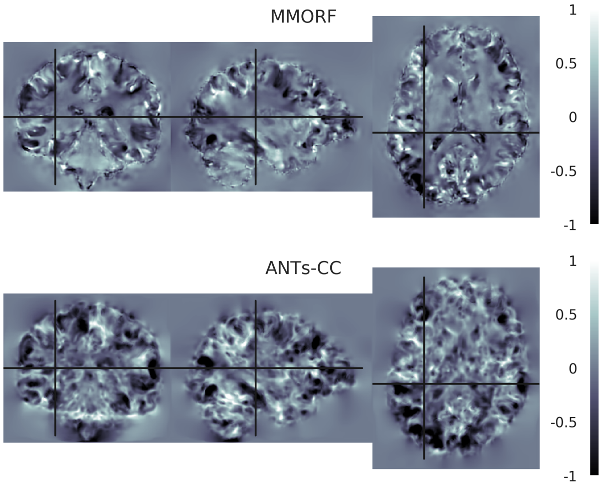

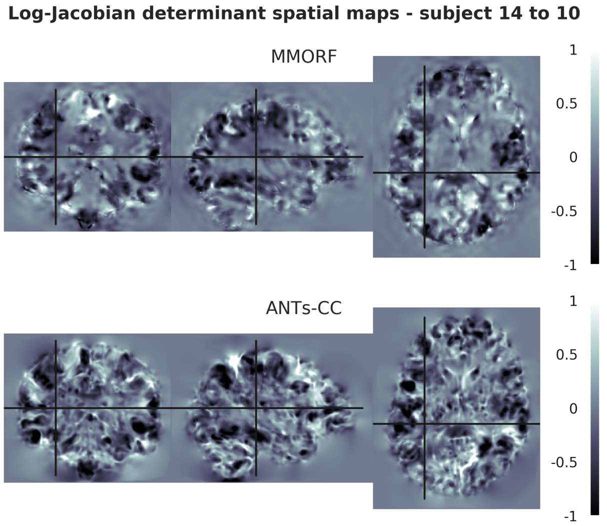
Log-Jacobian determinant spatial maps - subject 14 to 10
MMORF
ANTs-CC
1
0.5
0
-0.5
-1

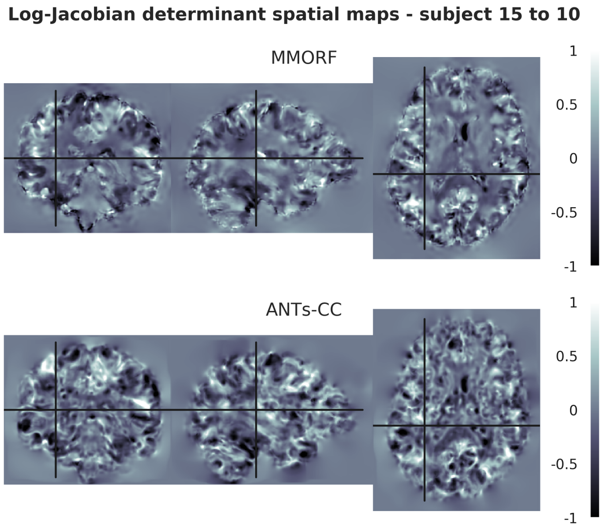
Log-Jacobian determinant spatial maps - subject 15 to 10
MMORF
ANTs-CC
1
0.5
0
-0.5
-1

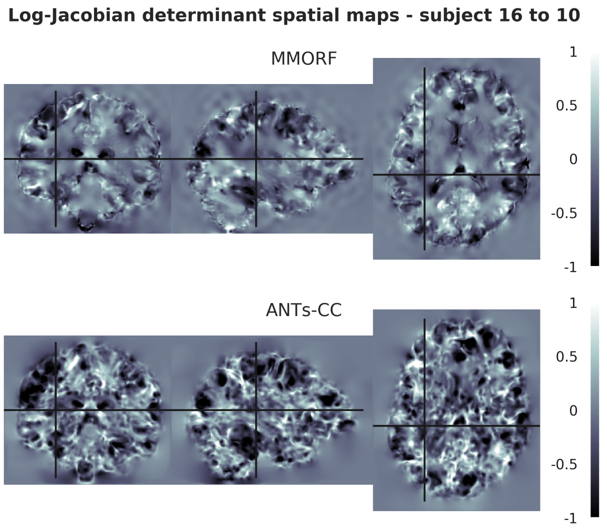
Log-Jacobian determinant spatial maps - subject 16 to 10
MMORF
ANTs-CC
1
0.5
0
-0.5
-1

11. Reference Subject 11

Log-Jacobian determinant spatial maps - subject 01 to 11

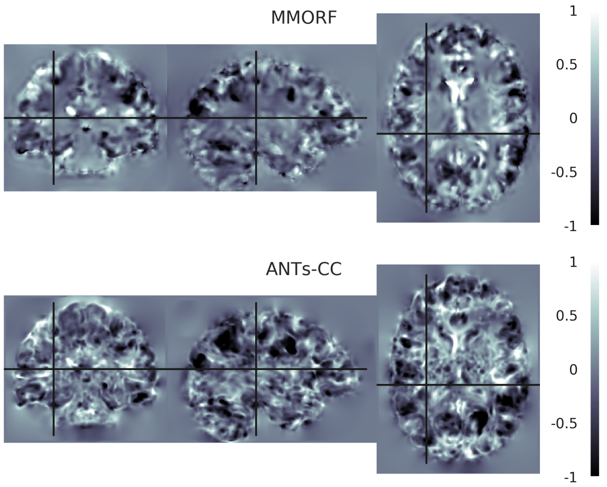

Log-Jacobian determinant spatial maps - subject 02 to 11

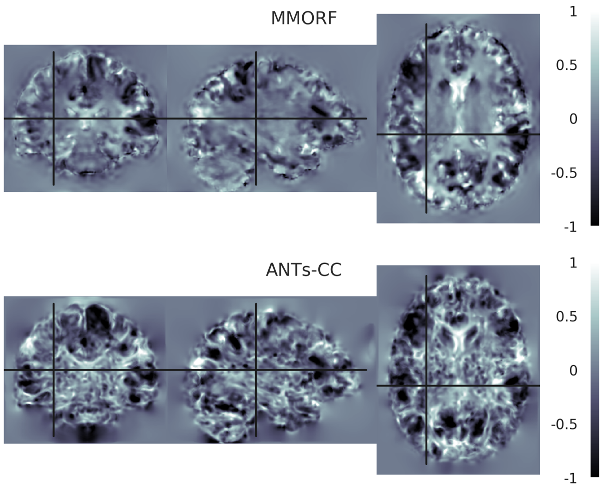

Log-Jacobian determinant spatial maps - subject 03 to 11

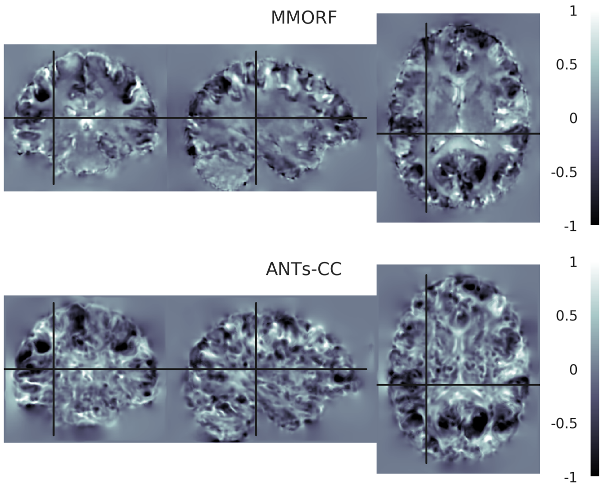

Log-Jacobian determinant spatial maps - subject 04 to 11

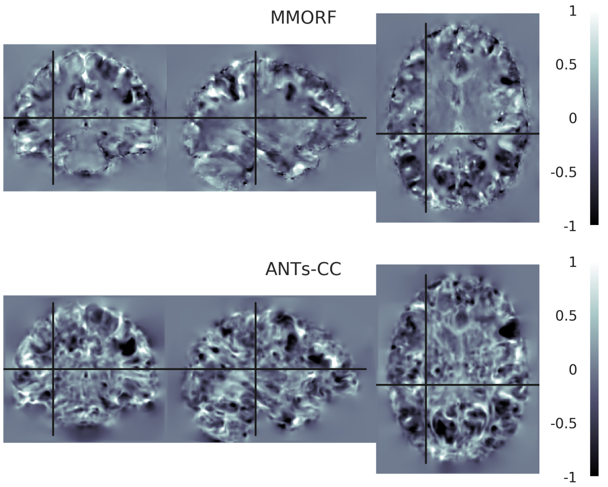

Log-Jacobian determinant spatial maps - subject 05 to 11

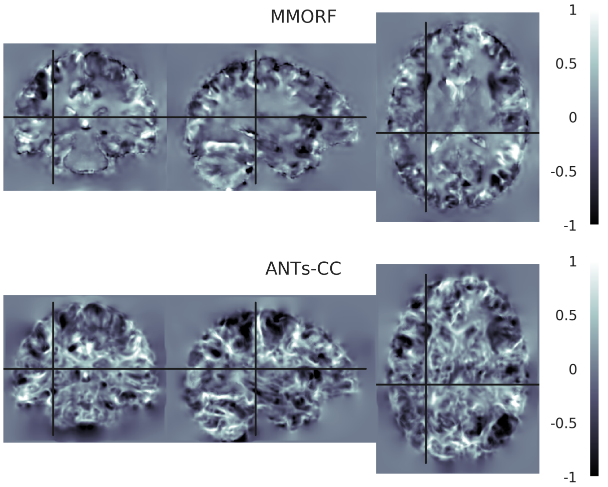

Log-Jacobian determinant spatial maps - subject 06 to 11

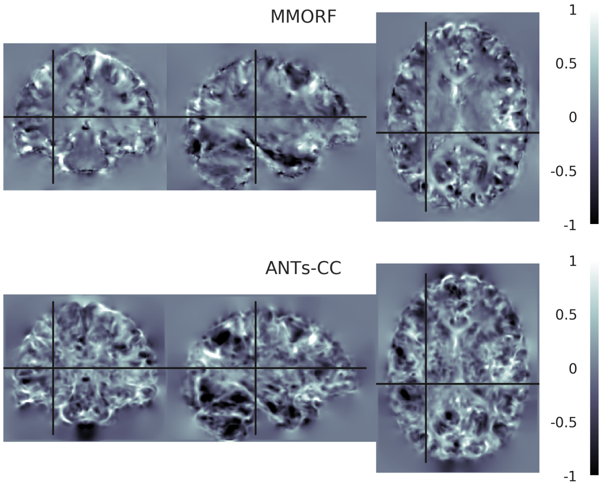

Log-Jacobian determinant spatial maps - subject 07 to 11

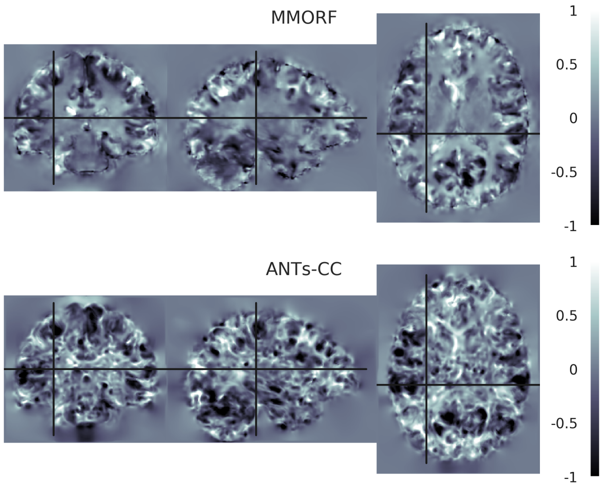

Log-Jacobian determinant spatial maps - subject 08 to 11

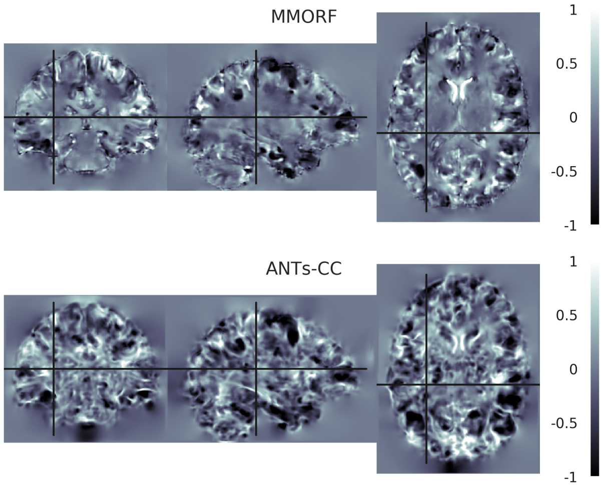

Log-Jacobian determinant spatial maps - subject 09 to 11

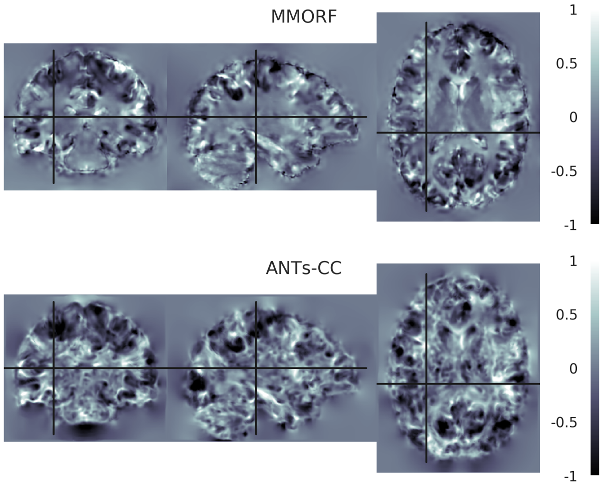

Log-Jacobian determinant spatial maps - subject 10 to 11

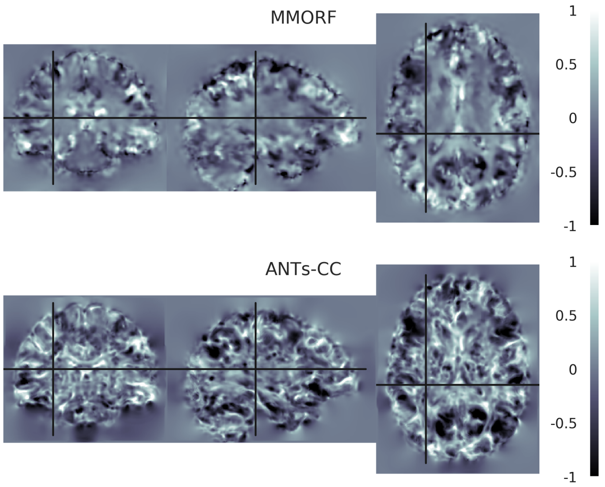

Log-Jacobian determinant spatial maps - subject 12 to 11

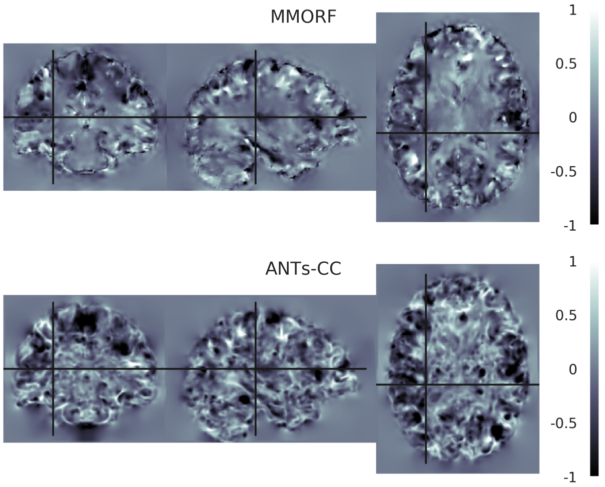

Log-Jacobian determinant spatial maps - subject 13 to 11

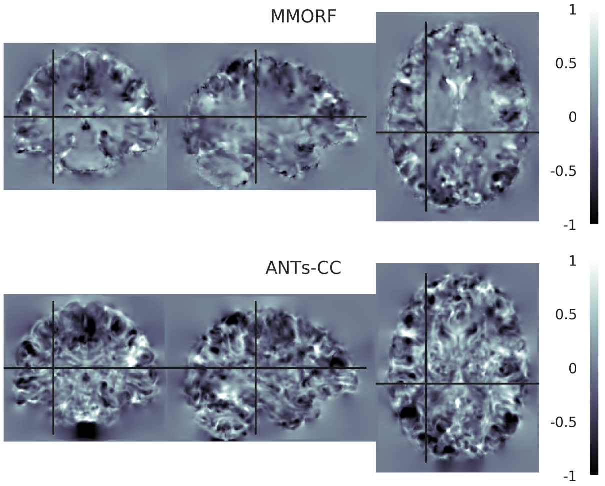

Log-Jacobian determinant spatial maps - subject 14 to 11

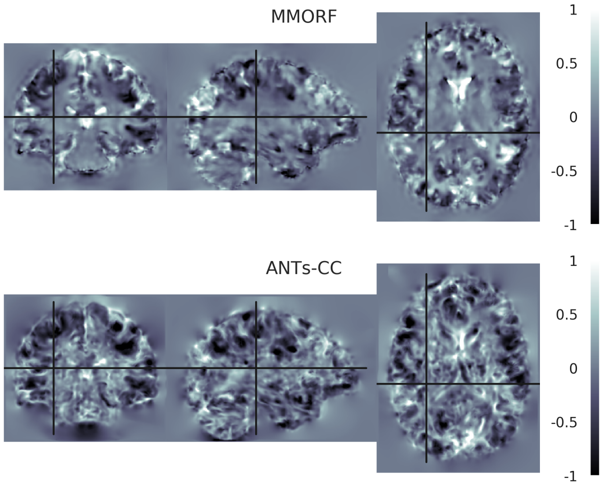

Log-Jacobian determinant spatial maps - subject 15 to 11

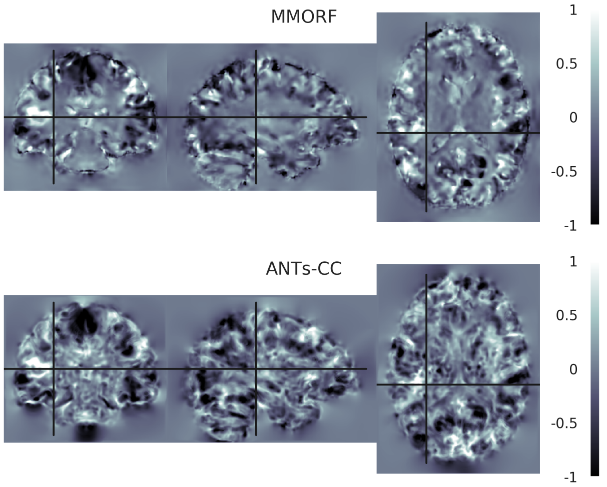

Log-Jacobian determinant spatial maps - subject 16 to 11

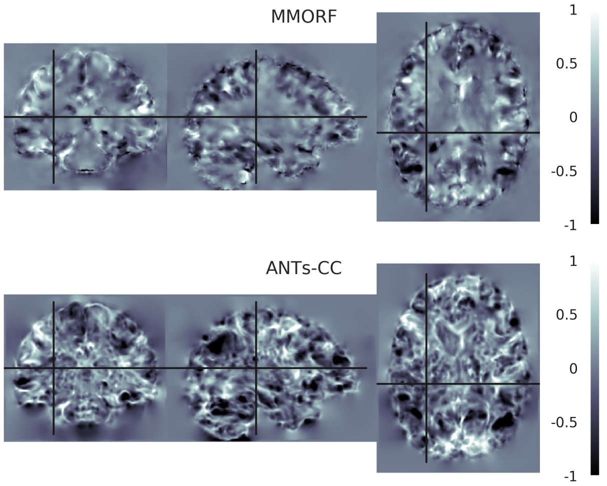

12. Reference Subject 12

Log-Jacobian determinant spatial maps - subject 01 to 12

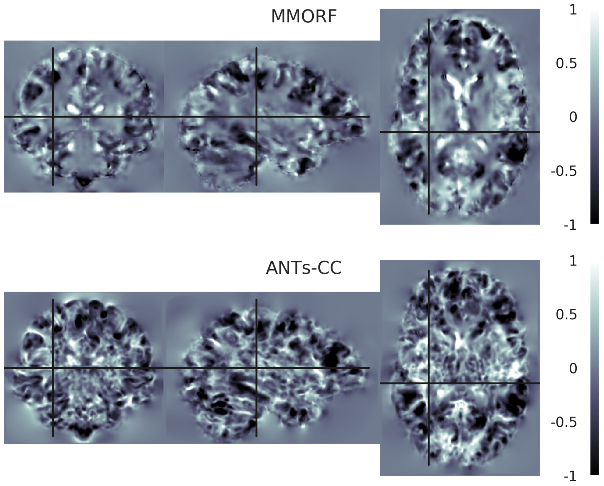

Log-Jacobian determinant spatial maps - subject 02 to 12

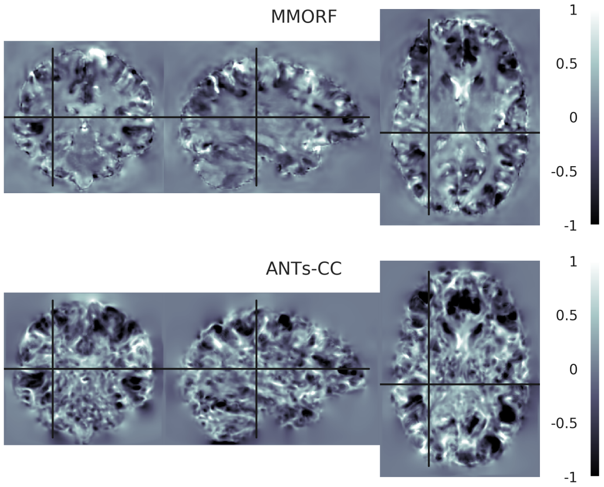

Log-Jacobian determinant spatial maps - subject 03 to 12

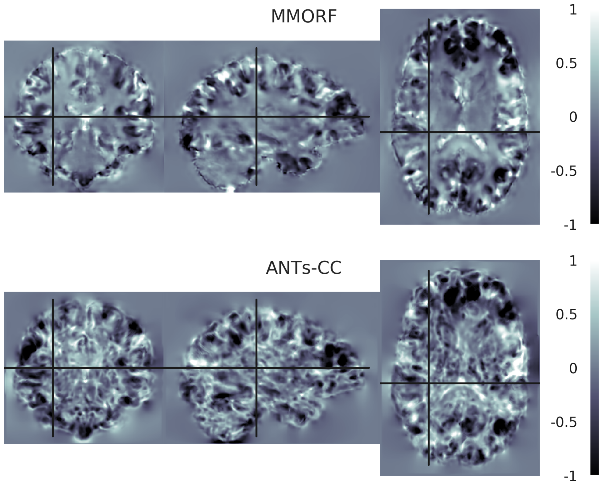

Log-Jacobian determinant spatial maps - subject 04 to 12

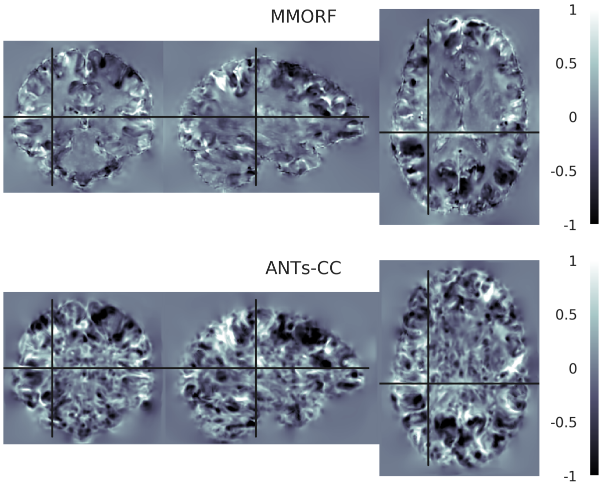

Log-Jacobian determinant spatial maps - subject 05 to 12

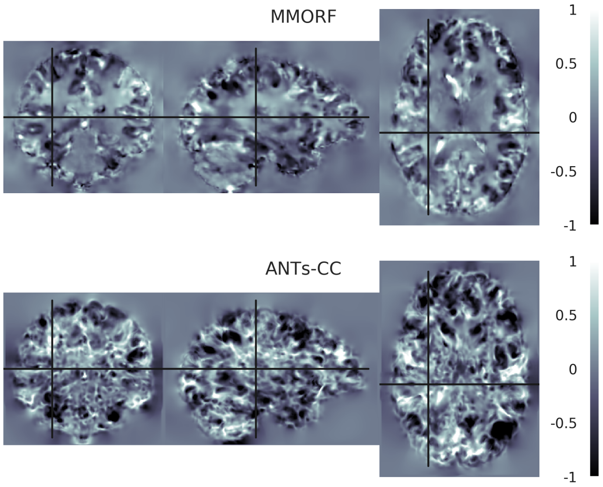

Log-Jacobian determinant spatial maps - subject 06 to 12

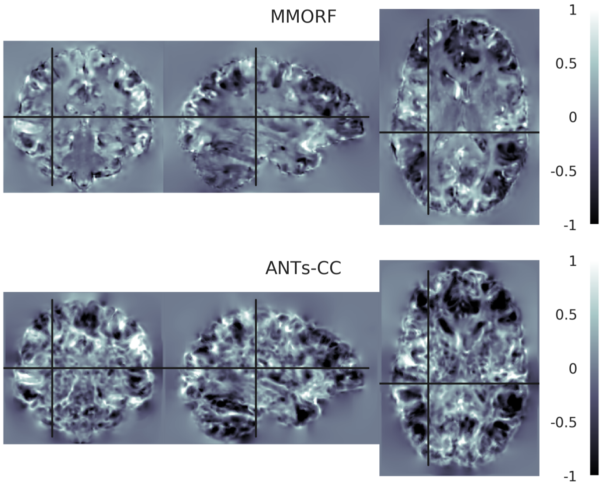

Log-Jacobian determinant spatial maps - subject 07 to 12

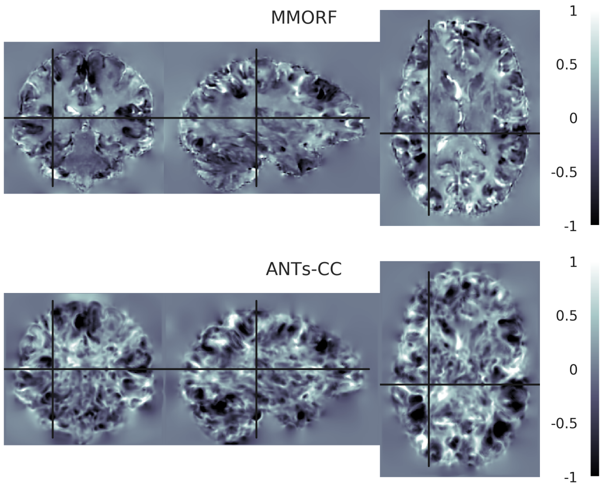

Log-Jacobian determinant spatial maps - subject 10 to 12

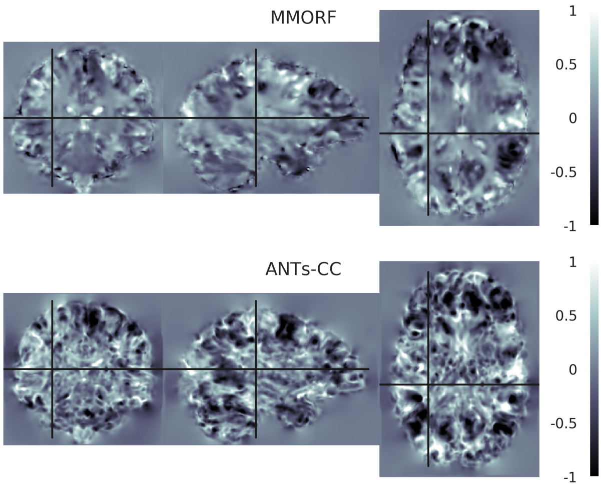

Log-Jacobian determinant spatial maps - subject 08 to 12

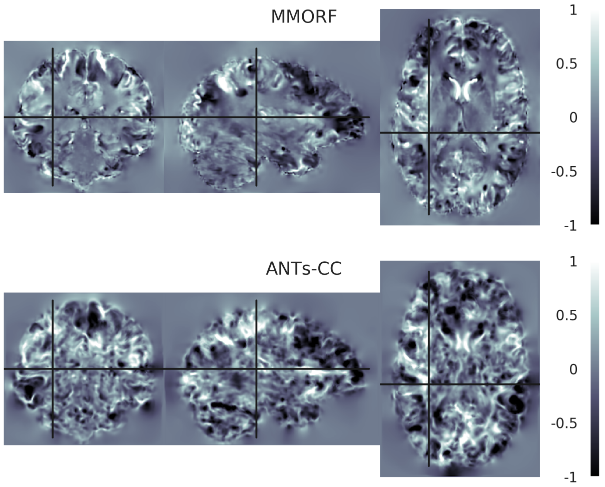

Log-Jacobian determinant spatial maps - subject 11 to 12

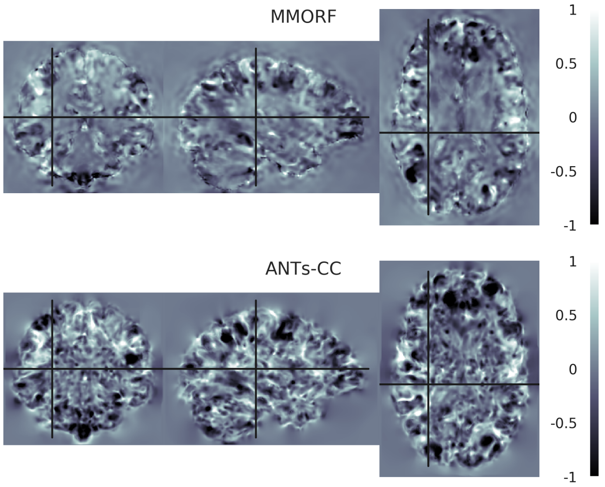

Log-Jacobian determinant spatial maps - subject 09 to 12

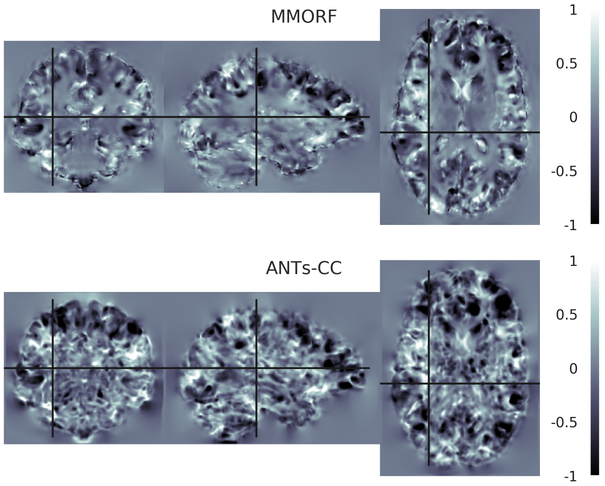

Log-Jacobian determinant spatial maps - subject 13 to 12

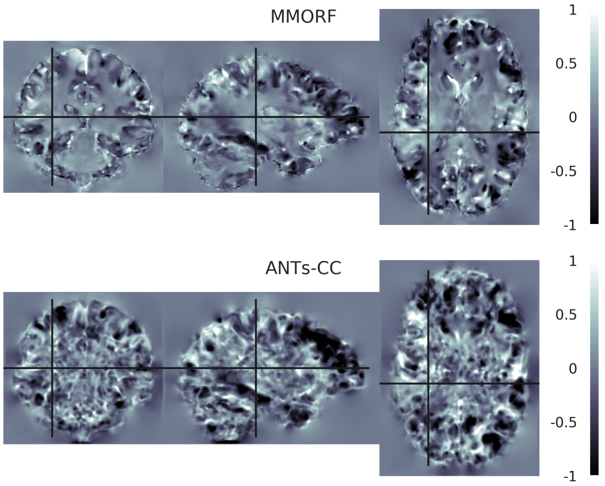

Log-Jacobian determinant spatial maps - subject 14 to 12

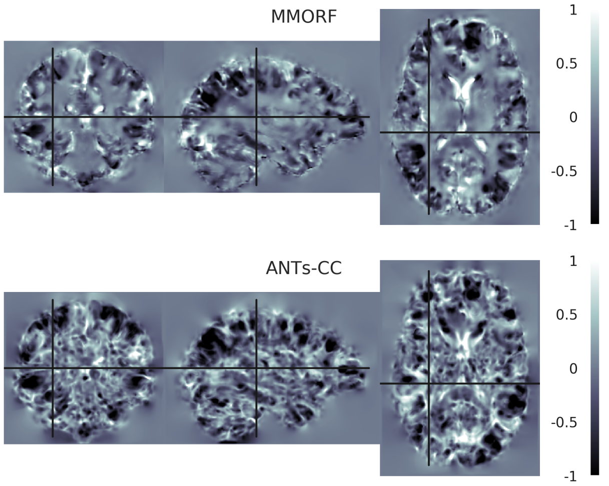

Log-Jacobian determinant spatial maps - subject 15 to 12

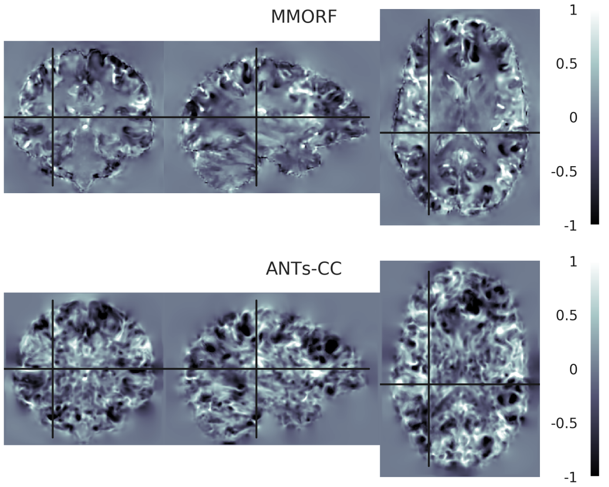

Log-Jacobian determinant spatial maps - subject 16 to 12

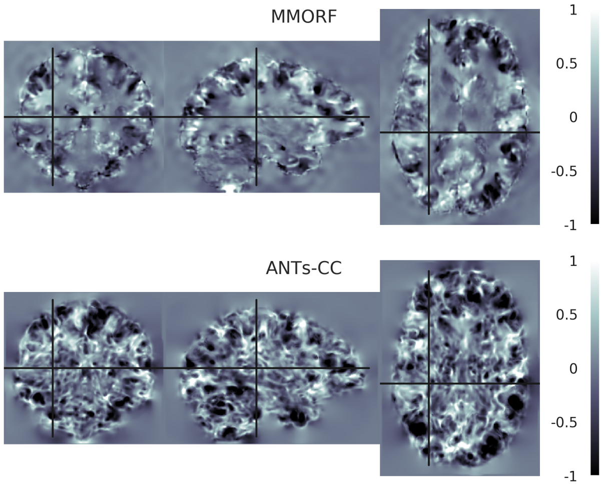

13. Reference Subject 13

Log-Jacobian determinant spatial maps - subject 01 to 13

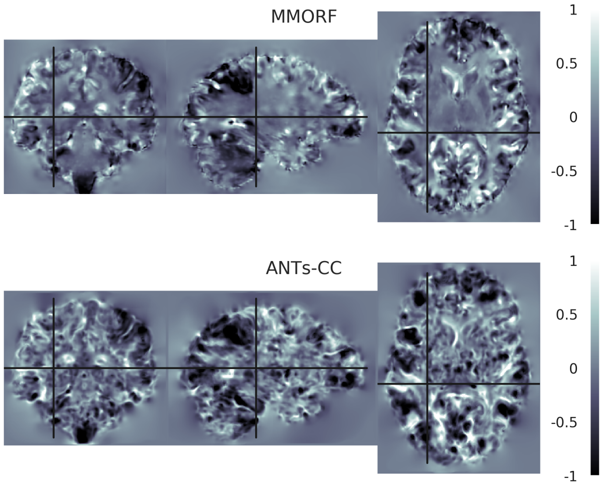

Log-Jacobian determinant spatial maps - subject 02 to 13

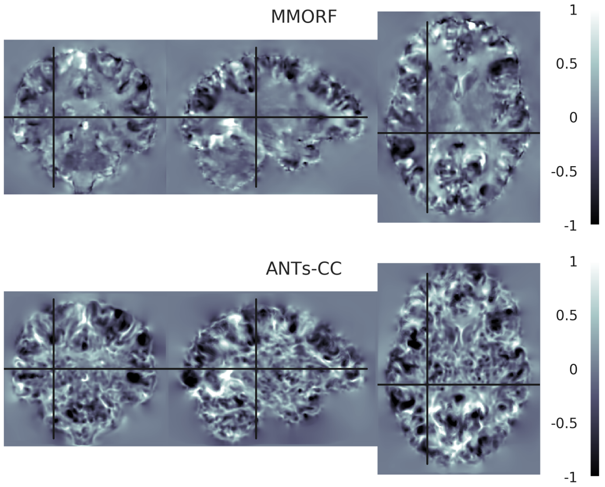

Log-Jacobian determinant spatial maps - subject 03 to 13

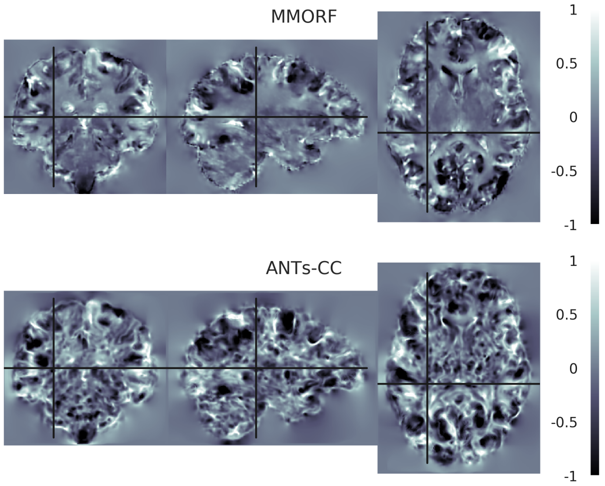

Log-Jacobian determinant spatial maps - subject 04 to 13

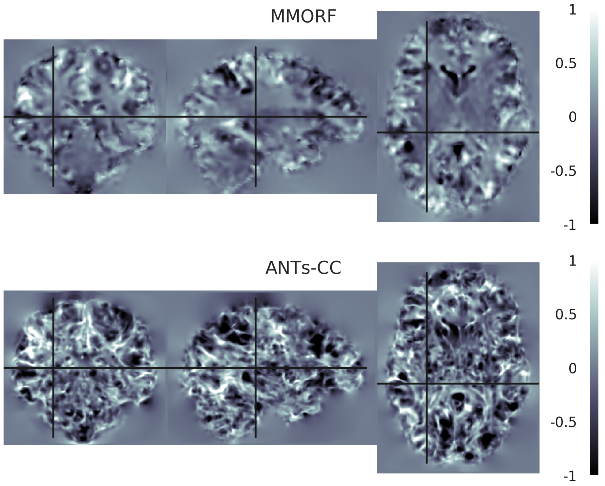

Log-Jacobian determinant spatial maps - subject 05 to 13

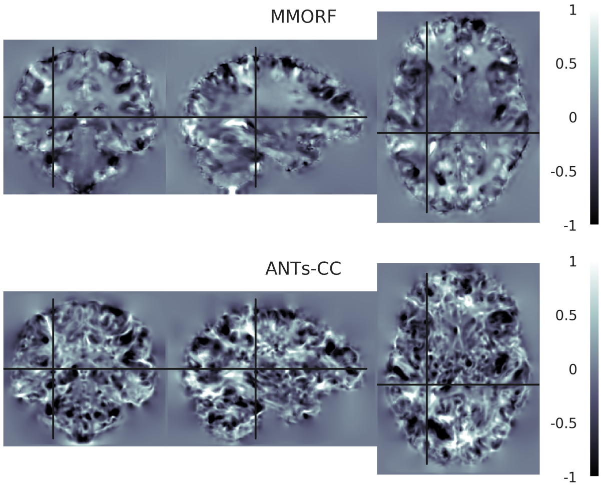

Log-Jacobian determinant spatial maps - subject 06 to 13

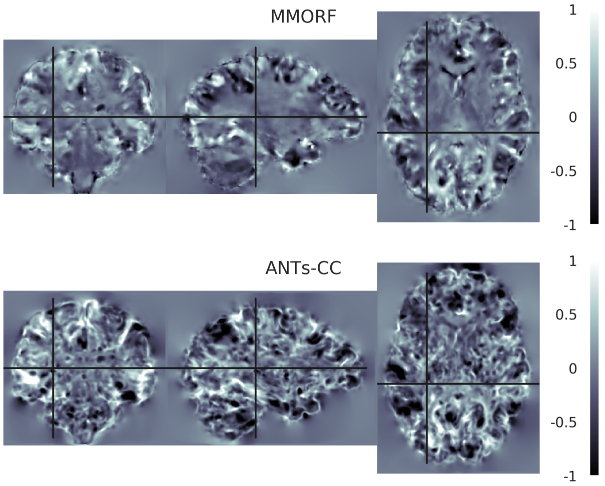

Log-Jacobian determinant spatial maps - subject 07 to 13

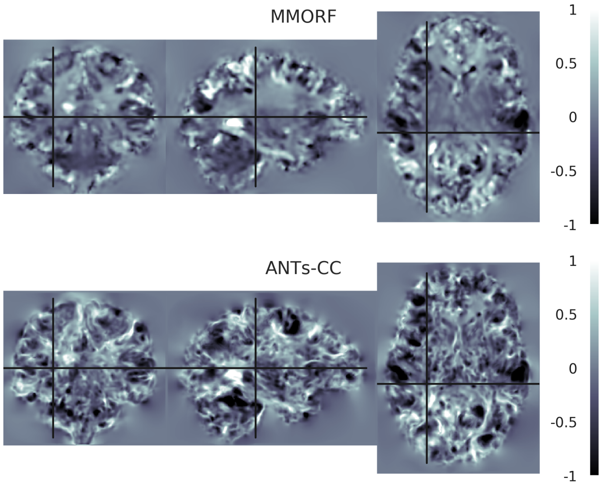

Log-Jacobian determinant spatial maps - subject 10 to 13

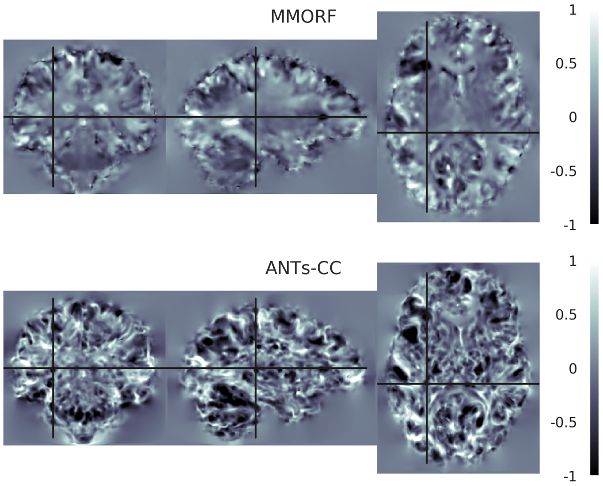

Log-Jacobian determinant spatial maps - subject 08 to 13

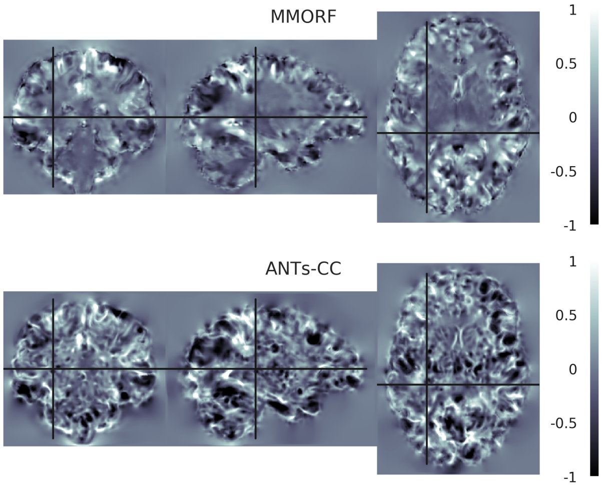

Log-Jacobian determinant spatial maps - subject 11 to 13

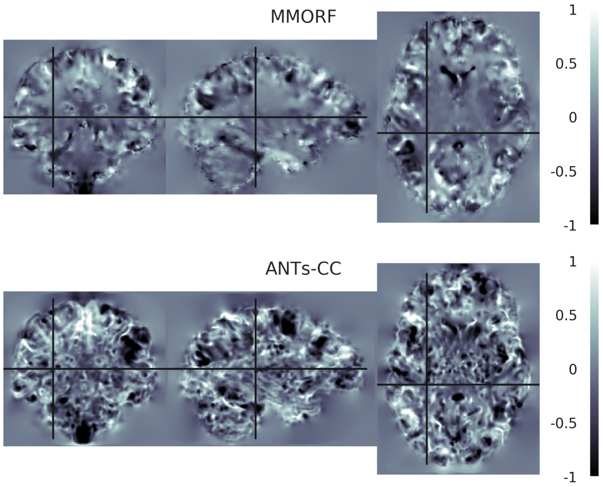

Log-Jacobian determinant spatial maps - subject 09 to 13

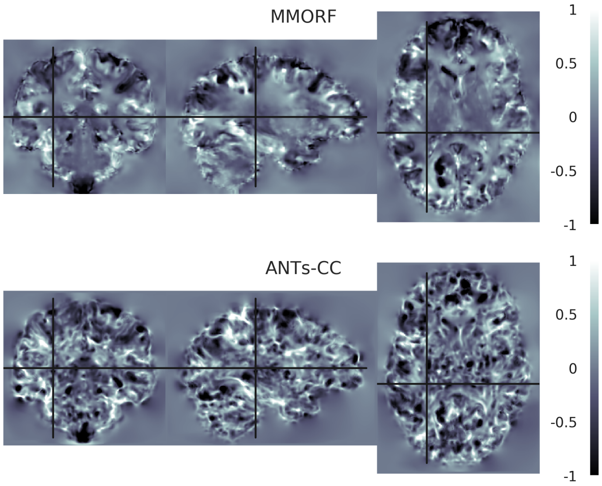

Log-Jacobian determinant spatial maps - subject 12 to 13

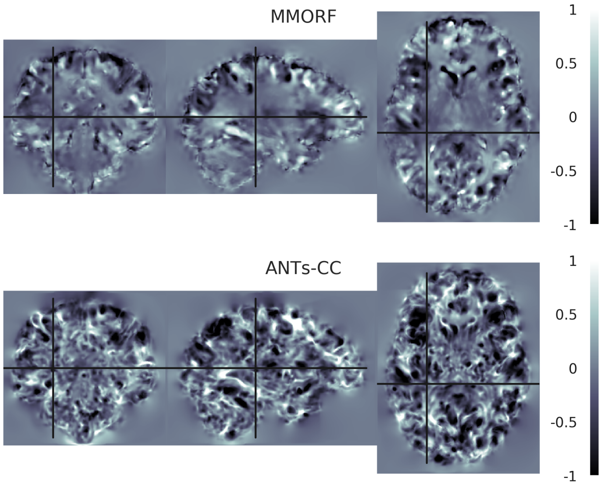

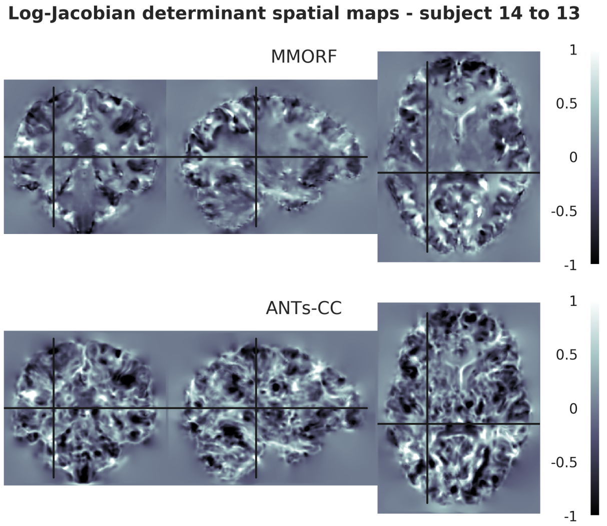
Log-Jacobian determinant spatial maps - subject 14 to 13
MMORF
ANTs-CC
1
0.5
0
-0.5
-1

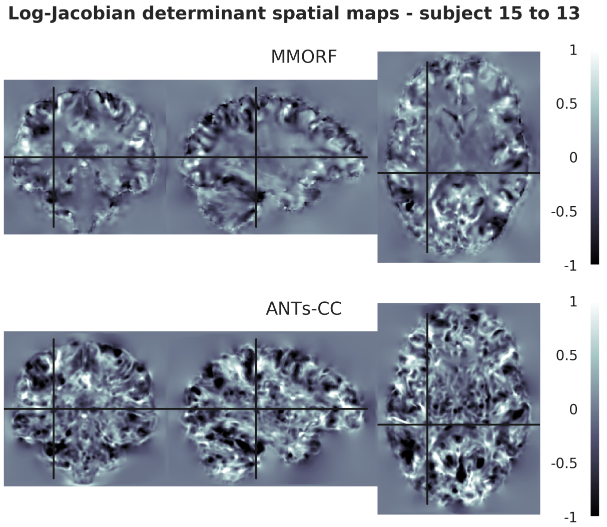
Log-Jacobian determinant spatial maps - subject 15 to 13
MMORF
ANTs-CC
1
0.5
0
-0.5
-1

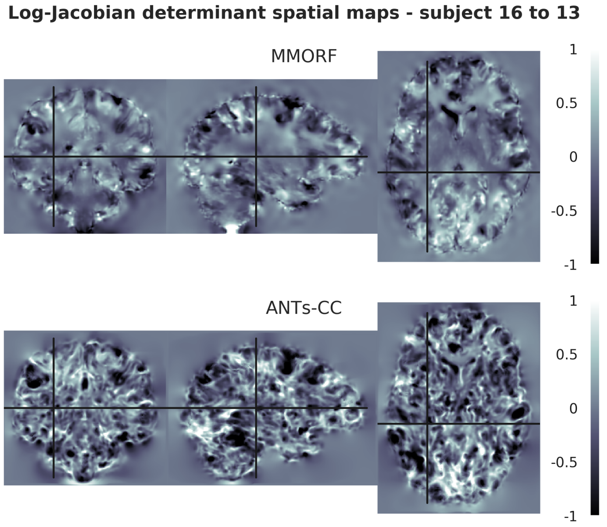
Log-Jacobian determinant spatial maps - subject 16 to 13
MMORF
ANTs-CC
1
0.5
0
-0.5
-1

14. Reference Subject 14

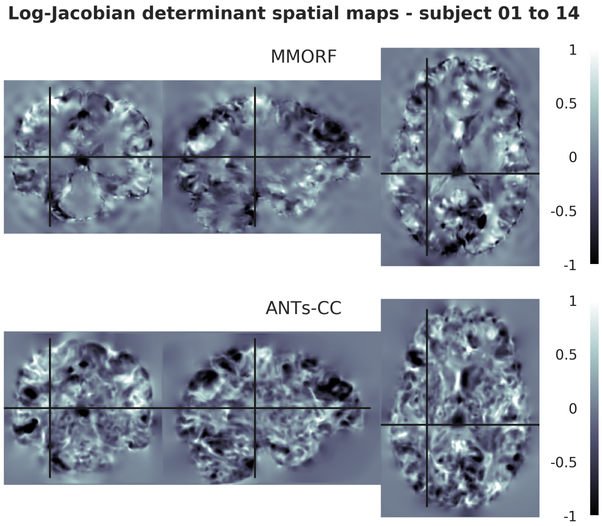

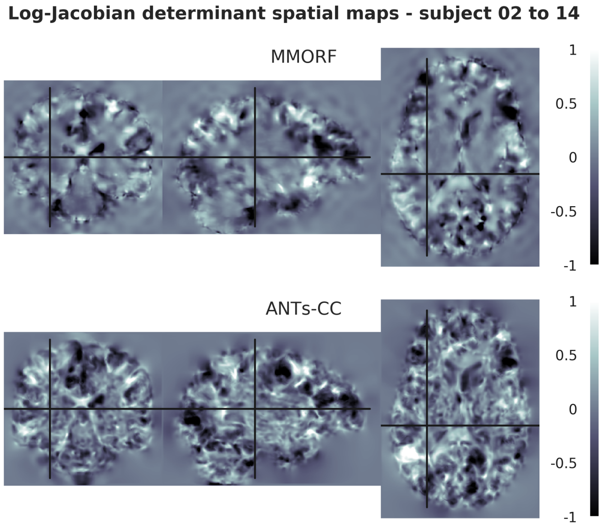

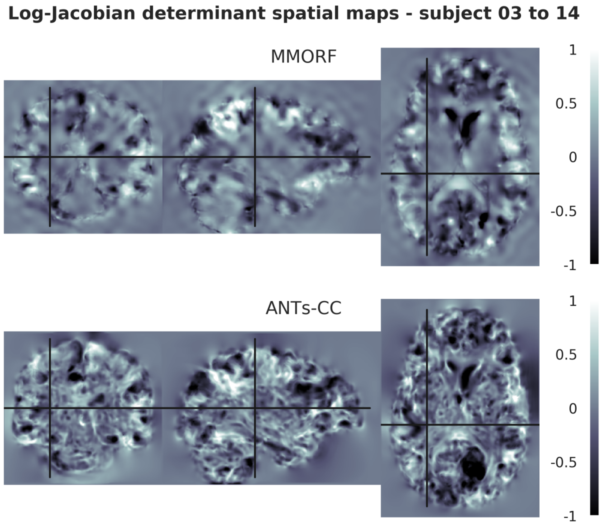

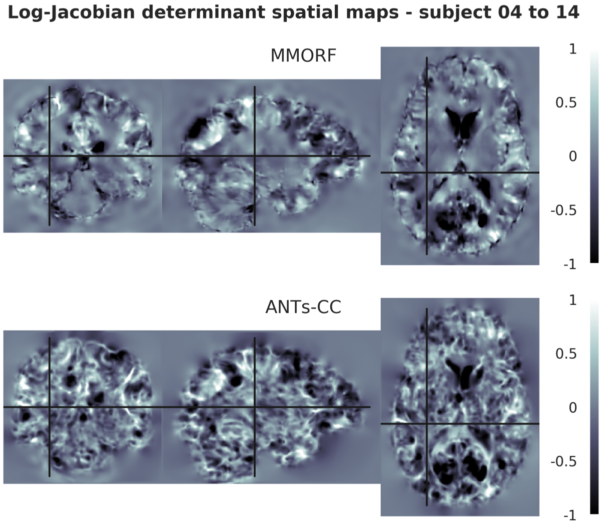

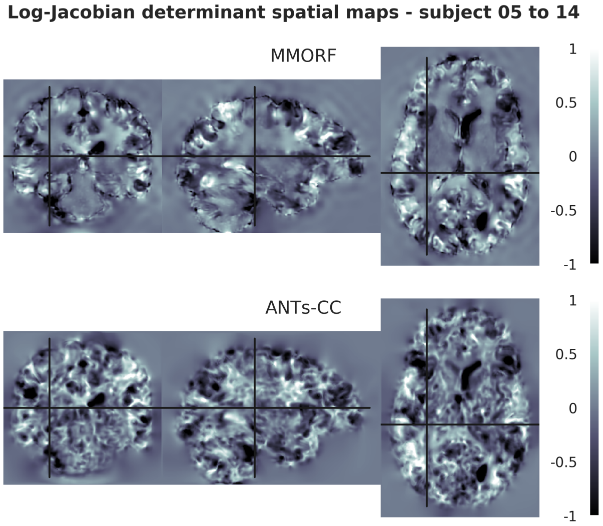

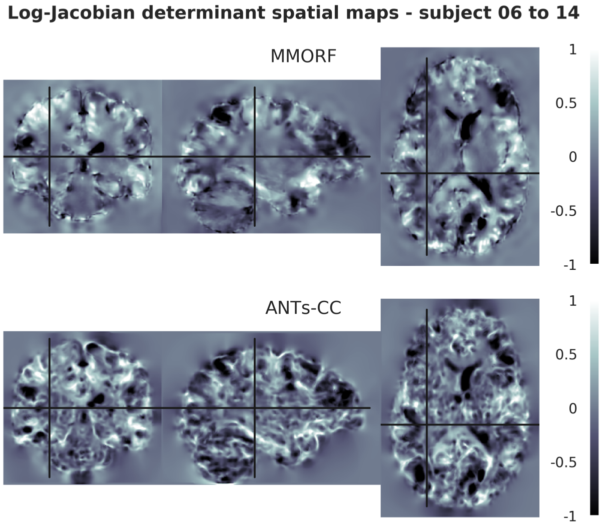

Log-Jacobian determinant spatial maps - subject 07 to 14

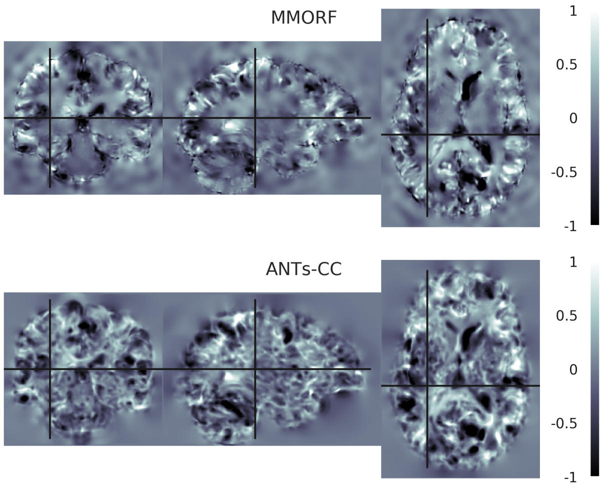

Log-Jacobian determinant spatial maps - subject 08 to 14

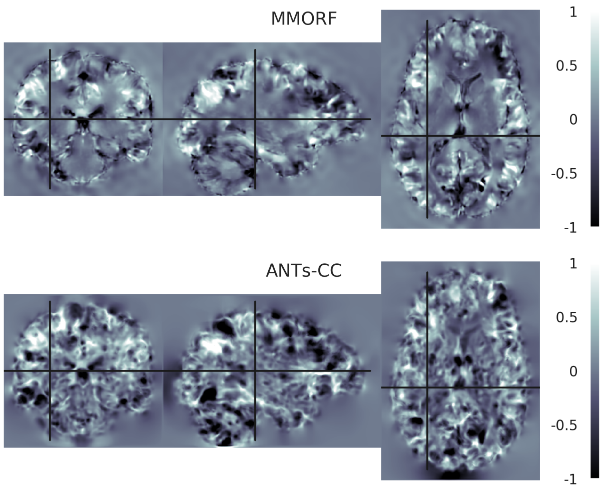

Log-Jacobian determinant spatial maps - subject 09 to 14

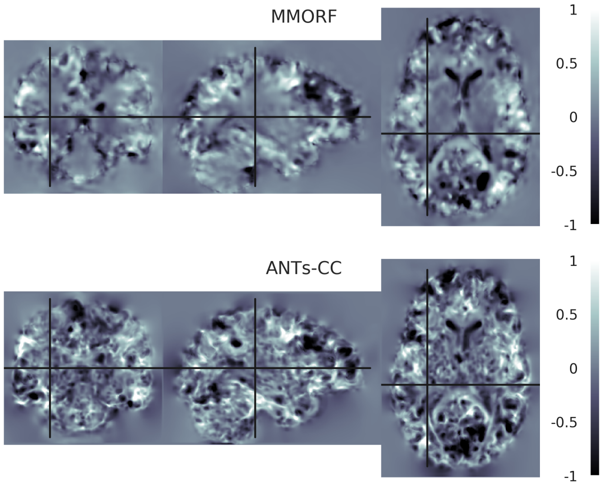

Log-Jacobian determinant spatial maps - subject 10 to 14

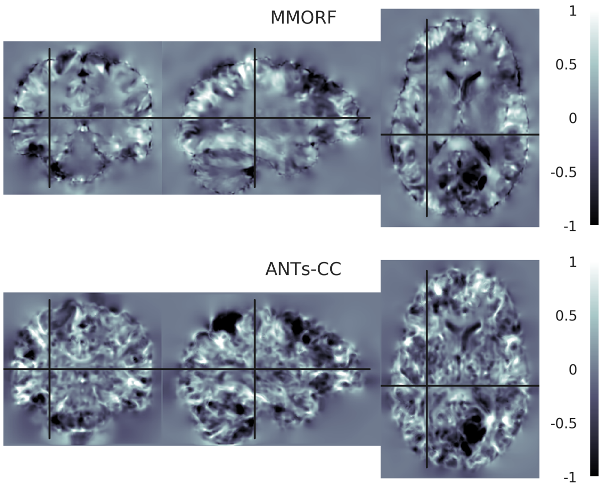

Log-Jacobian determinant spatial maps - subject 11 to 14

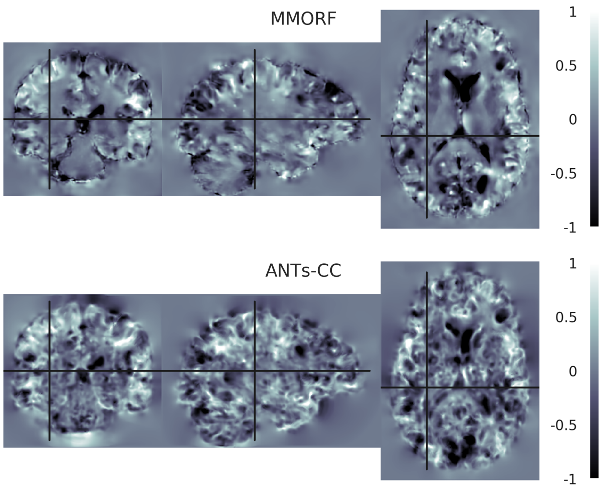

Log-Jacobian determinant spatial maps - subject 12 to 14

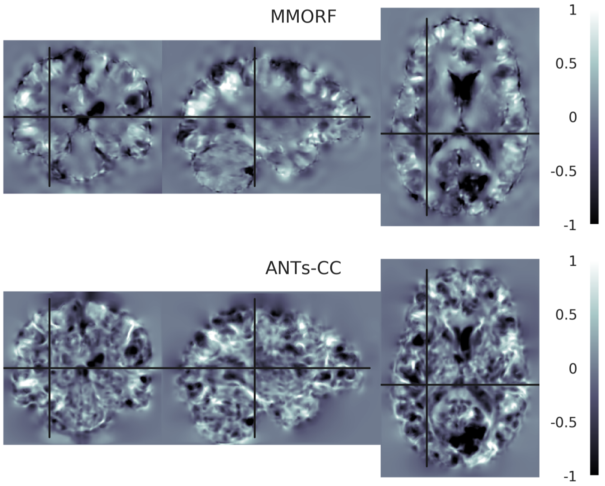

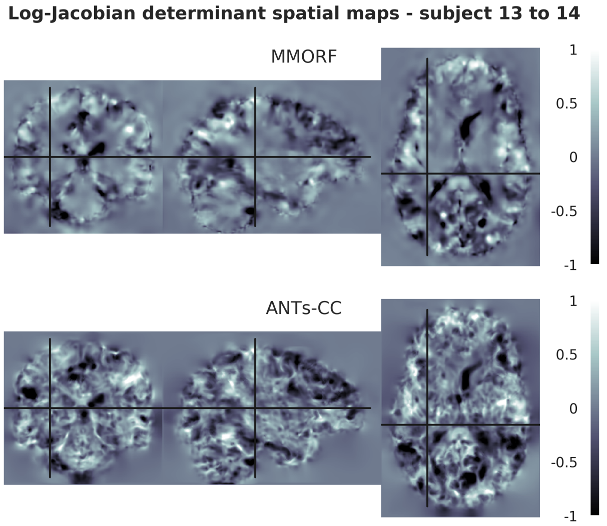
Log-Jacobian determinant spatial maps - subject 13 to 14
MMORF
ANTs-CC
1
0.5
0
-0.5
-1

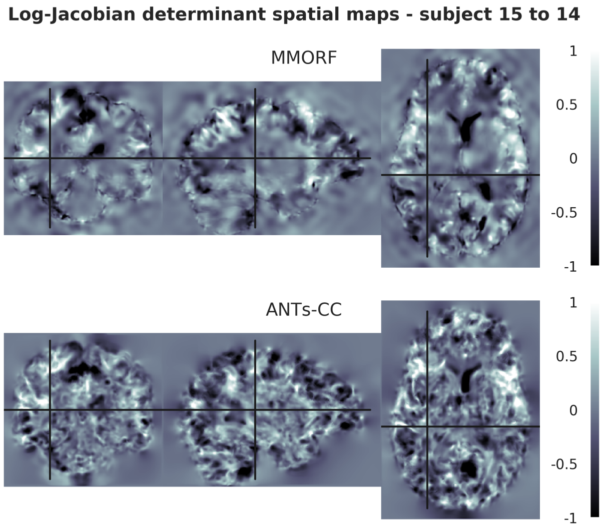
Log-Jacobian determinant spatial maps - subject 15 to 14
MMORF
ANTs-CC
1
0.5
0
-0.5
-1

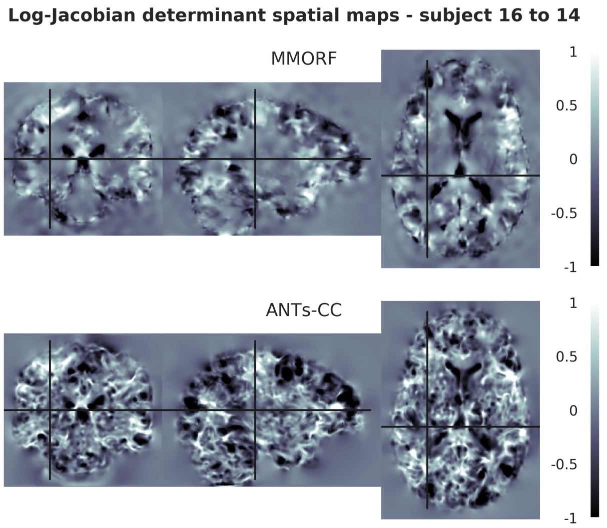
Log-Jacobian determinant spatial maps - subject 16 to 14
MMORF
ANTs-CC
1
0.5
0
-0.5
-1

15. Reference Subject 15

Log-Jacobian determinant spatial maps - subject 01 to 15

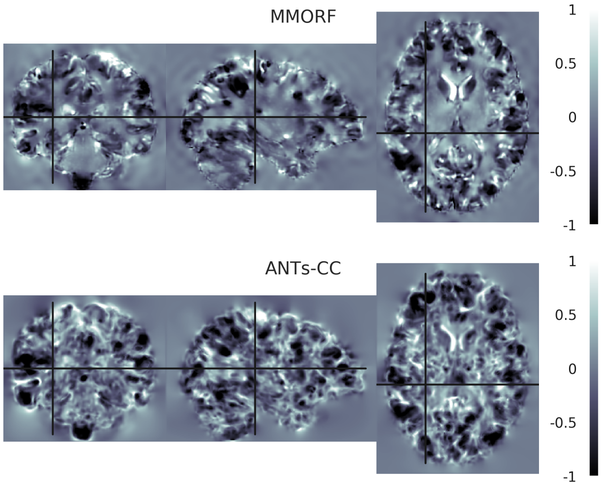

Log-Jacobian determinant spatial maps - subject 02 to 15

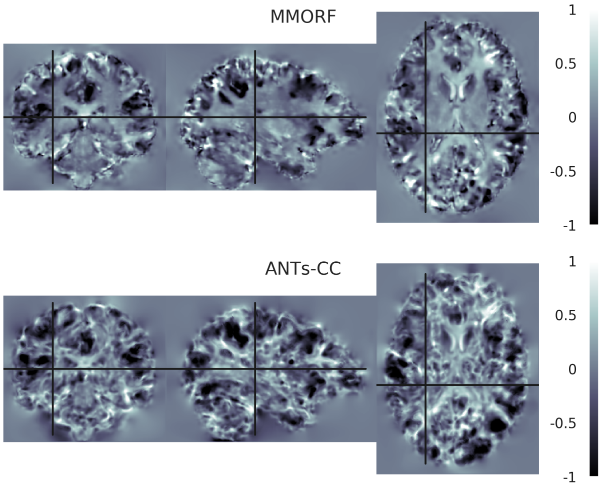

Log-Jacobian determinant spatial maps - subject 03 to 15

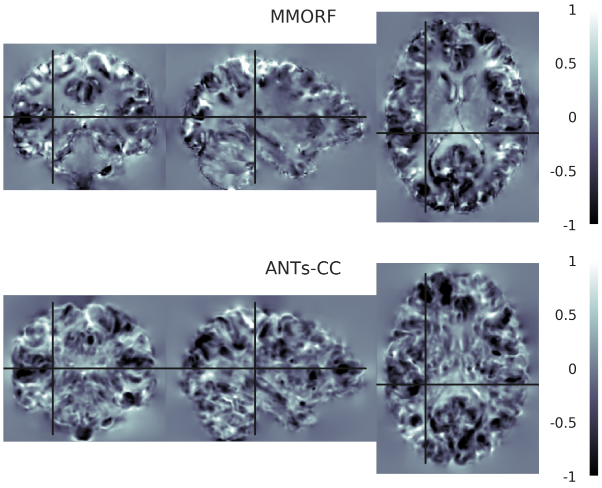

Log-Jacobian determinant spatial maps - subject 04 to 15

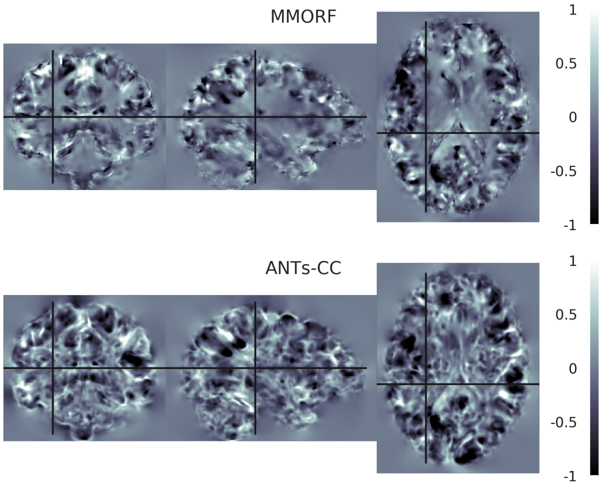

Log-Jacobian determinant spatial maps - subject 05 to 15

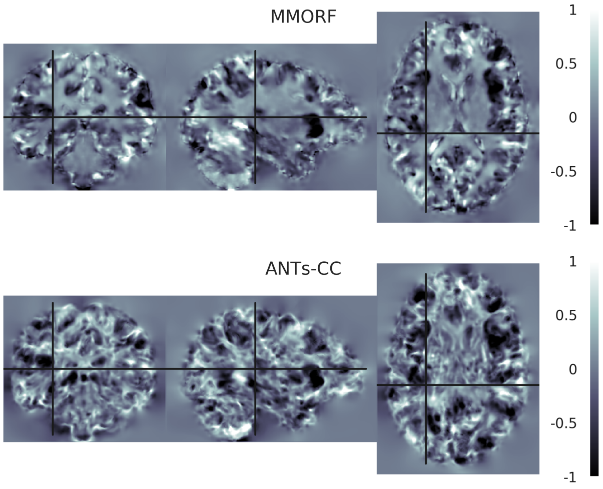

Log-Jacobian determinant spatial maps - subject 06 to 15

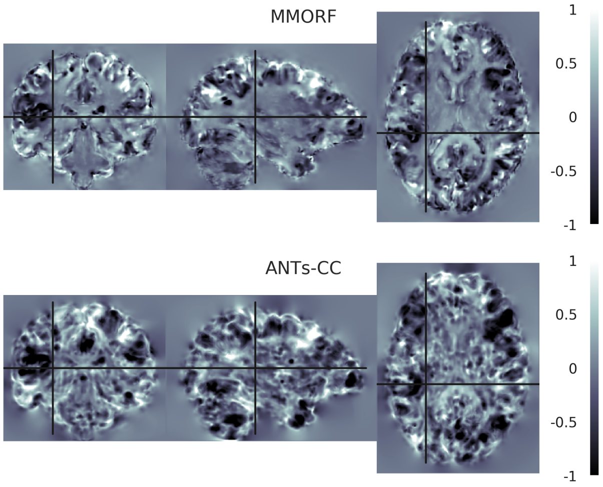

Log-Jacobian determinant spatial maps - subject 07 to 15

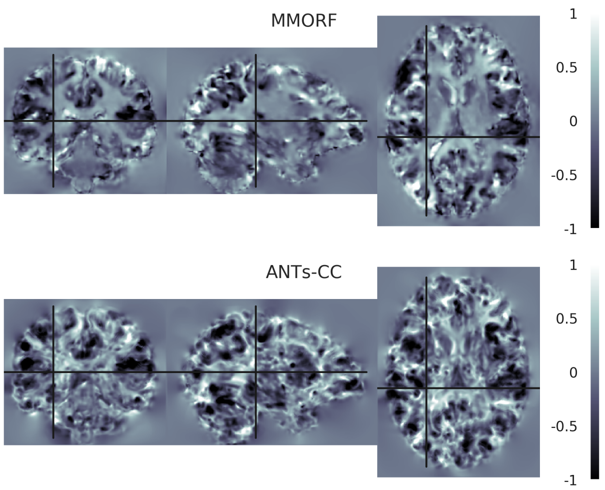

Log-Jacobian determinant spatial maps - subject 08 to 15

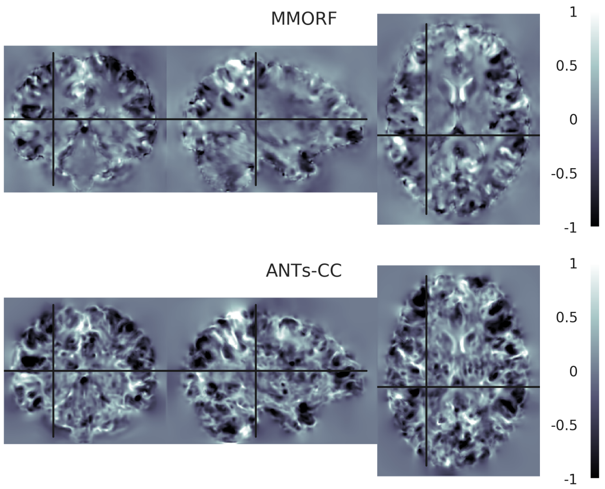

Log-Jacobian determinant spatial maps - subject 09 to 15

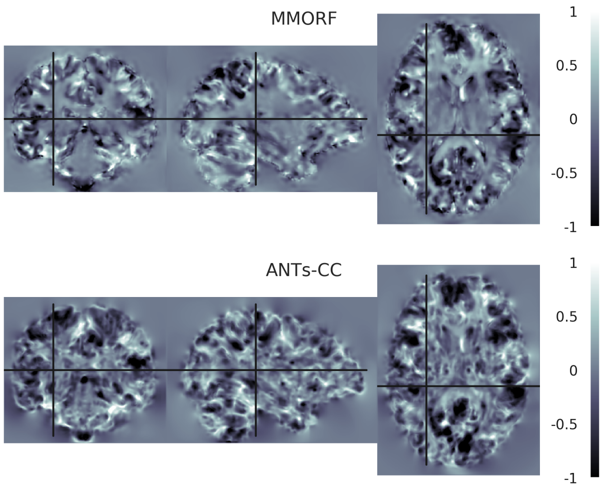

Log-Jacobian determinant spatial maps - subject 10 to 15

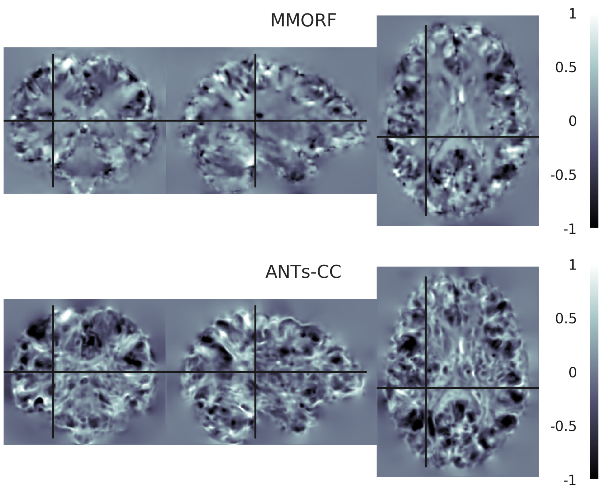

Log-Jacobian determinant spatial maps - subject 11 to 15

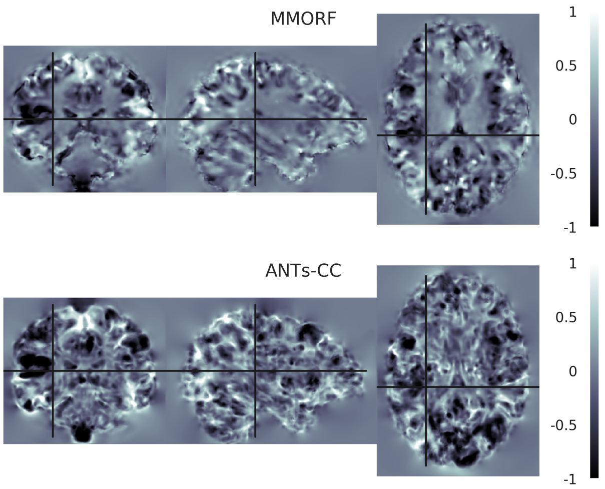

Log-Jacobian determinant spatial maps - subject 12 to 15

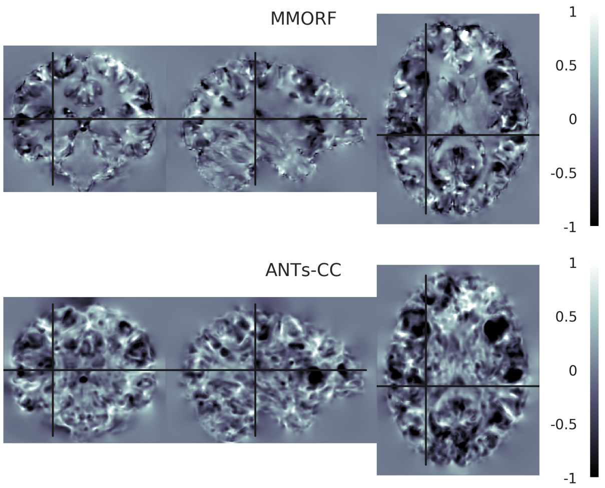

Log-Jacobian determinant spatial maps - subject 13 to 15

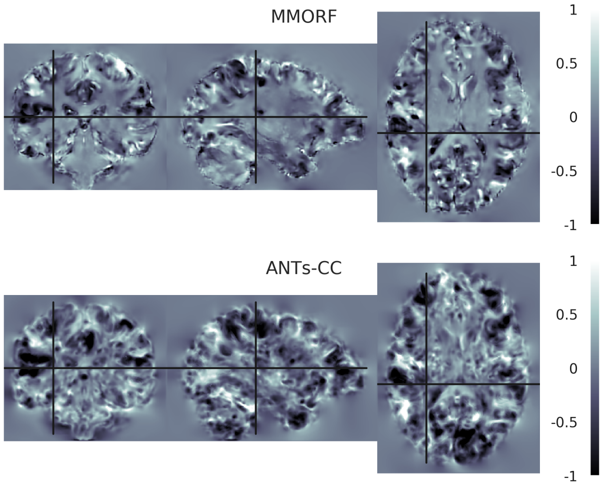

Log-Jacobian determinant spatial maps - subject 14 to 15

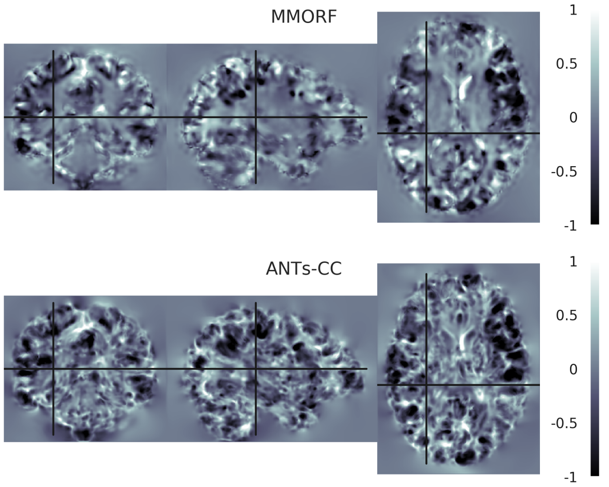

Log-Jacobian determinant spatial maps - subject 16 to 15

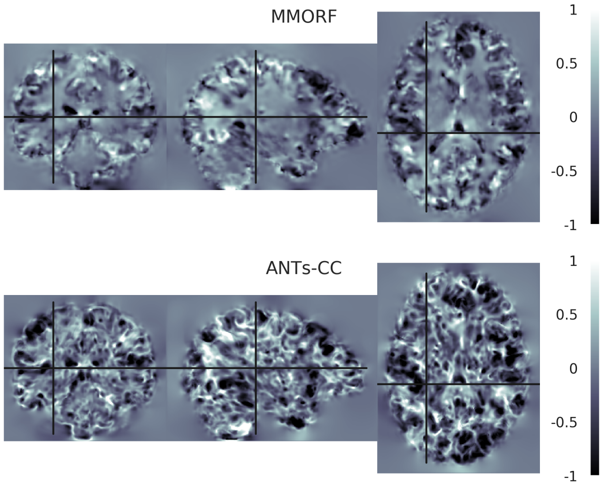

16. Reference Subject 16

Log-Jacobian determinant spatial maps - subject 01 to 16

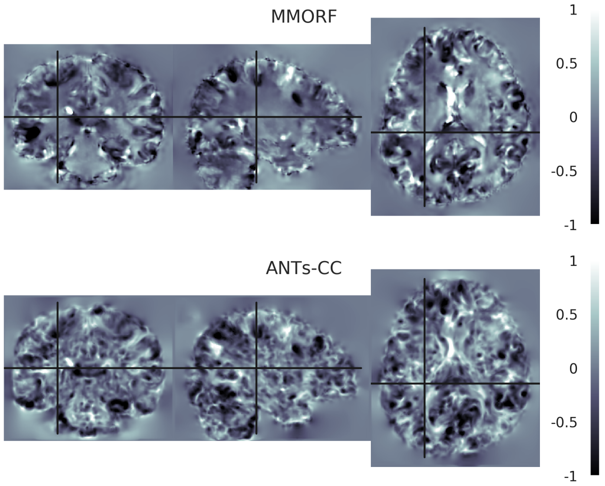

Log-Jacobian determinant spatial maps - subject 02 to 16

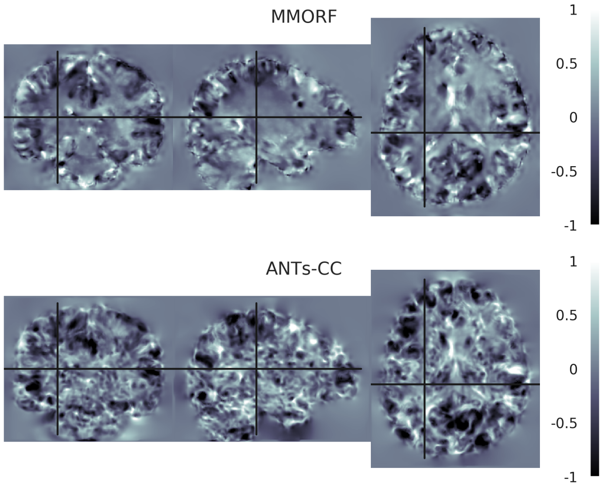

Log-Jacobian determinant spatial maps - subject 03 to 16

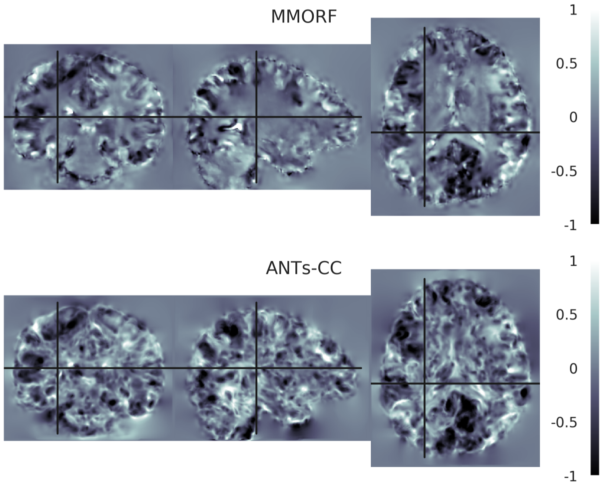

Log-Jacobian determinant spatial maps - subject 04 to 16

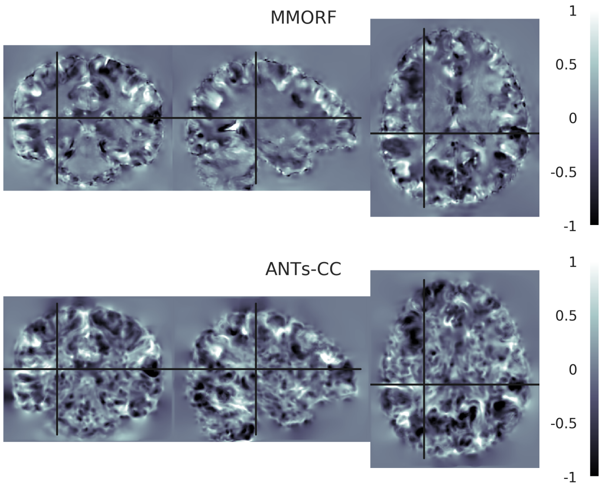

Log-Jacobian determinant spatial maps - subject 05 to 16

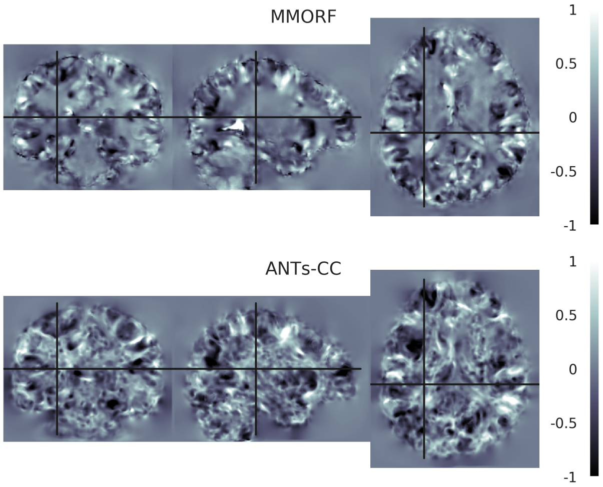

Log-Jacobian determinant spatial maps - subject 06 to 16

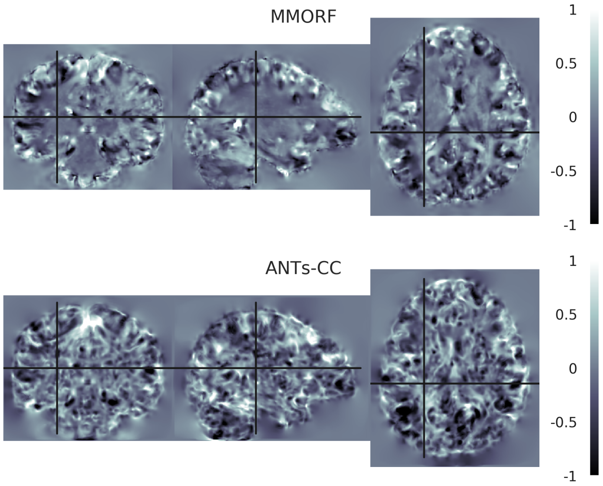

Log-Jacobian determinant spatial maps - subject 07 to 16

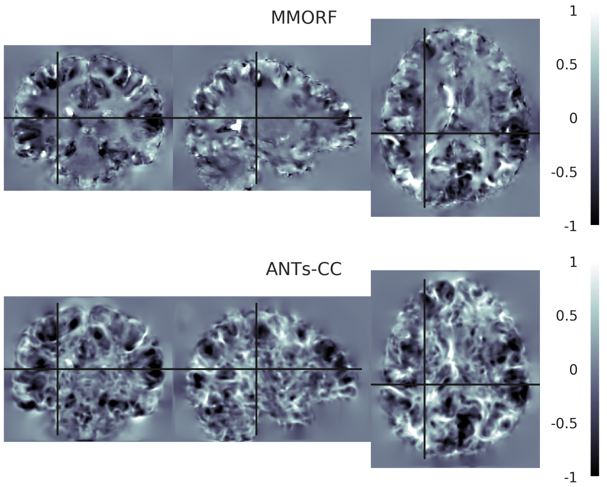

Log-Jacobian determinant spatial maps - subject 08 to 16

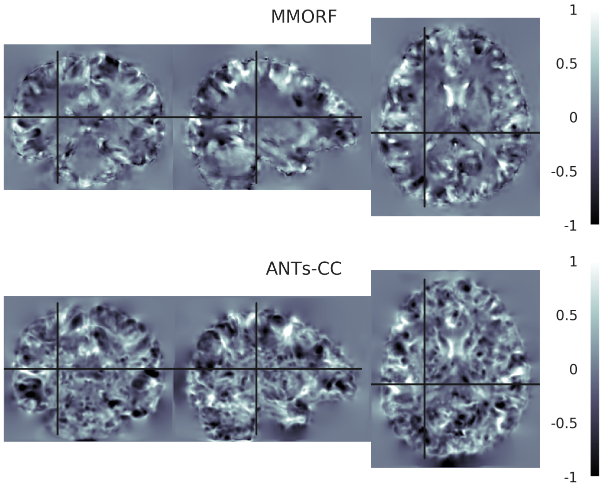

Log-Jacobian determinant spatial maps - subject 09 to 16

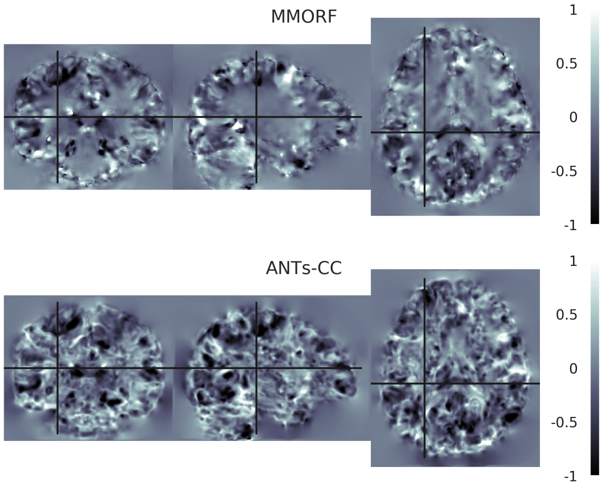

Log-Jacobian determinant spatial maps - subject 10 to 16

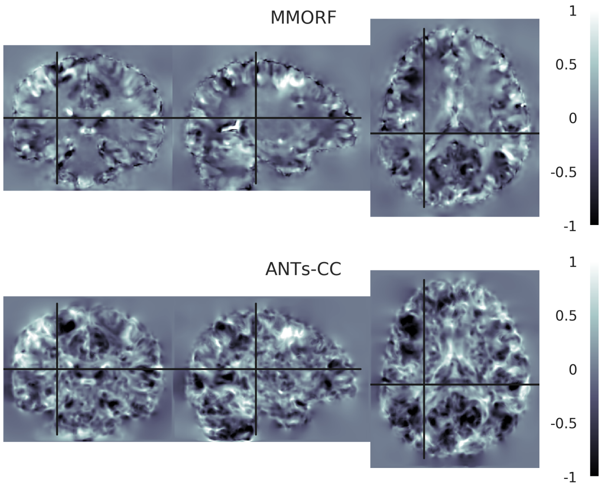

Log-Jacobian determinant spatial maps - subject 11 to 16

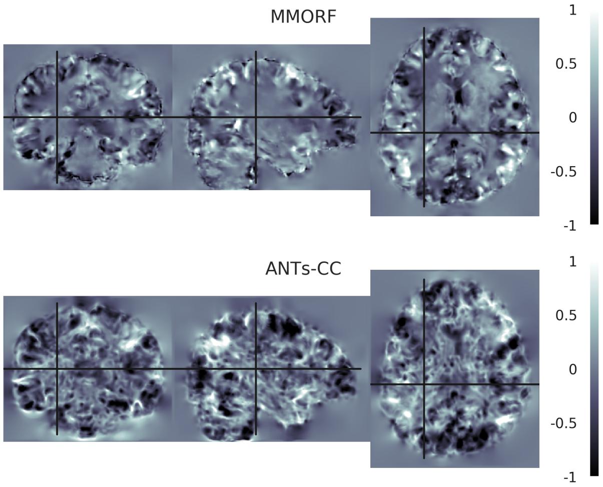

Log-Jacobian determinant spatial maps - subject 12 to 16

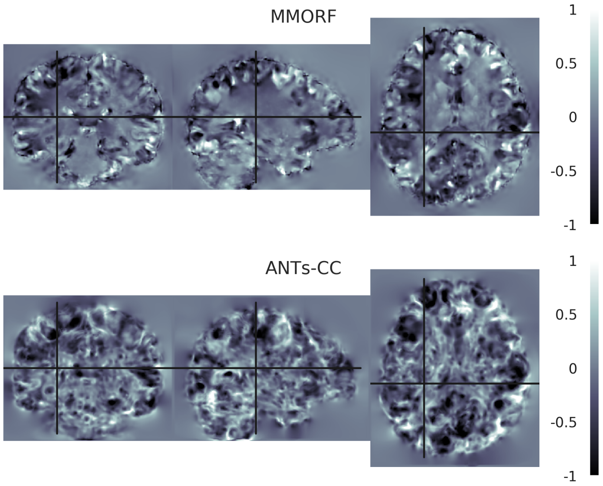

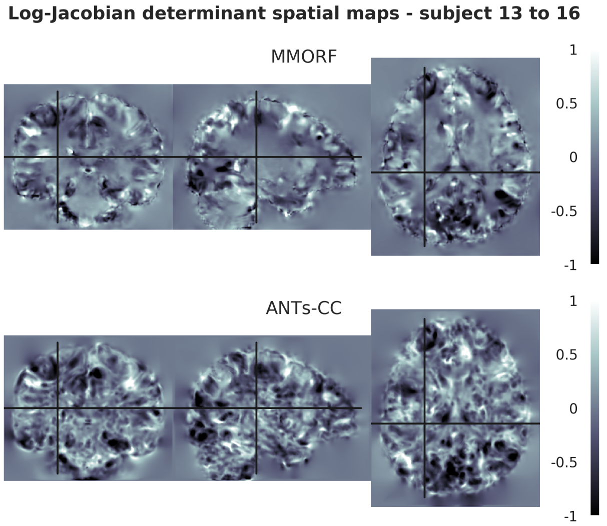
Log-Jacobian determinant spatial maps - subject 13 to 16
MMORF
ANTs-CC
1
0.5
0
-0.5
-1

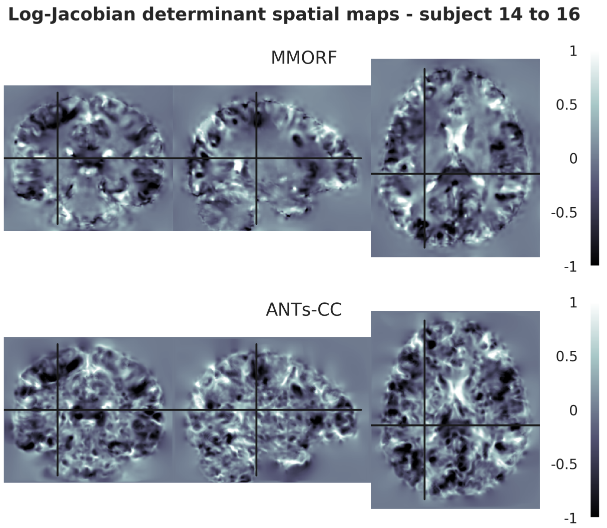
Log-Jacobian determinant spatial maps - subject 14 to 16
MMORF
ANTs-CC
1
0.5
0
-0.5
-1

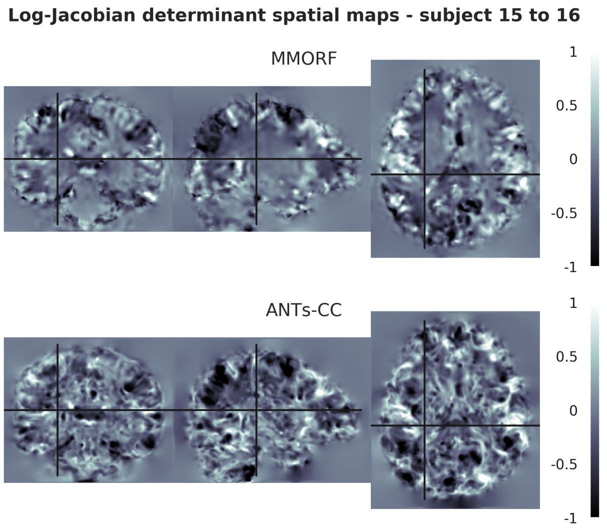
Log-Jacobian determinant spatial maps - subject 15 to 16
MMORF
ANTs-CC
1
0.5
0
-0.5
-1
