# Supplementary material for: A Symmetric Prior for the Regularisation of Elastic Deformations: Improved anatomical plausibility in nonlinear image registration
Source: Neuroimage. Author manuscript; Available in PMC 2021 May 17. (PMC7610794; doi:10.1016/j.neuroimage.2020.116962)
Supplement: Supplementary file 1 [file EMS123321-supplement-Appendix.pdf]

Appendix A. Approximation of the SPRED Penalty

In this section we provide the derivation of the approximation shown in Equation (11) of the main text, and provide an interpretation of this approximation in terms of a local rigidity penalty.

Appendix A.1. Approximation

Given:

$$\log^2 x \approx x + \frac{1}{x} - 2 \tag{A.1}$$

Let:

$$y(x) = x + \frac{1}{x} - 2 \tag{A.2}$$

And:

$$z(x) = \log x \tag{A.3}$$

$$\therefore x(z) = e^z \tag{A.4}$$

Then:

$$y(z) = e^z + e^{-z} - 2 \tag{A.5}$$

Now, taking the Taylor expansion of $y(z)$ about $z = 0$:

$$\begin{aligned} y(z)|_{z=0} &\approx y(0) + \frac{y'(0)}{1!}(z-0) + \frac{y''(0)}{2!}(z-0)^2 \\ &= 0 + 0 + z^2 \\ &= z^2 \end{aligned} \tag{A.6}$$

And finally, using Equation A.3:

$$\therefore y(x) \approx \log^2 x \tag{A.7}$$

Fig. A1 graphically demonstrates the relationship between the exact and approximate penalty functions.

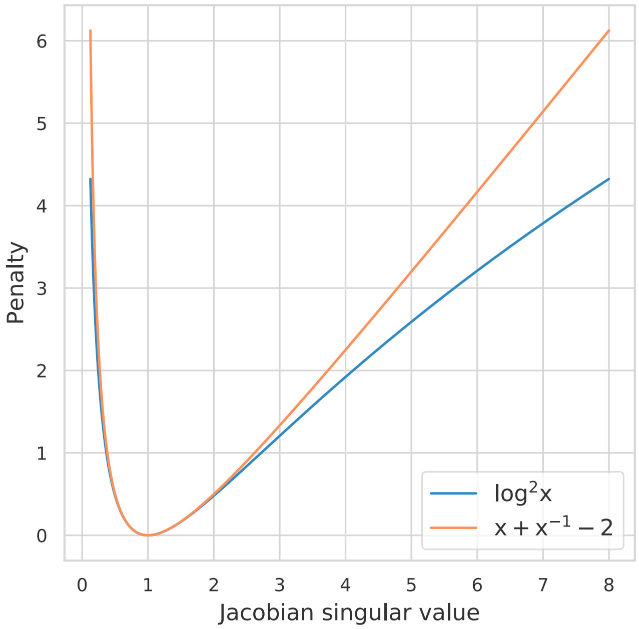

Fig. A.1. Exact vs approximate SPRED penalty - Effect of the approximation in Equation A.1 on the SPRED penalty. The approximation is almost indistinguishable from the exact penalty for singular values near 1. For singular values between 0.25 and 4 the approximation is accurate to within 17%, and within 42% between 0.125 and 8. Note that the approximation maintains the desirable symmetry for expansions and contractions. Note too that the approximation majorises the exact penalty (*i.e.*, the approximation is always greater than or equal to the exact penalty), and therefore maintains the exact penalty's ability to ensure diffeomorphism.

Appendix A.2. Interpretation

As shown in Equation (15) of the main text, utilising the approximation leads to a penalty in terms of the local Jacobian containing the term:

$$y = \mathrm{tr}\left(\mathbf{J}^T\mathbf{J} + (\mathbf{J}^{-1})^T\mathbf{J}^{-1} - 2\mathbf{I}\right) \tag{A.8}$$

Which may be rearranged as follows:

$$y = \mathrm{tr}\left(\mathbf{J}\mathbf{J}^T - 2\mathbf{I} + (\mathbf{J}^{-1})^T\mathbf{J}^{-1}\right) \tag{A.9}$$

$$= \mathrm{tr}\left(\mathbf{J}\mathbf{J}^T - \mathbf{J}\mathbf{J}^{-1} - (\mathbf{J}^{-1})^T\mathbf{J}^T + (\mathbf{J}^{-1})^T\mathbf{J}^{-1}\right) \tag{A.10}$$

$$= \mathrm{tr}\left(\left(\mathbf{J} - (\mathbf{J}^{-1})^T\right)(\mathbf{J}^T - \mathbf{J}^{-1})\right) \tag{A.11}$$

$$= \mathrm{tr}\left((\mathbf{J}^T - \mathbf{J}^{-1})^T(\mathbf{J}^T - \mathbf{J}^{-1})\right) \tag{A.12}$$

$$= ||\mathbf{J}^T - \mathbf{J}^{-1}||_F^2 \tag{A.13}$$

where $||\ ||_F^2$ is the squared Frobenius norm.

This penalty is minimised to 0 when $\mathbf{J}^{\mathrm{T}} = \mathbf{J}^{-1} \Leftrightarrow \mathbf{J}$ is an orthogonal matrix. This provides an interesting and intuitive alternative interpretation of the approximate SPRED penalty as one that penalises transformations for which the local deformation deviates from a pure rotation. Under that interpretation, the similarity to the *Local Rigidity* penalty

$$\left|\left|\mathbf{J}^{\mathrm{T}}\mathbf{J} - \mathbf{I}\right|\right|_F \tag{A.14}$$

as used by Loeckx et al. (2004); Staring et al. (2007) becomes apparent.

The primary difference between these two methods is that Equation A.13 includes $\mathbf{J}^{-1}$, and consequently tends to ∞ as $|\mathbf{J}|$ tends to 0. The SPRED approximation is therefore able to preserve diffeomorphism, whereas Local Rigidity is not.

Appendix B. 2D Example

We begin by defining a displacement warp field and its values in the underlying image space. The image space is a square with dimensions 21×21. The cubic B-splines are placed with their control points (knots) 5 samples apart. Each spline has a spatial support radius of 2 knots (10 samples), and therefore we place an extra ring of splines around the perimeter of the image space, such that each point in the image has an equal number of splines with support there. As a result, our *x*-warp and *y*-warp parameters are of dimension 7×7. We initialise the warp with a random set of parameters. Fig. B1 shows the warp parameters, the resulting warp in image space, and the gradient of the warp in image space.

Based on the spatial gradients of the displacement fields in Fig. B1, the local Jacobian matrix can be calculated at each point in space according to Equation (7).

The elements of $\mathbf{J}$ for each point in the sample space, as well as the resulting Jacobian determinant, $|\mathbf{J}|$, are shown in Fig. B2, which now contains all of the information necessary to calculate the voxelwise part (Equation (22)) of the calculations for the gradient (Equation (19)).

Based on the elements of $\mathbf{J}$, Fig. B3(a) shows the resulting contribution to the total cost from each point in the image. Fig. B.3(b) to B.3(e) then constitute the gradient of the cost with respect to the elements of $\mathbf{J}$ at each point in the image, i.e., the columns of $\left[\frac{\partial c}{\partial \mathbf{J}}\right]$ (Equation (22)). Each point in the four partial derivative images can be calculated completely independently of every other point. The calculation of these images therefore constitutes the first parallelised step of our algorithm.

We now move onto visualising the result of Equation (25). Note that Equation (24) is constant across the image and identical over the support of every spline. Therefore, when calculating the gradient in a parallel-per-parameter regime, the operation conforms exactly to the SIMD paradigm and may be very efficiently implemented on GPUs. In practice, we perform one such calculation for each combination of $\mathbf{J}$ element gradient image (Fig. B3(b) to B3(e)) and warp direction (*x* or *y*), the results of which are shown in Fig. B4(b) to B4(e). Finally, we sum over these combinations for each warp direction, and the result is then the gradient of our penalty with respect to our warp parameters. This is shown in Fig. B.4(f) and B.4(g).

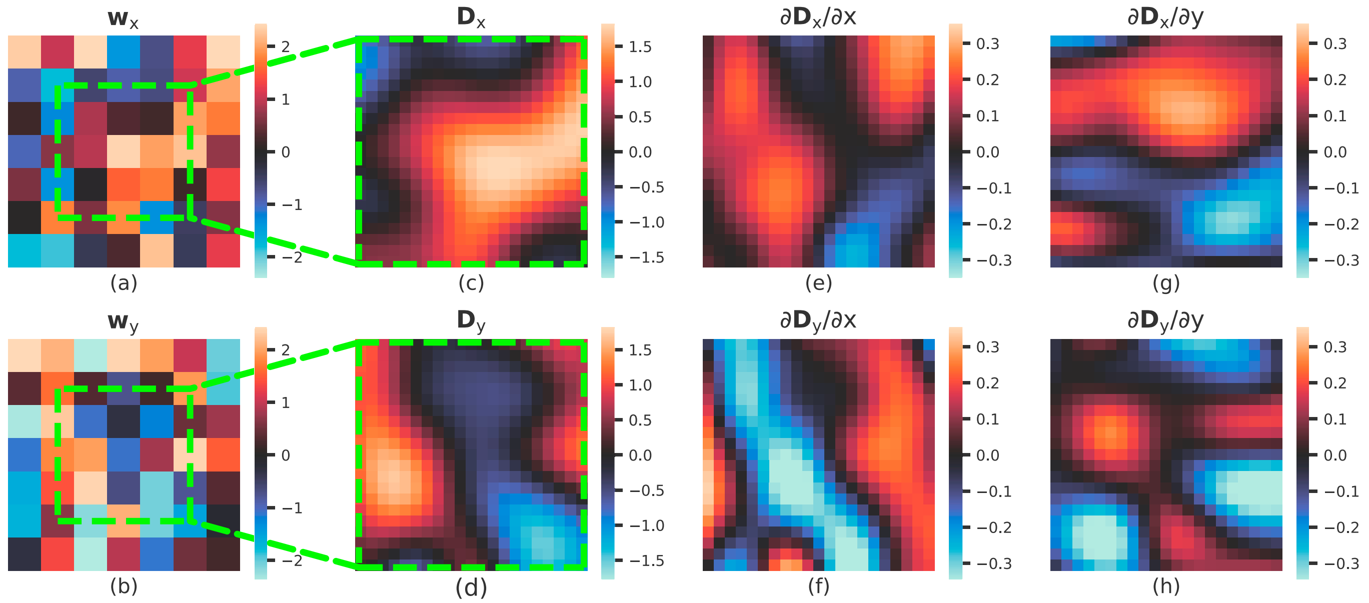

Fig. B.1. 2D B-spline displacement field and spatial derivatives - Example of a warp (or technically *displacement*) field for a random set of parameters (cubic B-spline coefficients). The positive *x*-direction is defined from left to right, and the positive *y*-direction from top to bottom. The *x*-warp parameters $\mathbf{w}_x$ (a) and *y*-warp parameters $\mathbf{w}_y$ (b) are arranged on a regular 7×7 grid. The resulting *x*-displacement field $\mathbf{D}_x$ (c) and *y*-displacement field $\mathbf{D}_y$ (d) corresponding to $\mathbf{w}_x$ and $\mathbf{w}_y$ are evaluated on a regular grid with knot spacing of 5×5. Note that only those parts of the warps with full support of the surrounding B-splines are shown (i.e., the area outside of the green square is considered to be *outside* of the image we are warping). This leaves a square of 21×21 samples in image space. The spatial partial derivatives of the *x*-warp in the *x*-direction (e), *y*-warp in the *x*-direction (f), *x*-warp in the *y*-direction (g) and *y*-warp in the *y*-direction (h) are presented, as these values are used in determining the local Jacobian matrices of the warp field. Cubic B-spline parametrisation ensures that both the warps and their spatial derivatives have analytical solutions and are smooth-continuous.

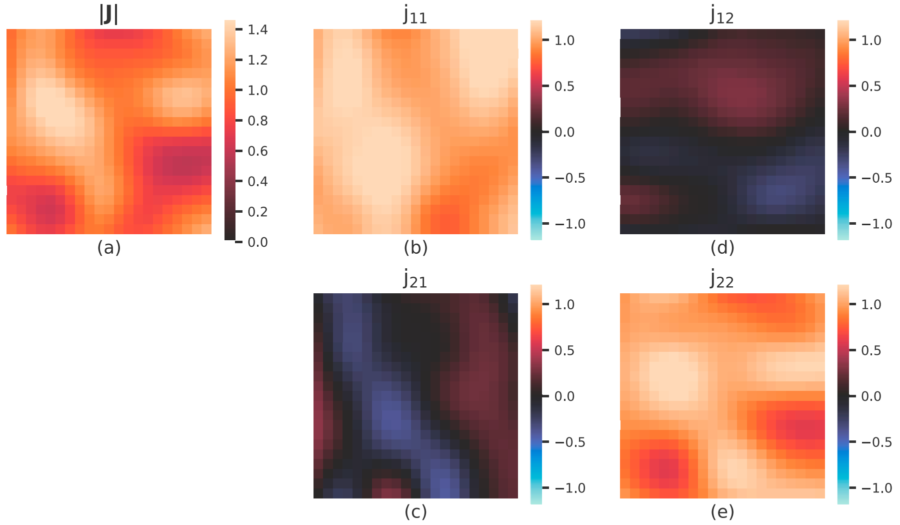

Fig. B.2. 2D Jacobian matrix fields - Elements of the local Jacobian matrix of the warp. At each position $[x,y]$ in image space a local Jacobian matrix **J** is calculated according to Equation (7) by adding the identity matrix I_2 to the 2×2 matrix composed of the elements of Fig. B1 (e)–(h) at that position. The resulting $2\times$ 2 2D fields (each showing how one element of **J** varies across the image) are presented here in sub-figures (b) to (e). From these a local Jacobian determinant field (a) can be calculated. The SPRED penalty is based on the singular values of **J**, and is therefore a function of fields (b) to (e).

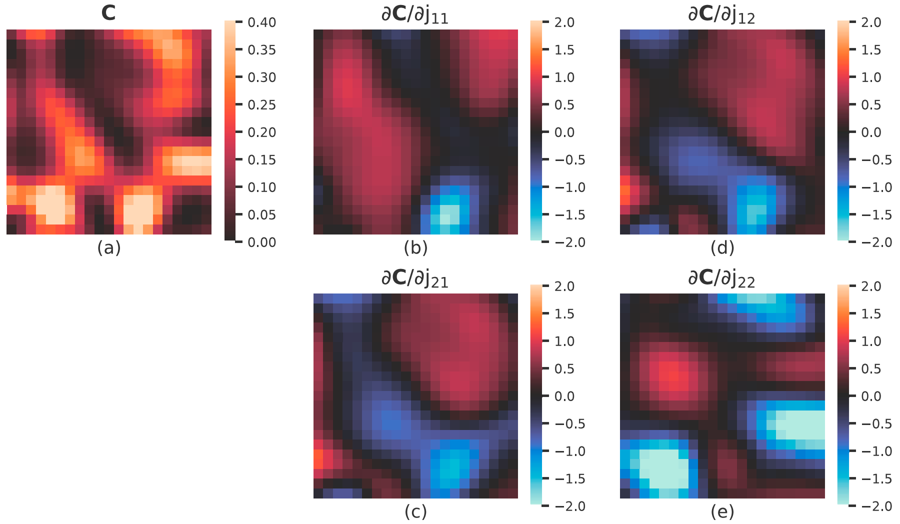

Fig. B.3. 2D cost field and Jacobian matrix gradient - From the singular values of the local Jacobian matrices of Fig. B2 a cost field **C** (a) can be evaluated at each position $[x,y]$ of the image. **C** then represents the contribution to the total cost from each point in image space. The partial derivatives of **C** with respect to the elements of **J** are shown in sub-figures (b) to (e). These partial derivative fields (which together make up the overall gradient $\frac{\partial \mathbf{C}}{\partial \mathbf{J}}$), can be calculated independently for each position $[x,y]$ as they depend *only* on the values of **J** at $[x,y]$, making this step ideal for a parallel-per-voxel approach.

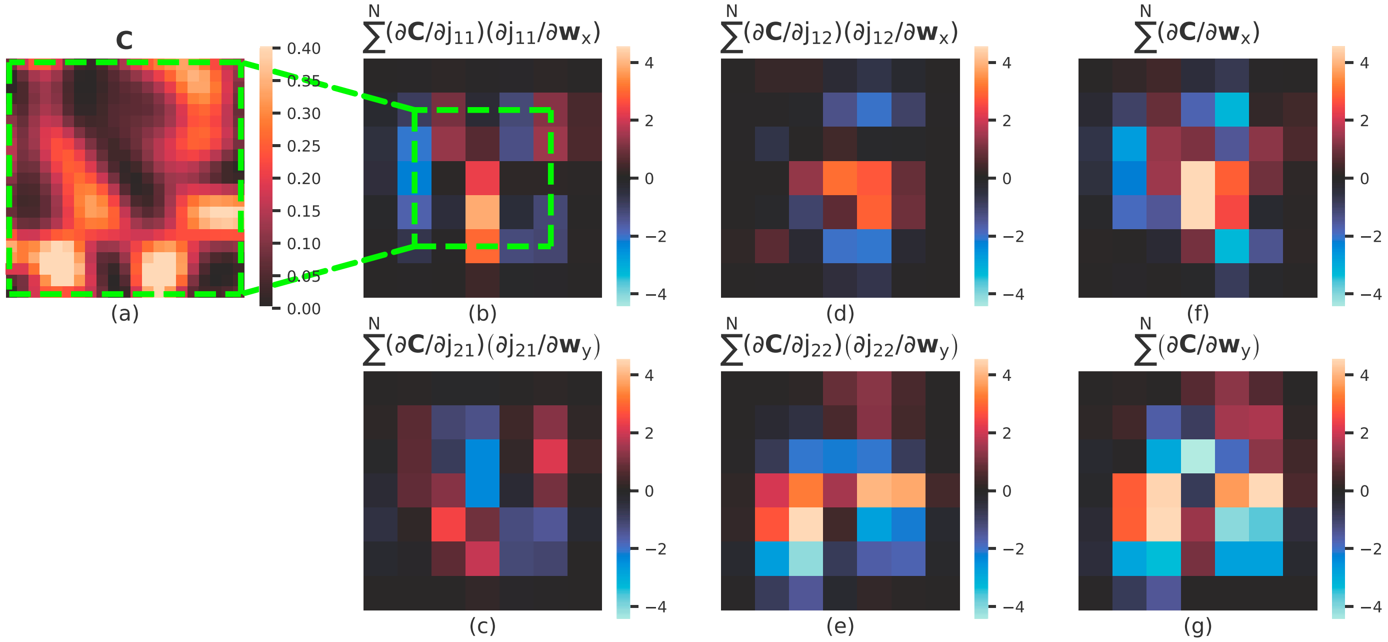

Fig. B.4. 2D cost field and warp parameter gradient - The final, and most computationally expensive, step is calculating the gradient of the total SPRED penalty (being the sum of **C** over all x and y) with respect to the warp parameters. Given that we have the gradient of **C** with respect to **J**, knowing the gradient of the resulting partial derivative fields (i.e., Fig. B3 (b)–(e)) with respect to the warp parameters would allow us to apply the chain rule to calculate this. We proceed by recognising that the piece-wise polynomial nature of B-splines means that the gradient of **J** with respect to each warp parameter is constant. Additionally, the gradient at position $[x, y]$ is exactly 0 for any B-spline which does not have support at that position. Thus, we can calculate the total gradient with respect to each warp parameter by summing the result of the chain rule across each position $[x, y]$ where that spline has support. In (b) to (e) we can see this illustrated in terms of the contributions to the overall gradient due to the effect of the warp parameters $\mathbf{w}_x$ and $\mathbf{w}_y$ on each element of the **J** fields. These partial derivatives can be calculated independently for each warp parameter, making this step ideal for a parallel-per-parameter approach. The end result of the process is then the gradient of the total penalty with respect to $\mathbf{w}_x$ (f) and $\mathbf{w}_y$ (g).

Appendix C. Parameter Selection

Parameter selection in image registration is non-trivial and often leads to discussions regarding whether or not the particular parameters selected were optimal. As such, the aim of parameter selection in this work was not necessarily to find the absolutely optimal set of parameters for each method, but rather to find parameters which made the various methods equal in one metric (such as accuracy in terms of overlap scores), in order so that differences in another metric (such as aggressiveness in terms of Jacobian determinant distributions) can be meaningfully compared.

Appendix C.1. MMORF

We fixed the number of steps in the warp resolution pyramid (40, 20, 10, 5, 2.5 and 1.25 mm isotropic), and then optimised over three parameters, namely:

- Smoothing as function of warp resolution (λ_s)
- Initial regularisation (λ_{ri})
- Rate of decrease of regularisation (λ_{rd})

The FWHM of the smoothing kernel was defined as the current warp resolution divided by λ_s. The initial regularisation λ_{ri} is in arbitrary units and its exact value is not of interest. The total regularisation at each step of the resolution pyramid was $\lambda_{ri} \times (\lambda_{rd})^{it}$ where *it* is the current position in the resolution pyramid (i.e. 0 to 5).

The parameter values were chosen via a grid search of the parameter space. Half of the NIREP dataset (8 subjects, 56 warps) were used, and overlap scores calculated as per Section 2.5.4. The parameters which resulted in the best overlap scores were then used for the rest of the analysis. The final parameters were:

$\lambda_s = 4.0$

$\lambda_{ri} = 0.15$

$\lambda_{rd} = 0.75$

These parameters are relatively aggressive, but as we were aiming for maximum overlap scores this is to be expected.

Appendix C.2. FNIRT

The parameters for FNIRT were chosen to match those used by the tool's author in (Andersson et al., 2019).

Appendix C.3. ANTs

For both ANTs-CC and ANTs-MSQ the number of steps in the resolution pyramid were fixed (10, 6, 4, 2 and 1 mm isotropic). Smoothing was chosen to match MMORF as closely as possible at each resolution level. This left three parameters over which to optimise, namely:

- Gradient step (λ_g)
- Update field variance (λ_u)
- Total field variance (λ_t)

$\lambda_g$determines how far each point is allowed to move on each iteration, and increasing this value allows for higher frequency deformations. λ_u determines how much to smooth the gradient field between updates and increasing this value increases smoothness in the velocity field. λ_t determines how much to smooth the total displacement field.

The parameters were selected such that the MSQ after registration was similar to that of MMORF. Note that despite our best efforts, we were unable to get ANTs-MSQ to match MMORF and ANTs-CC in terms of this metric. The best results for ANTs-MSQ were achieved with:

$$\lambda_g = 0.85$$

$$\lambda_u = 2$$

$$\lambda_t = 0$$

And for ANTs-CC with:

$$\lambda_g = 0.5$$

$$\lambda_u = 2$$

$$\lambda_t = 0$$

$\lambda_t$was found to always increase MSQ and was therefore left at the default value of 0. The final values for λ_g and λ_u are more aggressive than the default (fairly conservative) values of $\lambda_g = 0.1$ and $\lambda_u = 3$, but we found that this was necessary to achieve the required registration accuracy for a fair comparison.

Note also that the ANTs-CC parameters are very similar to those supplied by the tool's author for use in Klein et al. (2009).

Appendix D. Cube-Volume Aspect Ratio

To facilitate a quantitative comparison of registration aggressiveness we require metrics to measure changes in both volume and shape. The Jacobian determinant is useful in that it provides a single number quantifying volumetric changes. Similarly, we use the Cube-Volume Aspect Ratio (CVAR) to describe the deviation in shape of any cuboid from a regular cube (Smith and Wormald, 1998). CVAR in 3 dimensions is defined as: *The cube-root of the ratio of the volume of the smallest regular cube which can fully enclose the cuboid, to the cuboid's own volume*. Alternatively, for a deformation we may equally well define it as: *The cube-root of the ratio of the largest Jacobian singular value cubed, to the Jacobian determinant* (see Equation D.1).

$$CVAR = \sqrt[3]{\frac{s_{i-max}^3}{s_1 \times s_2 \times s_3}} \tag{D.1}$$

CVAR therefore takes on a value of 1 for a regular cube, and is greater than 1 for any other shape.

Appendix E. Alternative Regularisation Penalties within MMORF

Comparison of different regularisation penalties across registration frameworks is a difficult undertaking. It is not always possible to control all other potential sources of variability. For completeness, we therefore provide here a comparison between the SPRED penalty and two other related penalties, all implemented within the MMORF framework.

Appendix E.1. Jacobian Determinant Penalty

Given that we believe the Jacobian determinant is a good measure of warp aggressiveness, the question may arise as to why we prefer to penalise the Jacobian singular values rather than the determinant itself. Our reasoning, as stated in the main text, is that simply penalising the determinant will lead to implausible shape distortions in an attempt to minimise volumetric changes. To demonstrate this, we have implemented the following diffeomorphism preserving penalty based on the log-Jacobian determinant directly:

$$C_{LJ}(\vec{w}) = \sum_{n=1} v(1 + |\mathbf{J}_n|)\log^2|\mathbf{J}_n| \tag{E.1}$$

Appendix E.2. Local Rigidity Penalty

In Appendix A we show that our approximation to the SPRED penalty can be interpreted as promoting transformations which are locally rigid. However, the SPRED penalty is considerably more computationally complex than alternative methods which also promote local rigidity. We therefore implemented the following much simpler penalty in order to determine whether the additional complexity is warranted or not:

$$C_{LR}(\overrightarrow{w}) = \sum_{n=1}^{N} v \left\| \mathbf{J}_n^{\mathrm{T}} \mathbf{J}_n - \mathbf{I} \right\|_F \tag{E.2}$$

Appendix E.3. Testing

We registered all subjects of the NIREP dataset to subject NA01 only (i.e. 15 warps in total) using the three different regularisation methods.

The regularisation weighting was chosen such that the average overlap scores were comparable across all methods. We then calculated overlap scores as in Section 2.5.4 of the main text. For each warp we then calculated the minimum, maximum, mean, 5th and 95th percentile for both the Jacobian determinant and CVAR metrics. Overlap scores are presented in Fig. E1 and E2. Jacobian determinant ranges are presented in Fig. E3, and mean CVAR in Fig. E4. Spatial maps of both Jacobian determinants and CVAR are presented in Fig. E5 and E6. Finally, Fig. E7 shows a grid deformed under each regularisation scheme.

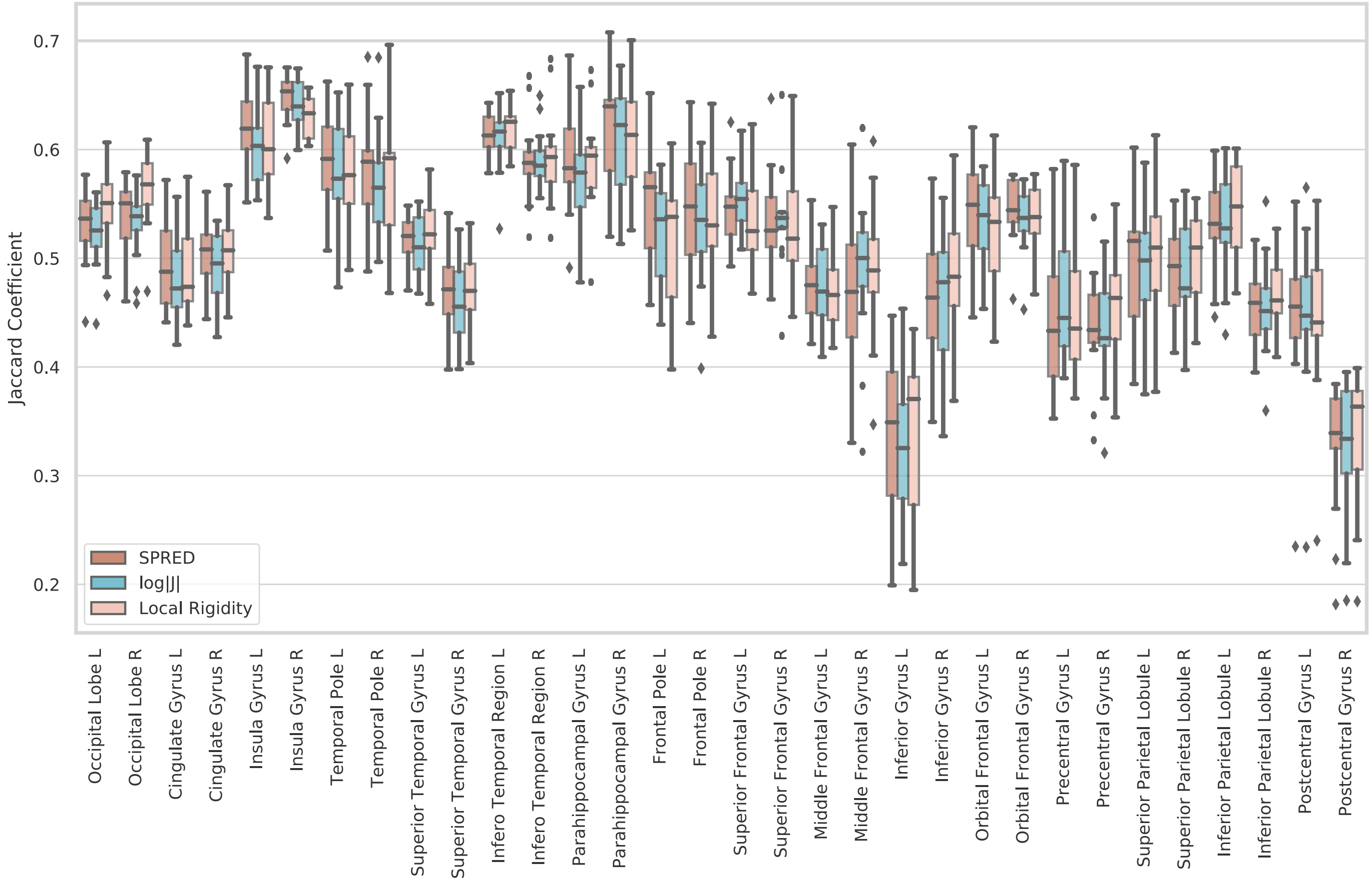

Fig. E.1. Jaccard coefficients for all subjects to NA01 - Jaccard coefficients of 32 cortical segmentations for registration to subject NA01 of the NIREP dataset. No one method was consistently superior or inferior across all regions leading to comparable results between the methods in terms of overlap scores.

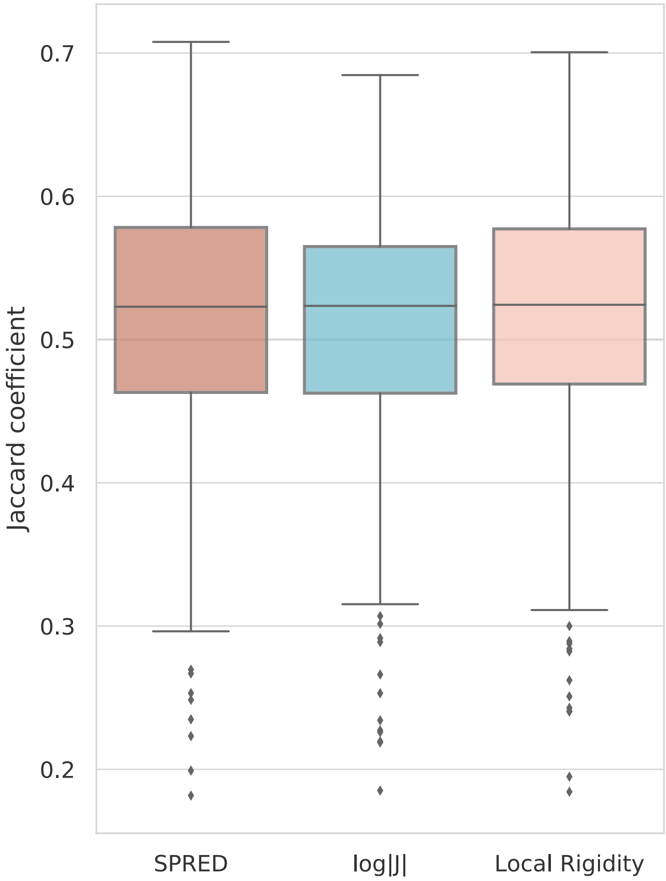

Fig. E.2. Comparative Jaccard coefficients - Distribution of Jaccard coefficients combined across all regions and subjects pairs for each regularisation penalty. This confirms what is seen in Fig. E1, that all methods have comparable overlap accuracy on average.

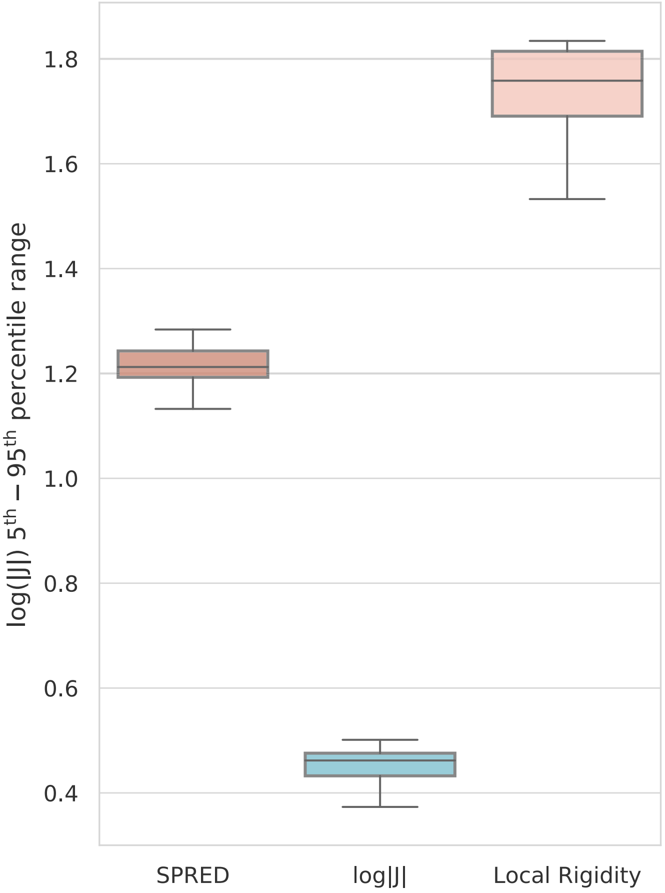

Fig. E.3. Log-Jacobian determinant range distributions - 5th to 95th percentile range of Jacobian determinants for warps from all subjects to NA01 of the NIREP dataset. As would be expected penalising the log-Jacobian determinant directly leads to a much tighter distribution of Jacobian determinants about 1 than penalising the Jacobian singular values. The local rigidity penalty performs worst in this regard, with consistently more extreme Jacobian determinants.

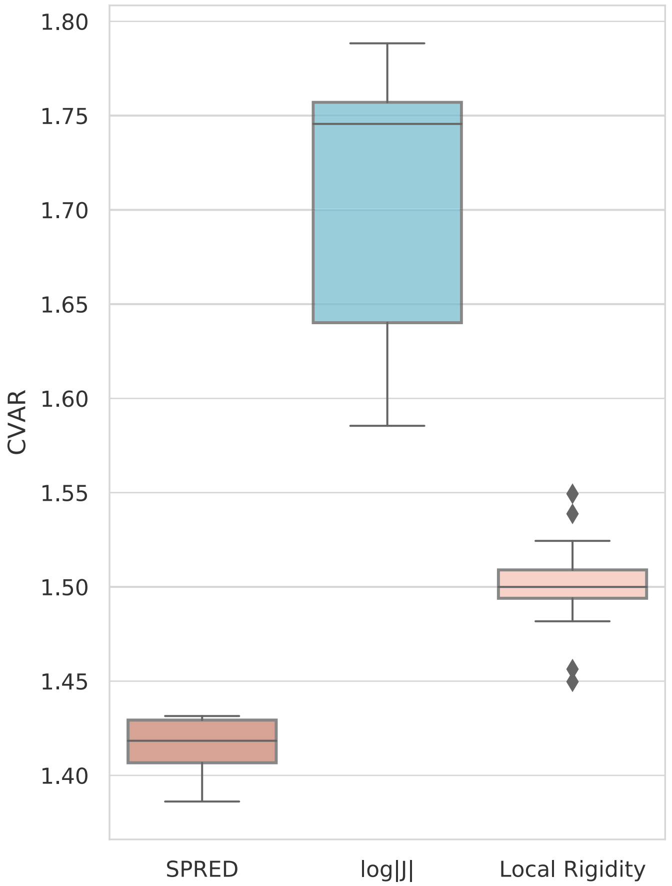

Fig. E.4. Mean CVAR distributions - Distribution of mean CVAR within the brain for warps from all subjects to NA01 of the NIREP dataset. We observe that SPRED clearly best preserves the original shape of the underlying anatomy during deformation. The local rigidity penalty, while not quite as good as SPRED, still consistently outperforms the log-Jacobian determinant penalty.

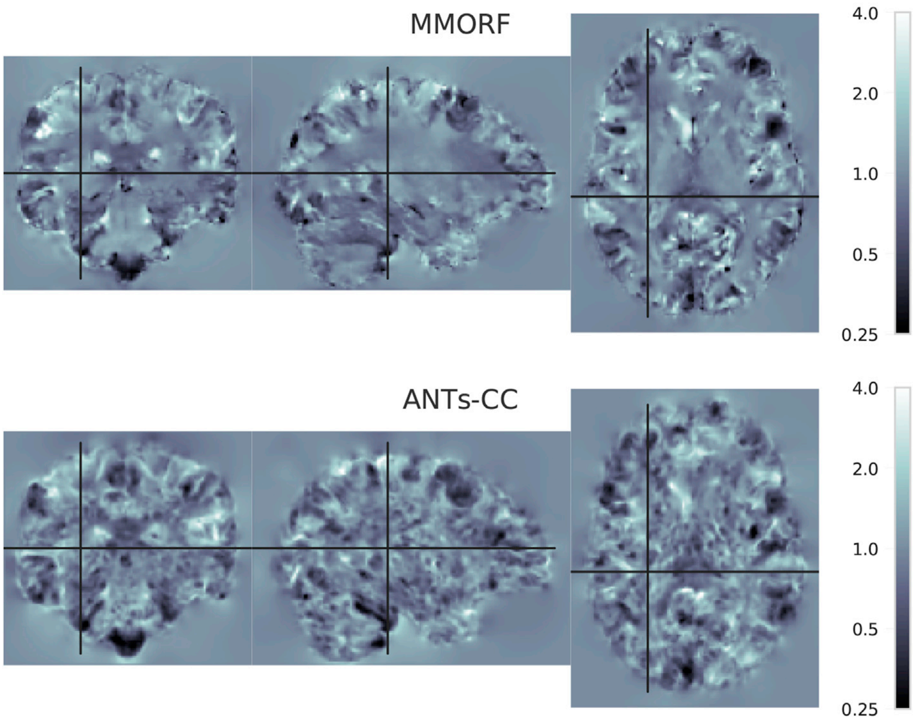

Fig. E.5. Jacobian determinant spatial maps - Spatial distribution of Jacobian determinants for SPRED, log|**J**|, and local rigidity regularisation. Penalising the volumetric changes directly clearly leads to the flattest Jacobian determinant maps. As the local rigidity penalisation does not guarantee diffeomorphism we observe a number of areas where the Jacobian determinant is either very close to 0, or negative, particularly in and around the cortex. SPRED is somewhere between the other two methods, which agrees with our observations in Fig. E3.

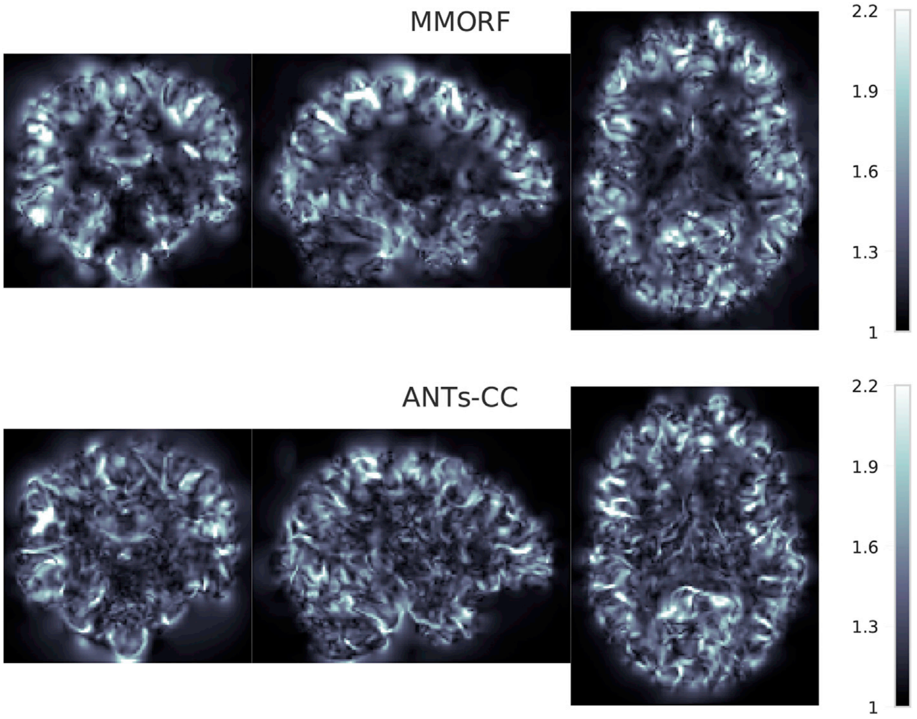

Fig. E.6. CVAR spatial maps - Spatial distribution of CVAR values for SPRED, log|**J**|, and local rigidity regularisation. It is immediately clear that the flat Jacobian determinant maps for the log|**J**| penalty are accompanied by a marked increase in CVAR, confirming that volumetric control has come at the cost of extreme shape changes. Local rigidity penalisation leads to excellent preservation of shape within the white matter, but shows extreme distortion within some areas the cortex. SPRED performs considerably better than either of the other methods, confirming our observations in Fig. E4.

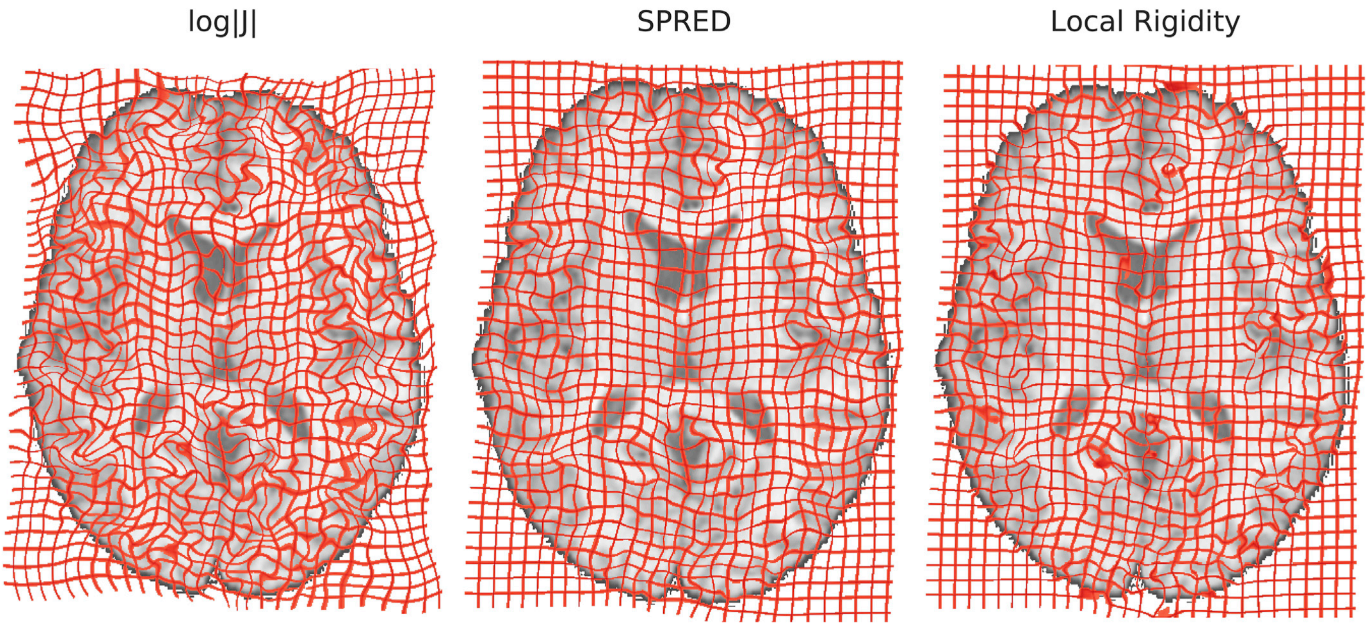

Fig. E.7. Comparison of deformed grids - Warped grid showing the effect of the different regularisation penalties. Penalising the Jacobian determinant alone has led to extremely convoluted warps, whereas penalising the Jacobian singular values results in smoothly varying warps which are far more plausible. Interestingly, penalising local rigidity has led to a very similar deformation to SPRED, with the exception of areas where topological folding or collapse has occurred. These areas are particularly evident within the cortex and at tissue boundaries.

E.4. Discussion

From Figs. E1 and E2 we see that the choice of regularisation metric need not affect the overall registration accuracy. From Fig. E3 we note that, as expected, penalising the Jacobian determinant directly leads to warps with less volumetric changes as compared to both SPRED and local rigidity. However, Fig. E4 shows that this comes at the cost of increased shape distortion as measured by CVAR, where both SPRED and local rigidity are far more adept at controlling implausible shape changes.

Fig. E5 and E6 provide a clearer understanding of the information in Fig. E3 and E4. The extent to which volumetric and shape changes are traded off by the different regularisation schemes is clearly evident. Finally, from Fig. E7 we see the extent to which extreme CVAR and Jacobian determinant values translate into more convoluted warps with reduced spatial smoothness as compared to SPRED.

By combining the log-Jacobian determinant and local rigidity penalties, in a similar manner to Staring et al. (2007), it may be possible to overcome the limitations of each penalty. However, this would introduce additional regularisation weights to tune. In this respect the SPRED penalty is far more elegant, as it effectively controls both volumetric and shape changes, preserves topology, and requires only a single tuning parameter.

In conclusion, this example shows that simply penalising the Jacobian determinant is insufficient to ensure plausible deformations, and that the additional complexity of SPRED compared to a simpler local rigidity penalty is justified.

Appendix F. Supplementary data

Supplementary data to this article can be found online at https://doi.org/10.1016/j.neuroimage.2020.116962.
